# Between the scales and the scars: The interpersonal moderators of the association between Body Mass Index and parent-reported depression/anxiety diagnoses in U.S. children: Evidence from the 2021–22 National Survey of Children’s Health (NSCH)

**DOI:** 10.1371/journal.pgph.0006462

**Published:** 2026-05-20

**Authors:** Fnu Kajal, Bin Suh, Chen Zhao, Maia Ingram, Scott Carvajal, Karl Krupp, Purnima Madhivanan

**Affiliations:** 1 Mel & Enid Zuckerman College of Public Health, University of Arizona, Tucson, Arizona, United States of America; 2 Arizona Department of Health Services, Tucson, Arizona, United States of America; 3 Epidemiology and Biostatistics, Mel & Enid Zuckerman College of Public Health, University of Arizona, Tucson, Arizona, United States of America; 4 Arizona Prevention Research Center, Mel & Enid Zuckerman College of Public Health, University of Arizona, Tucson, Arizona, United States of America; 5 Health Promotion Sciences, Mel & Enid Zuckerman College of Public Health, University of Arizona, Tucson, Arizona, United States of America; 6 Division of Public Health Practice & Translational Research, Mel & Enid Zuckerman College of Public Health, University of Arizona, Tucson, Arizona, United States of America; 7 Health Promotion Sciences, Mel & Enid Zuckerman College of Public Health, University of Arizona, Tucson, Arizona, United States of America; Islamic Azad University South Tehran Branch, IRAN, ISLAMIC REPUBLIC OF

## Abstract

Adverse childhood mental health outcomes have emerged as a significant public health concern, with an increasing body of evidence linking children’s obesity with increased risk of mental health outcomes. This is a cross-sectional study of 40,820 U.S. children aged 6–17 used survey-weighted logistic regression on the 2021–22 National Survey of Children’s Health (NSCH) data. The study examined whether interpersonal factors such as bullying, poor mothers’ or fathers’ mental health, poor family resilience and greater difficulty in making or keeping friends modified the associations between BMI category and parent-reported lifetime depression (LDD) or anxiety (LDA) mental health outcomes, using interaction terms and adjusting for age, sex, race/ethnicity and federal poverty levels. Obesity was significantly associated with higher odds of both **LDD** and **LDA** in children aged 6–17 years. Obese children had **2.08 times higher odds** of LDD (aOR 2.08; 95% CI: 1.64-2.64) and **1.48 times higher odds** of LDA (aOR 1.48; 95% CI: 1.25-1.76) compared to normal-weight peers. Significant effect modification in the statistical model was observed for **LDD:** never bullied (aOR = 2.12; 95% CI: 1.45-3.11), mother’s mental health (aOR = 1.70; 95% CI: 1.70- 2.70), and father’s mental health (aOR = 2.54; 95% CI: 1.51-4.29). For **LDA**, effect modification in the statistical model was seen for **making friends** (aOR = 1.91; 95% CI: 1.13-3.22) and **father’s mental health** (aOR = 2.14; 95% CI: 1.34-3.41). In this cross-sectional sample, obesity was associated with higher LDD and LDA, with associations varying by interpersonal and family factors. Given parent-reported, cross-sectional data, findings are associative and not causal. The results emphasize integrating mental health screening into obesity programs, strengthening anti-bullying efforts, and adopting family-centered approaches; future research should use longitudinal, multi-informant designs to establish temporal relationships and reduce bias.

## Introduction

Childhood obesity and mental health outcomes have emerged as a pressing public health concern with significant implications for both physical and mental well-being. On a global scale, childhood obesity has proliferated at an unprecedented rate in recent decades. According to the World Health Organization (WHO), it was estimated in 2022 that more than 390 million children aged 5–19 were classified as overweight, and 160 million were obese [1]. In the United States, nearly 20 percent of children experience obesity, and the rates of childhood obesity have increased fourfold since 1990 [2], forming what is increasingly recognized as a “Body–Mind Burden” [3,4]. Concomitantly, the incidence of mental health outcomes in children has spiked, with nearly 50% of adolescents experiencing at least one mental illness, and emergency visits for mental health outcomes rising by 37% in recent years [5,6].

This epidemiological convergence is not coincidental. There is increasing evidence for a bidirectional relationship between obesity and mental health. Children with excess weight are more likely to experience depressive symptoms, parent-reported lifetime diagnosis of anxiety (LDA), and social withdrawal**,** as shown across multiple meta-analyses [7–10]. The reverse also holds: adolescents with parent-reported lifetime diagnosis of depression (LDD) have a 70% increased risk of obesity, particularly among females [11,12]. Luppino et al. established this reciprocal risk in a seminal meta-analysis, finding that obesity heightens LDD risk, and vice versa [13].

Although biological and behavioural determinants (e.g., dietary intake and physical inactivity) play a role in these associations, they do not fully account for the emotional distress presented by children with obesity. Instead, the observed associations may be socially patterned or mediated, although causal pathways cannot be established in cross-sectional data. Teasing/Bullying, stigma, parental mental health, family routines, and peer dynamics are increasingly implicated as effect modifiers in this relationship [14–16]. Yet, few studies have examined these interpersonal and family-context factors together within a single nationally representative analytic framework.

Among these, bullying emerges as an exceptionally robust interpersonal risk factor.

Children with obesity have up to 46% higher odds of being bullied, compared to their healthy-weight peers [17].

***According to the standards established by the World Health Organization (WHO), obesity is defined as a measurement that surpasses two standard deviations above the WHO growth reference median (World Health Organization [WHO], 2024). The Centers for Disease Control and Prevention (CDC) defines childhood obesity as having a body mass index (BMI) that is at or exceeds the 95th percentile for age and sex (CDC US Government, 2024).***

Victimization, especially related to weight, can exacerbate internalized stigma and trigger depressive and anxious symptomatology [18,19]. Longitudinal studies show that the mental health impact of obesity is significantly magnified among children who are bullied [20,21]. Additionally, children classified as bully-victims experience the highest rates of LDD, suicidal ideation, and behavioural problems [22,23]. Although bullying is widely acknowledged as linked to obesity and mental health outcomes, few studies have analyzed bullying together with other interpersonal and family factors within a single framework, especially using nationally representative data.

Peer relationships more broadly also serve as salient moderators. Quality friendships and strong peer attachment buffer children against the psychological impact of social exclusion due to obesity [24,25]. Increasing evidence indicates that secure peer attachment is protective for emotional health and well-being [26], whilst peer rejection evokes distress both within and outside the context of overt bullying [27,28].

In the familial sphere, parental mental health—particularly maternal LDD—has consistently been linked to both child obesity and mental health outcomes. Maternal LDD influences parenting styles, feeding practices, emotional availability, and home routines, all of which mediate the pathway from obesity to poor emotional outcomes [29–31]. The risk is heightened with chronic depressive symptoms, as shown in cohort studies and meta-analyses [32,33]. However, paternal mental health remains underexplored, and few studies have disaggregated effects by caregiver role or gender [34,35]. The impact of paternal mental health remains relatively understudied**,** with limited analyses distinguishing effects across different caregiver roles. Emerging evidence indicates that fathers’ psychological well-being may have unique influences on a child’s emotional development.

Family resilience—defined as the ability of families to adapt positively to adversity—has also emerged as a protective factor. Findings from studies that used the National Survey of Children’s Health (NSCH) data have shown that family resilience can attenuate the deleterious effects of bullying and adversity on internalizing symptoms [36,37]. For example, a study using data from the COVID-19 pandemic revealed that family functioning was positively related to adolescent LDA and LDD [38,39]. Yet, its role in buffering the psychological consequences of obesity has been underexplored, with few studies testing interaction effects in nationally representative datasets [40,41].

Overall, previous research indicates that obesity might interact with the social environment to influence children’s emotional health, but few studies have simultaneously explored bullying, peer relations, parental mental health, and family resilience as factors moderating this link. To fill this gap, this study utilizes nationally representative NSCH 2021–22 data and a socio-ecological approach to examine how these interpersonal elements impact the association between Body Mass Index (BMI) and LDD/LDA diagnoses in children aged 6–17.

## Theoretical framework

Mental health conditions in children are multifactorial, necessitating multidimensional theoretical frameworks. This research is grounded in two complementary frameworks—the socio-ecological model (SEM) and the diathesis-stress model—which explain how individual predispositions intersect with interpersonal and contextual stressors to affect mental health.

The **SEM**, originally proposed by Bronfenbrenner and expanded by McLeroy et al. into five levels of influence—Intrapersonal, Interpersonal, Institutional, Community, and Public Policy—underscores the dynamic interrelations between individuals and their environments [42,43]. While intrapersonal factors like sleep and activity patterns are essential, interpersonal stressors such as bullying, parental mental health, peer isolation, and family resilience are increasingly recognized as critical modifiers of mental health outcomes in children with obesity.

The diathesis-stress model dovetails with this ecological lens and explains how genetic and physiological predispositions intersect with environmental adversities to create mental illness [44,45]. We view obesity in this framework as a biological condition and psychosocial stressor that is experienced with stigma and social rejection. Nonetheless, these adversities may be ameliorated by protective interpersonal factors (e.g., emotionally balanced parenting and positive peer support). In tandem, such models provide a comprehensive framework for understanding how relational life experiences impact the emotional consequences of childhood obesity.

## Methodology

### Data source

The data for this study were obtained from the Child and Adolescent Health Measurement Initiative (CAHMI)/NSCH for the 2021–2022 period, which is publicly available [46]. The NSCH, sponsored by the U.S. Department of Health and Human Services (HHS), is conducted by the Census Bureau and has been providing nationally representative data on children’s health since 2002–2003.

### Study design

This study is a secondary data analysis of a cross-sectional survey examining the association between childhood obesity and mental health outcomes, particularly LDD and LDA. It focuses on identifying key interpersonal determinants that modify the association between obesity and mental health outcomes in children.

### Study population

The study population consists of children and adolescents aged 6–17, as reported in the 2021–2022 NSCH survey [47]. This diverse and nationally representative sample highlights a critical developmental period where obesity and mental health intersect, making it an ideal setting for investigating these issues and ensuring credible, actionable data to support research objectives.

The current research represented a sample size of 40,820 children, providing nationally representative data on their health and well-being. Data collection occurred between June 2021 and January 2022. The participants were the parents and guardians of children. The data were collected via the web (90%) and mail (10%), with an approximate response rate of 65%.

**Data Collection Tools/Measures:** All primary study variables were reported by the same parent or guardian respondent, including child height and weight, child LDD/ LDA diagnosis, bullying exposure, peer difficulties, family resilience, and parental mental health [47]. This introduces the possibility of same-informant bias and common method variance, whereby respondent characteristics, perceptions, or mental health status may influence reporting across multiple constructs in the same direction. For example, a parent experiencing psychological distress may be more likely to perceive or report difficulties in the child’s emotional functioning, peer experiences, or family context. Accordingly, the observed associations may partly reflect shared reporting processes rather than independent underlying phenomena. Although NSCH parent-reported items have shown acceptable utility in prior population-based research, this limitation is important, and findings should be interpreted cautiously. However, many previous studies on NSCH data have indicated the acceptable validity of parent-reported measures [48,49].

**Outcomes (Mental Health Measures):** The outcome variables were parent-reported lifetime diagnosis indicators for depression (LDD) and anxiety (LDA) available in the NSCH for children aged 6–17 years [50]. Regarding LDD and LDA, responses encompass the current diagnosis and historical data for children aged 6–17. The NSCH distinguishes current diagnosis from ever-diagnosed status. For the primary analysis, we combined current diagnosis with ever-diagnosed status to maximize statistical power, creating binary indicators of LDD and LDA. LDD/ LDA refers to children who have ever been diagnosed with depression/anxiety, including those who are currently depressed, and those who were previously diagnosed but no longer meet the criteria. We combined current and those who had ever been diagnosed as one category, and those who are not currently experiencing depression or anxiety as another category, for this comparison. We recognize that this approach introduces temporal ambiguity because current may not correspond to the timing of a past diagnosis. To evaluate the robustness of findings, we conducted sensitivity analyses using current diagnosis outcomes separately and report those results in the Supplement (S2 Table).

**Explanatory Variable (BMI):** Obesity was measured using BMI based on parent-reported height and weight, categorized according to CDC guidelines (CDC, 2016) into four groups by age-adjusted percentile: Underweight (less than 5^th^ percentile), Normal weight (5^th^ to 84^th^ percentile), Overweight (85^th^ to 94^th^ percentile), and Obese (95^th^ percentile or above)

**Covariates:** Covariates included the child’s age, sex, race/ethnicity, and federal poverty levels (FPL), which represent the socioeconomic status of the family. The question on the FPL is: “What is the income level (federal poverty level, FPL) of the household that this child lives in? The responses are divided into 0–99% FPL; 100–199% FPL; 200–399% FPL; 400% FPL or greater.

**Moderators:** These include interpersonal factors that reflect social relationships and peer interactions.

Bullying was assessed by asking respondents to report how often the child experienced bullying in the past 12 months. For the purpose of analysis, the responses were categorized into three categories: levels: “Never in past 12 months” = Never; “1-2 times in past 12 months” and “1-2 times per month,” into Occasional and “1-2 times per week” and “Almost every day” into Frequent. This single-item bullying measure has been used in all NSCH cycles and aligns with CDC Youth Risk Behavior Surveillance (YRBS) definitions of bullying. Prior NSCH documentation confirms the content validity and reliability of this item for population monitoring.

Making or keeping friends involved respondents, indicating the child’s level of difficulty in forming and maintaining friendships. The responses provided for this evaluation included “No difficulty,” “A little difficulty,” and “A lot of difficulty.” This indicator originates from the Strengths and Difficulties Questionnaire (SDQ) peer problems subscale, which has demonstrated construct validity and good internal consistency (α ≈ 0.70) in U.S. samples.

Father’s/Mother’s mental health was evaluated by asking respondents to assess the mental health status of the father/mother, if he/she was the primary caregiver. The assessment options included “Excellent/very good”, “Good”, and “Fair/poor.” This global self-rated item has been validated against standardized screening tools, such as the PHQ-2 and K6, showing a moderate to strong correlation in U.S. population surveys. Because parental mental health was both a substantive family-context variable and a characteristic of the same respondent source, it may also contribute to differential reporting of child outcomes and exposures.

Family resilience was evaluated by respondents who assessed the family’s ability to cope during difficult times. The question asked was: Does this child live in a home where the family demonstrates qualities of resilience during difficult times? The options included “All or most of the time to 0-1 items”, “All or most of the time to 2-3 items”, “All or most of the time to all 4 items.” These items comprise the NSCH Family Resilience Scale, which demonstrates strong internal consistency (Cronbach’s α = 0.80) and established construct validity.

All moderator items are part of the validated NSCH item bank maintained by the CAHMI. Psychometric properties of these scales have been described in prior technical documentation and peer-reviewed analyses [51,52].

An NSCH non-response bias analysis showed no evidence of bias. We applied weights, stratification, and accounted for clustering to enhance generalizability to the U.S. population of children and provided an unbiased test of statistical significance. The analytic sample included only children with complete responses on BMI, LDD, and LDA questions. Children under 6 years were excluded to avoid sensitivities related to BMI calculations and varying developmental stages. Of the original 64,535 children aged 6–17 years, we excluded 23,715 cases with non-analytic codes (95 were less than 2, 3 or 5 years old; 99 were missing or refused) for essential study variables. A complete-case analysis approach was used because missingness was primarily due to non-analytic codes rather than item non-response, and survey documentation indicated no systematic bias in the missingness. The ultimate analytic sample comprised 40,820 children with valid information on all variables of interest. We compared the excluded and retained participants in terms of age, sex, race/ethnicity, and family income (S1 Table); To assess potential selection bias due to exclusion criteria, demographic characteristics of retained and excluded participants were compared using survey-weighted chi-square tests). An inspection of the missing data pattern revealed no residual missingness and no need for multiple imputation.

### IRB and ethics statement

These survey results are openly available and de-identified; therefore, secondary analysis of the results is, according to federal guidelines, exempt from consideration by an Institutional Review Board (IRB).

### Analysis

The analysis used the R program and survey-weighted statistical procedures to address the NSCH’s complex sampling design, correcting for oversampling, non-response, and stratification issues. The NSCH data included weights that adjust for unequal selection probabilities, non-response, and post-stratification to accurately represent the population, as per the NSCH survey codebook [47].

The descriptive analysis includes both unweighted and weighted frequencies and percentages across demographic subgroups. A bivariate analysis examined association between BMI categories and LDD and LDA, as well as interpersonal variables. Design-based chi-square tests identified statistically significant associations. LDD or LDA were analyzed across demographic and interpersonal variable categories, as well as BMI categories, for statistical associations. Survey-weighted logistic regression models were used to evaluate the association between BMI and parent-reported lifetime diagnosis of depression (LDD) and anxiety (LDA). Models were adjusted for age, sex, and race/ethnicity, with federal poverty level (FPL) additionally included in the LDD model given its stronger theoretical and empirical relevance to depression outcomes and its contribution to model fit. Interaction terms between BMI and interpersonal variables were tested, and all moderator–BMI interactions were pre-specified based on the socio-ecological framework. Adjusted odds ratios (aOR) and 95% confidence intervals (95%CI) are reported, with statistical significance set at p < 0.05. Survey design variables, including weights, strata, and primary sampling units, were incorporated using the svyglm() function in R, with models defined using a quasibinomial family to address overdispersion in binary outcomes. Given mild overdispersion in the binary outcomes, we used quasibinomial logistic models to obtain robust variance estimates. Multicollinearity was assessed using variance inflation factors (VIF < 2 for all variables).. Stratified interaction analyses were conducted as exploratory and hypothesis-generating. We did not apply formal corrections for multiple comparisons; therefore, these results should be interpreted with caution regarding type I error risk.

## Results

In this study, the sample (Table 1), represented approximately 29 million children in the United States. The sample was evenly distributed among children aged 6–11 (49.29%), and those aged 12–17 years (50.70%). Similarly, the sample was balanced between male (50.93%) and female (49.06%) participants. While 57.29% were non-Hispanic White, 7.54% were non-Hispanic Black, and 11.62% reported other/multiracial. Furthermore, it was observed that 60.48% of the children had a normal BMI, while 15.07% were classified as overweight, and 15.28% obese. An LDA was reported in 13.29% of the participants, whereas an LDD occurred in 5.80%. In terms of interpersonal variables, 63.84% of children reported never being bullied within a year. For children who were bullied, 26.61% were bullied once or twice within the past year, with 5.75% bullied once or twice per month, and lower percentages bullied several times (2.69% per week, 1.05% per day). Additionally, 4.23% of the children indicated difficulties in making friends. Notably, 4.47% and 5.95% of children’s parental guardians (father/male and mother/female, respectively) exhibited poor mental health status. Lastly, only 5.15% of families demonstrated characteristics of full resilience across all four assessed items.

**Table 1:** Descriptive Stats (n = 40820) of children 6-17 years in the 2021-2022 NSCH Survey.

Variable	Categories	Unweighted frequency	Weighted frequency (in millions)
			**(Weighted %)**
Age	6-11 years	19108	14.39 (49.29%)
	12-17 years	21712	14.80 (50.70%)
Sex	Male	21205	14.87 (50.93%)
	Female	19615	14.32 (49.06%)
Race/Ethnicity	Hispanic	5072	6.87 (23.53%)
	White, Non-Hispanic	28911	16.73 (57.29%)
	Black, Non-Hispanic	1445	2.20 (7.54%)
	Other/Multiracial, non-Hispanic	5392	3.39 (11.62%)
FPL*	0-99%	3132	3.11 (10.65%)
	100-199%	5161	4.73 (16.22%)
	200-399%	12206	9.04 (30.97%)
	400% or greater	20321	12.30 (42.14%)
BMI	UW	3613	2.67 (9.14%)
	Normal	25952	17.66 (60.48%)
	Overweight	5806	4.40 (15.07%)
	Obese	5449	4.46 (15.28%)
Parent-reported LDA	No LDA	34173	25.31 (86.70%)
	LDA	6647	3.88 (13.29%)
Parent-reported LDD	No LDD	38434	27.50 (94.19%)
	LDD	2386	1.69 (5.80%)
INTERPERSONAL FACTORS			
Bullying	Never in past 12 months	24129	18.64 (63.84%)
	1-2 times in past 12 months	11959	7.77 (26.61%)
	1-2 times per month	2874	1.68 (5.75%)
	1-2 times per week	1281	0.78 (2.69%)
	Almost everyday	577	0.31 (1.09%)
Making or Keeping friends	No difficulty	30212	22.29 (76.35%)
	A little Difficulty	8532	5.66 (19.40%)
	A lot of Difficulty	2076	1.23 (4.23%)
Mental Health of Father	Excellent or Very Good	30420	22.02 (75.44%)
	Good	8405	5.86 (20.07%)
	Fair or Poor	1995	1.30 (4.47%)
Mental Health of Mother	Excellent or Very Good	29043	20.96 (71.78%)
	Good	9368	6.49 (22.25%)
	Fair or Poor	2409	1.74 (5.95%)
Family Resilience	All or most of time to all 4 items	1899	1.50 (5.15%)
	All or most of time to 2–3 items	3791	2.68 (9.19%)
	All of most of time to all 0–1 items	35130	25.01(85.64%)

*FPL=Federal Poverty level.

Overall, the descriptive characteristics show that about one in seven children were classified as obese, with significant variability across interpersonal factors like bullying exposure and parental mental health, which provides the context for subsequent analyses.

The supplementary S1 Table compares participants who were retained and those who were excluded. Age and sex distributions were similar between the groups. However, differences appeared in BMI category distributions, race/ethnicity, and FPL. Notably, the excluded group had a higher proportion of children with obesity compared to the retained sample. This indicates that exclusion due to missing data may have disproportionately removed children with obesity, potentially leading to conservative estimates of the link between BMI and mental health outcomes.

The analysis in Table 2 demonstrates a significant association between obesity and the occurrence of LDD and LDA among children aged 6–17 years. In the initial model, obese children exhibited 1.60 times higher odds of experiencing LDD compared to those with a normal BMI (OR= 1.60; 95% CI: 1.27-2.02). Furthermore, after adjusting for age, race, sex, and FPL, this association intensified, revealing that obese children had 2.08 times higher odds of experiencing LDD (aOR 2.08; 95%CI: 1.64-2.64). It was also observed that there exists a dose-response relationship, whereby overweight and obese children present progressively greater odds of LDD than their normal weight counterparts.

**Table 2:** Multivariate Logistic Regression for BMI and LDD/LDA.

BMI Category	LDD (Unadjusted OR, 95% CI)	LDD (aOR^1^, 95% CI)	LDA (Unadjusted OR, 95% CI)	LDA (aOR^2^, 95% CI)
Normal (reference)	1.00	1.00	1.00	1.00
Underweight	0.63 (0.46–0.87)	0.91 (0.65–1.27)	0.85 (0.70–1.02)	1.03 (0.85–1.24)
Overweight	1.41 (1.07–1.84)	1.53 (1.18–1.97)	0.92 (0.80–1.07)	1.01 (0.87–1.18)
Obese	1.60 (1.27–2.02)	2.08 (1.64–2.64)	1.20 (1.01–1.43)	1.48 (1.25–1.76)

^1^Adjusted for age, sex, race/ethnicity, and federal poverty level (FPL).

^2^Adjusted for age, sex, and race/ethnicity.

These results demonstrate clearly that higher BMI is associated with increased odds of LDD and LDA after adjustment for demographic factors.

To address temporal alignment between current BMI and mental health status, sensitivity analyses were conducted using current depression and current anxiety diagnoses only (S2 Table). In these models, the direction of association between BMI category and mental health outcomes remained broadly similar to the primary analysis; however, the magnitude of the associations was attenuated, and confidence intervals widened, and the associations were no longer statistically significant. For example, the adjusted odds ratio for obesity and current depression was 1.26 (95% CI: 0.81–1.96), and for current anxiety was 1.15 (95% CI: 0.52–2.56). The loss of statistical significance is likely due to the substantially smaller number of current cases (n = 575 for depression and n = 637 for anxiety), which reduces statistical power. These findings suggest that the primary results are sensitive to the inclusion of lifetime diagnoses but that the overall direction of association remains consistent.

With LDA, obese children had 1.20 times greater odds of LDA than normal weight children in the unadjusted analyses (OR=1.20; 95% CI: 1.01-1.43). After adjustment for covariates, obese children had also significantly greater odds of LDA than their normal weight counterparts, having 1.48 times greater odds of LDA (aOR 1.48; 95% CI: 1.25-1.76).

Stratified analysis (Table 3, 4 and 5) suggested that the association between obesity and current or lifetime diagnosis of depression varied across some interpersonal and family-context strata, particularly bullying frequency and parental mental health. Among children who were never bullied, those with obesity were more than twice as likely to experience LDD compared to their normal weight peers, when adjusted for covariates (aOR 2.12; 95% CI: 1.45–3.11). This association also remained significant for children who were bullied occasionally (aOR 1.65; 95% CI: 1.14–2.39), though among children who were bullied frequently, the confidence interval for the obesity– LDD association crossed the null (aOR 1.35; 95% CI: 0.87–2.08), indicating limited statistical evidence for an association in this subgroup. A similar trend was seen with maternal mental health. Among children whose mothers had excellent or very good mental health, those with obesity had a 28% higher odds of being LDD (aOR 1.28; 95% CI: 1.02–1.62), while in children whose mothers reported poor mental health, the odds increased to 70% higher (aOR 1.70; 95% CI: 1.07–2.70) as compared to normal weight children. The pattern was even more pronounced when fathers mental health was taken under consideration: children with obesity had significantly higher odds of LDD whether their fathers reported excellent (aOR 1.54; 95% CI: 1.17–2.02), good (aOR 2.09; 95% CI: 1.35–3.22), or poor mental health (aOR 2.54; 95% CI: 1.51–4.29).

**Table 3:** Effect of the Interpersonal Factors on the adjusted Odds of LDD among Children 6-17 years with Obesity. Adjusted Odds Ratios (aOR) for LDD by BMI Category, Stratified by Mother’s Mental Health.

Mother’s Mental Health	BMI Category	aOR^1^	95% CI	p-value
Excellent/ Very Good	Underweight	1.04	0.80–1.35	0.766
	Overweight	0.94	0.77–1.14	0.516
	Obese	1.28	1.02–1.62	**0.035**
Good	Underweight	1.00	0.72–1.39	0.993
	Overweight	0.97	0.72–1.29	0.815
	Obese	1.34	0.99–1.82	0.061
Fair/ Poor	Underweight	0.88	0.44–1.74	0.704
	Overweight	1.16	0.75–1.79	0.503
	Obese	1.70	1.07–2.70	**0.025**

^1^Adjusted for child age, sex, race/ethnicity, and federal poverty level (FPL).

**Table 4:** Effect of the Interpersonal Factors on the adjusted Odds of LDD among Children 6-17 years with Obesity. Adjusted Odds Ratios (aOR) for LDD by BMI Category, Stratified by Father’s Mental Health.

Father’s Mental Health	BMI Category	aOR^1^	95% CI	p-value
Excellent/ Very Good	Underweight	0.96	0.62–1.49	0.862
	Overweight	1.36	1.02–1.82	0.037
	Obese	1.54	1.17–2.02	0.002
Good	Underweight	0.82	0.42–1.57	0.544
	Overweight	1.63	0.99–2.68	0.052
	Obese	2.09	1.35–3.22	0.001
Fair/ Poor	Underweight	0.62	0.28–1.35	0.227
	Overweight	1.20	0.61–2.35	0.599
	Obese	2.54	1.51–4.29	0.001

^1^Adjusted for child age, sex, race/ethnicity, and federal poverty level (FPL).

**Table 5:** Effect of the Interpersonal Factors on the adjusted Odds of LDD among Children 6-17 years with Obesity. Adjusted Odds Ratios (aOR) for LDD by BMI Category, Stratified by Bullying Frequency.

Bullying Frequency	BMI Category	aOR^1^	95% CI	p-value
Never	Underweight	1.09	0.59–2.03	0.781
	Overweight	1.07	0.73–1.56	0.745
	Obese	2.12	1.45–3.11	**<0.001**
Occasional	Underweight	0.80	0.53–1.21	0.292
	Overweight	1.77	1.24–2.52	**0.002**
	Obese	1.65	1.14–2.39	**0.008**
Frequent	Underweight	0.69	0.32–1.45	0.324
	Overweight	1.51	0.81–2.81	0.194
	Obese	1.35	0.87–2.08	0.176

^1^Adjusted for child age, sex, race/ethnicity, and federal poverty level (FPL).

**Fig 1:**
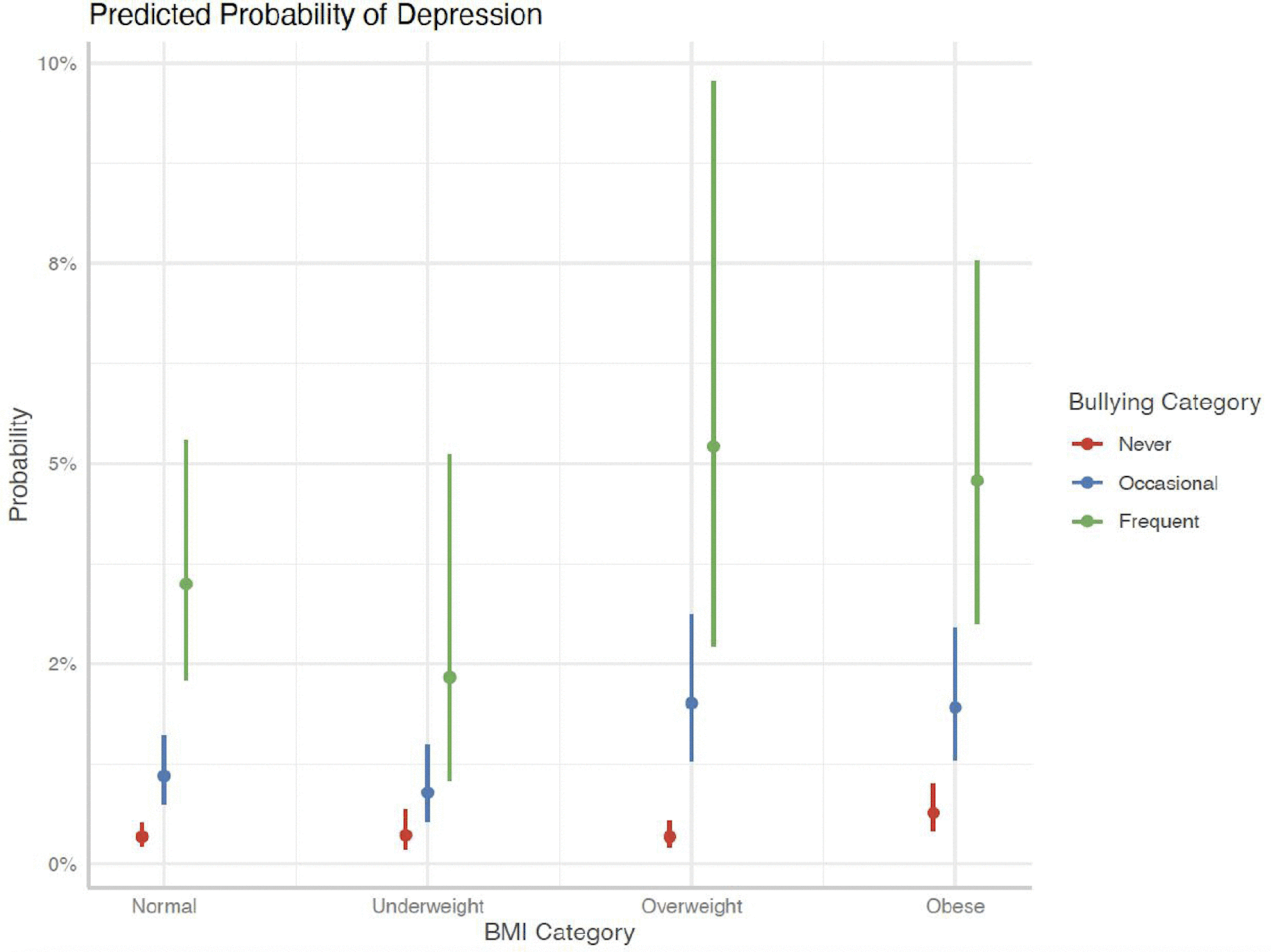
Predicted Probability of LDD - Bullying Category.

**Fig 2:**
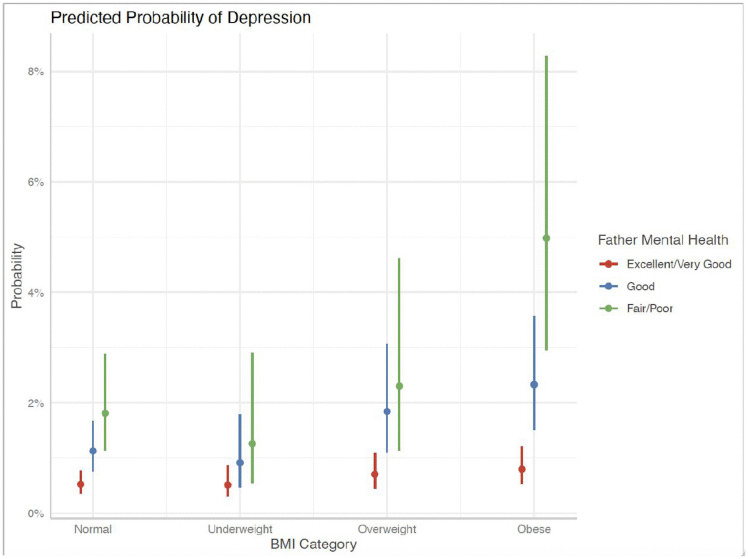
Predicted Probability of LDD- Mental Health of Father.

**Fig 3:**
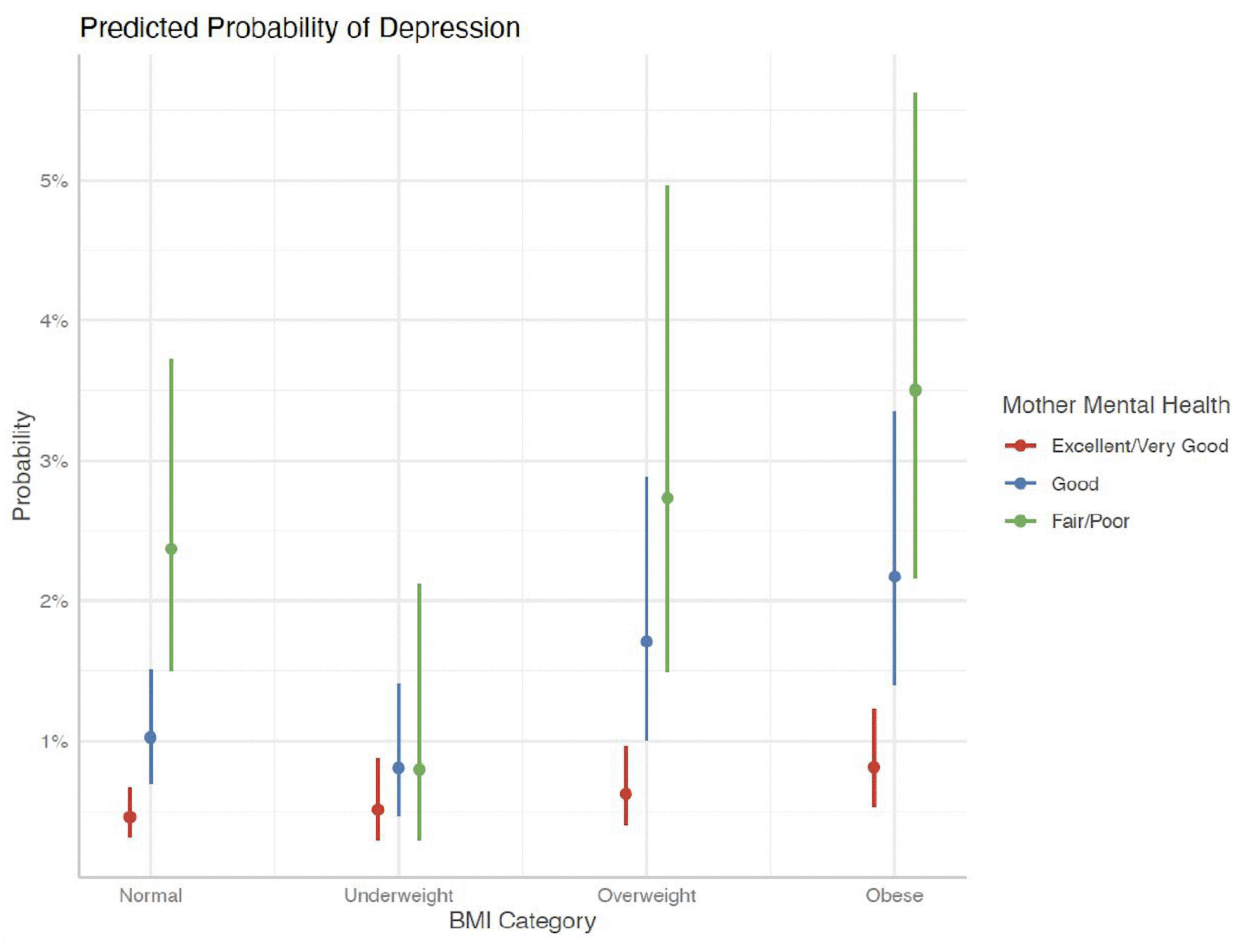
Predicted Probability of LDD- Mental Health of Mother.

Similarly, Figs 1, 2, and 3 presented indicate that children with a history of frequent bullying exhibited significantly greater associated probabilities of LDD across all BMI ranges, particularly among children who were overweight or obese. Furthermore, children whose parents, both mothers and fathers, reported poor mental health demonstrated a significantly increased risk of LDD, with a clear gradient observed from excellent to poor parental mental health. These findings corroborate the interactive roles of interpersonal exposures—namely, bullying and parental psychological well-being—in modifying the association between body weight and mental health outcomes among children.

The supplementary S3 Table demonstrates that the obese + frequent bullying subgroup included n = 399 children, corresponding to a weighted population estimate of 278,751 (0.95% of the analytic sample). This relatively small subgroup size likely reduced precision and may explain the wider confidence interval observed in this stratum. In contrast, larger subgroup sizes were observed in the “never” and “occasional” bullying categories, where associations were estimated with greater precision.

Together, these stratified results suggest that the association between obesity and LDD varies depending on the child’s bullying experiences and parental mental health, highlighting the moderating role of interpersonal environments. Further, these illustrations have higher predicted probabilities of depression among children exposed to frequent bullying across BMI categories, but these visual patterns should be interpreted alongside the wider confidence intervals in stratified regression estimates.

In contrast, **difficulty making friends** and **family resilience** (Table 6) did not interact with obesity, but both were strong correlates of LDD on their own. Children who had a little difficulty making friends had five times higher odds to report LDD (aOR 4.96; 95% CI: 4.07–6.05), and those with a lot of difficulty had almost thirteen times the odds (aOR 12.94; 95% CI: 10.03–16.44), compared to children who reported no difficulty. On the flip side, strong family resilience seemed to offer protection. Children from families that were resilient to “most items” had 43% lower odds of LDD (aOR 0.57; 95% CI: 0.42–0.77), and those from families resilient to “some items” had 60% lower odds of LDD (aOR 0.40; 95% CI: 0.31–0.53) compared to families resilient to all items.

**Table 6:** Adjusted Odds Ratio for LDD by Difficulty in Making Friends and Family Resilience.

Variable	Category	aOR^1^	95% CI
Difficulty Making Friends	No difficulty (reference)	1.00	1.00
	A little difficulty	4.96	4.07–6.05
	A lot of difficulty	12.94	10.03–16.44
Family Resilience	Resilient to all items (reference)	1.00	1.00
	To most items	0.57	0.42–0.77
	To some items	0.40	0.31–0.53

^1^Adjusted for child age, sex, race/ethnicity, and federal poverty level (FPL).

While making friends and family resilience did not interact with BMI, both remained independently associated with LDD, suggesting additional pathways of psychosocial risk and buffering.

When examining the relationship between **body weight and social challenges** related to **LDA in children,** the association of obesity appeared to vary depending on the child’s ability to make friends socially and the mental health of their father. Table 7 shows that among children who reported **no difficulty making friends,** obesity was not significantly linked to LDA when adjusted for age, sex, race/ethnicity and FPL (aOR 1.12; 95% CI: 0.87–1.44). The same pattern held for those with **a little difficulty,** where the odds of LDA were slightly higher for children with obesity, but this was not statistically significant (aOR 1.27; 95% CI: 0.94–1.72). However, among children who had **a lot of difficulty making friends,** the findings were different: children with obesity had twice the odds of experiencing LDA compared to their normal-weight peers (aOR 1.91; 95% CI: 1.13–3.22).

**Table 7:** Effect of the Interpersonal Factors on the LDA among Children 6-17 years with Obesity. Adjusted Odds Ratios (aOR) for LDA by BMI Category, Stratified by Difficulty in Making Friends.

Difficulty in Making Friends	BMI Category	aOR^1^	95% CI	p-value
No difficulty	Underweight	1.05	0.80–1.39	0.724
	Overweight	1.06	0.84–1.33	0.642
	Obese	1.12	0.87–1.44	0.368
Little difficulty	Underweight	0.75	0.52–1.08	0.125
	Overweight	0.79	0.61–1.03	0.081
	Obese	1.27	0.94–1.72	0.121
A lot of difficulty	Underweight	1.34	0.79–2.28	0.277
	Overweight	1.38	0.89–2.14	0.146
	Obese	1.91	1.13–3.22	**0.015**

^1^Adjusted for child age, sex, race/ethnicity, and federal poverty level (FPL).

A similar trend appeared when examining **fathers’ mental health** (Table 8). For children whose fathers reported **excellent or very good mental health,** obesity among obese children, the confidence interval for the obesity– LDA association crossed the null (aOR=1.19; 95% CI: 0.95–1.48), indicating limited statistical evidence for an association in this subgroup, after adjusting for age, sex, race/ethnicity and FPL. Among those whose fathers reported **good** mental health, obesity was associated with a 56% increase in odds of LDA (aOR 1.56; 95% CI: 1.14–2.14). The most substantial effect was observed in children whose fathers had **fair or poor mental health**—here, obesity more than doubled the odds of experiencing LDA (aOR 2.14; 95% CI: 1.34–3.41).

**Table 8:** Effect of the Interpersonal Factors on the LDA among Children 6-17 years with Obesity. Adjusted Odds Ratios (aOR) for LDA by BMI Category, Stratified by Father Mental Health.

Father’s Mental Health	BMI Category	aOR^1^	95% CI	p-value
Excellent/ Very Good	Underweight	1.07	0.85–1.36	0.552
	Overweight	1.00	0.82–1.22	0.983
	Obese	1.19	0.95–1.48	0.126
Good	Underweight	0.92	0.63–1.35	0.666
	Overweight	0.78	0.59–1.04	0.089
	Obese	1.56	1.14–2.14	**0.006**
Fair/ Poor	Underweight	1.21	0.65–2.24	0.549
	Overweight	1.55	0.95–2.54	0.078
	Obese	2.14	1.34–3.41	**0.001**

^1^Adjusted for child age, sex, race/ethnicity, and federal poverty level (FPL).

Thus, for LDA, the association between BMI and internalizing symptoms was strongest among children who had difficulty maintaining friendships and those whose fathers reported poorer mental health*.*

**Fig 4:**
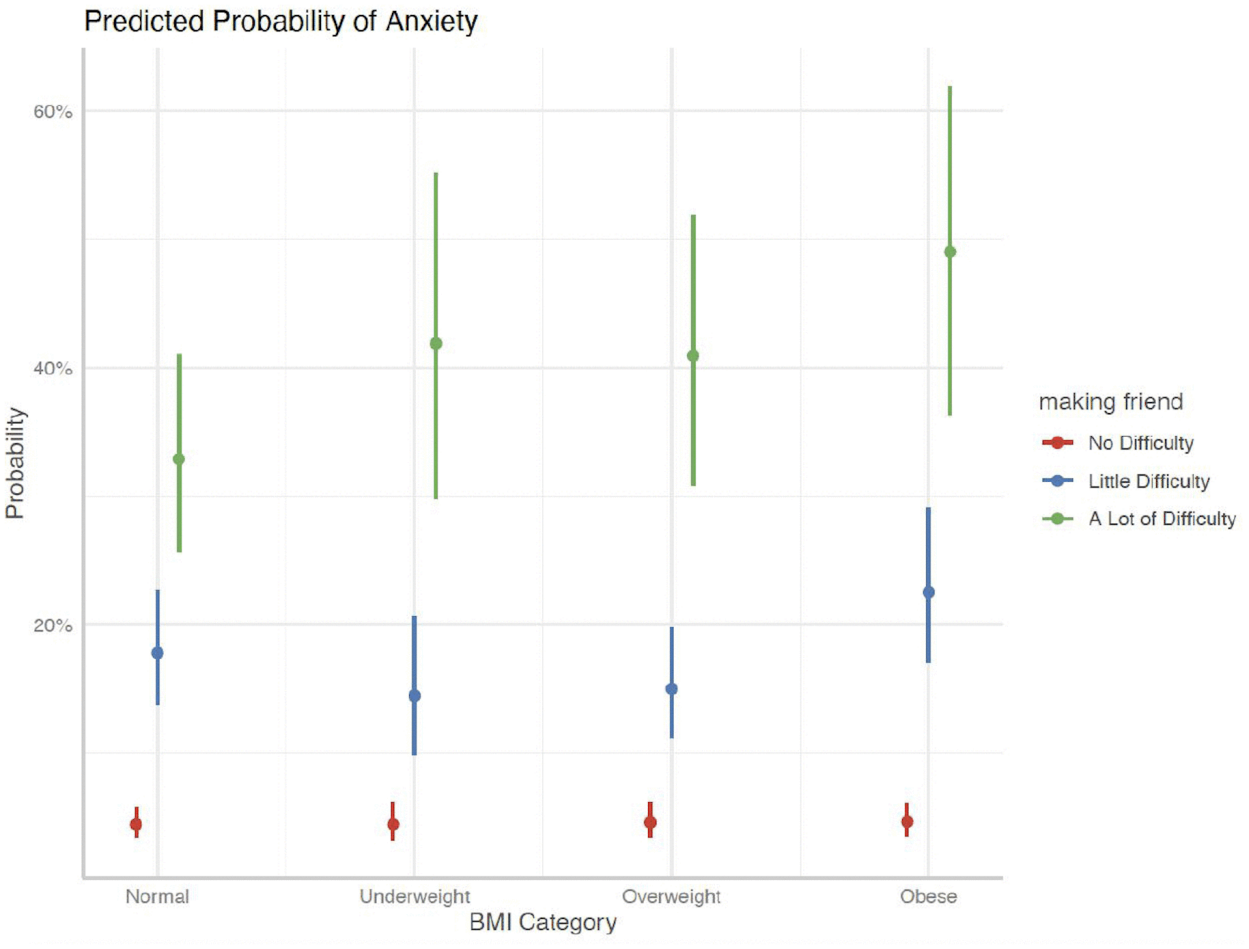
Predicted Probability of LDA– Making Friends.

**Fig 5:**
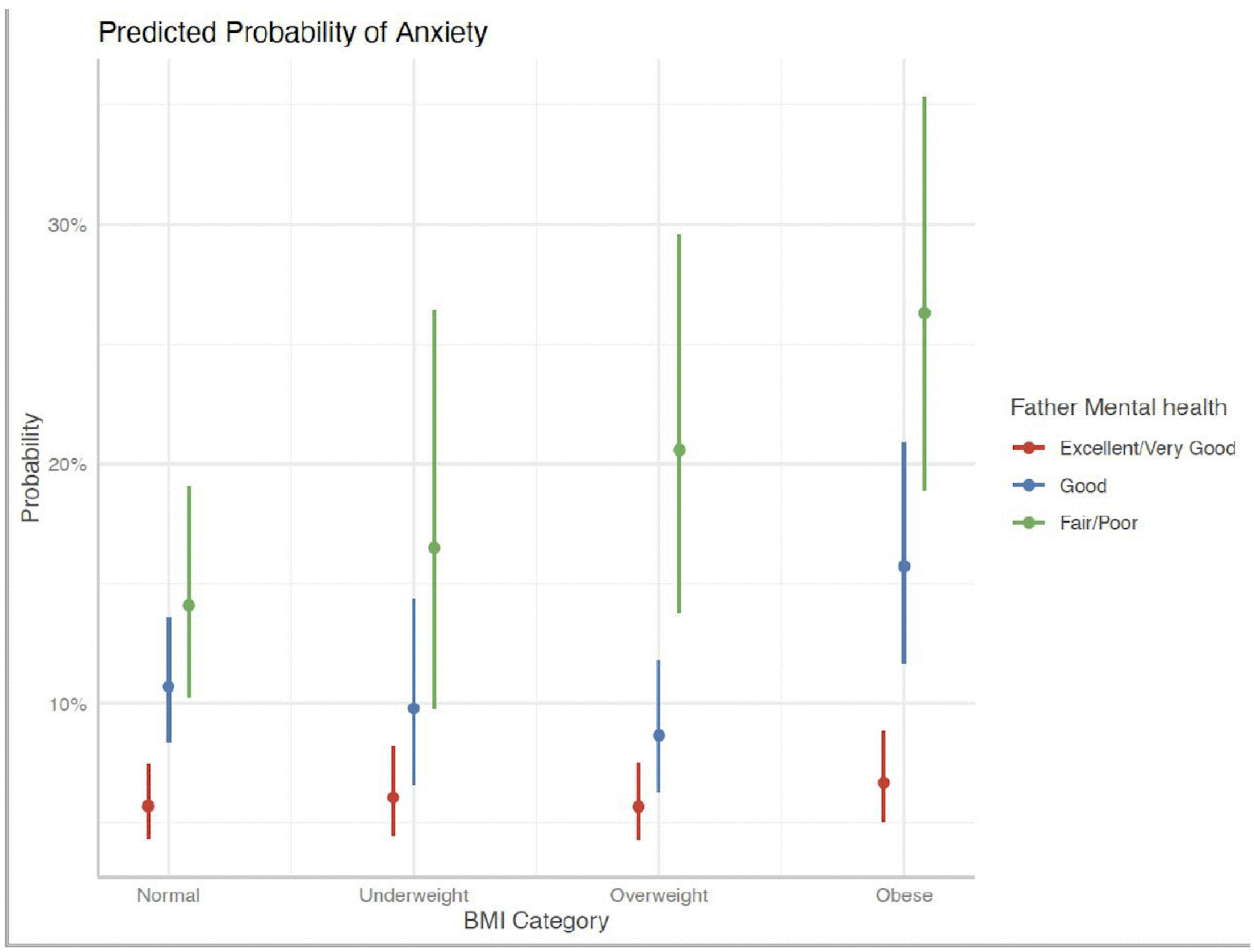
Predicted Probability of LDA– Father’s Mental Health.

Figs 4 and 5 show that the likelihood of LDA was notably higher in children with obesity when their father reported poor or fair mental health, exceeding 25%. Additionally, children who faced more challenges in forming friendships had a significantly higher risk of LDA across all BMI categories, especially among those with obesity. These results indicate that paternal mental health and social integration play a crucial role in modifying the association between BMI and LDA in children.

## Discussion

Our findings are consistent with existing literature on the association between childhood obesity and severe mental health difficulties and support an observed association between obesity and LDD and LDA in childhood populations. It indicates how it is imperative to recognize obesity as an essential risk factor for LDD and LDA in childhood populations [9,53,54]. However, other longitudinal investigations have reported weaker or non-significant associations once baseline emotional symptoms or socioeconomic stressors are rigorously controlled, suggesting that obesity may not uniformly predict mental illness across all contexts [11,12]. A sensitivity analysis restricted to current diagnoses of depression and anxiety showed that the direction of association between obesity and mental health outcomes remained positive, but the magnitude of the associations was attenuated and no longer statistically significant. This attenuation is likely attributable to the substantially smaller number of current cases relative to combined current-or-lifetime diagnoses, resulting in reduced statistical power and wider confidence intervals. The findings suggest that while the association between obesity and mental health is observable in cross-sectional analyses using broader diagnostic definitions, caution is warranted when interpreting the relationship strictly in terms of current mental health status. Longitudinal studies are needed to better establish temporal ordering and causal direction.

This research also adds to the literature by revealing that interpersonal stressors were associated with stronger observed associations in some strata. These risks align with socio-ecological and diathesis-stress models. We observed heterogeneity across BMI groups, particularly between the LDD and LDA pathways. While the literature corroborates this association for both LDD and LDA, our study has identified a nuance: BMI exhibits a stronger association with LDD than with LDA.

Although the literature recognizes bullying as a risk factor for LDD and LDA [55], especially demonstrating a dose–response relationship where more frequent victimization predicts greater internalizing risk [20,22,21], another paradox identified in this study was that infrequent bullying was more predictive of LDD than frequent bullying. This interaction was absent in the development of LDA in children. Interestingly, the link between obesity and LDD was most noticeable among children who faced *occasional* bullying rather than frequent bullying. Since the study is cross-sectional, we can’t explain why this pattern occurs. A notable finding was that the obesity–depression association was statistically significant in the never-bullied and occasionally bullied strata, but not in the frequently bullied stratum. This should not be interpreted as evidence that frequent bullying is protective or that repeated victimization builds resilience. A more plausible explanation is limited statistical precision within the frequent-bullying subgroup, potential reporting heterogeneity, or the possibility that frequently bullied children manifest distress through outcomes not captured by the diagnostic variables examined here. Because the confidence interval in the frequent-bullying subgroup crossed the null, this pattern should be interpreted conservatively as inconclusive rather than substantively different. As demonstrated in the supplementary analysis, this subgroup constituted less than 1% of the analytic sample, resulting in reduced statistical power and wider confidence intervals. Therefore, the observed attenuation of association is more likely attributable to limited sample size rather than a true protective or resilience effect. We can assume that parents have underreported the frequency of bullying, as it engenders stigma or exposes vulnerabilities within the family, particularly when the child conceals the victimization. Nonetheless, this interpretation is speculative and requires testing in future longitudinal studies. Consequently, we believe that our findings suggest the necessity for screening mental health outcomes, even amidst seemingly minor or infrequent peer conflicts. In addition, stratified estimates are sensitive to subgroup size; therefore, we provide the unweighted and weighted counts for the obese + frequent bullying subgroup in the Supplement.

Previous literature posited that maternal and paternal mental health have significant effects upon children’s emotional regulation, attachment style, and behavior development [31,34,35]. A few studies have reported weaker associations after adjusting for socioeconomic stressors, and found that genetic heritability may partially account for these relationships—indicating parental mental health effects are not uniformly environmental [30,33]. The literature supported that this phenomenon was more commonly observed in mother-child relationships than in father-child relationships. The strong link between poor maternal mental health and child LDD may reflect multiple mechanisms, including family stress, caregiving disruption, shared environmental adversity, genetic vulnerability, or differential reporting by the responding parent. Because parental mental health was also part of the same reporting system, these findings should not be interpreted as purely environmental effects. As primary caregivers, mothers often provide most of the emotional, educational, and health-related support. Depressed or anxious mothers may be less responsive, nurturing, or emotionally attuned to their children and therefore contribute to poorer caregiving quality, which then indirectly affects the psychosocial development of children, but this would be outside the scope of our research. In many households, mothers continue to occupy central caregiving roles, which may shape the observed association between maternal mental health and child emotional outcomes; however, this interpretation remains tentative in the absence of direct caregiving measures.

While research has mostly focused on maternal mental health and its impact on child emotional outcomes, our results also reveal a significant link between poor paternal mental health and adverse child mental health outcomes. This may mirror the wider influence of the family’s emotional climate, where paternal psychological distress can impact parenting styles, emotional support, and overall household dynamics. Similar to maternal mental health, paternal well-being might contribute to a shared psychosocial environment that increases children’s emotional vulnerability. Nonetheless, because paternal influences are often less studied in child mental health research, these findings should be viewed with caution and underscore the need for more research into father-specific effects.

Despite other interpersonal stresses, a higher family resilience was associated with lower odds of depression against LDD [56,57]. Studies reported inconsistent or limited moderation effects, showing that resilience does not uniformly protect against LDA or chronic adversity [15,39]. Our findings indicate that, in adjusted models, greater family resilience was associated with lower odds of depression, but we did not observe clear evidence that family resilience modified the association between BMI and anxiety. These patterns may reflect outcome-specific psychosocial processes, but they may also reflect limitations of cross-sectional and single-informant measurement. The distinction may be traced back to the different cognitive and affective mechanisms underpinning these mental health outcomes. LDD is characterized by a negative schema of hopelessness and self-blame, which may improve more with cohesive family processes. In contrast, LDA—associated with hyperarousal and vigilance—may necessitate non-family-centred interventions.

The importance of multi-level approaches that consider interpersonal context is strengthened by our findings. Paediatric screening practices could incorporate routine assessment of peer relationship difficulties and parental mental health where appropriate. School-based anti-bullying programs and caregiver-focused supports may help address some of the interpersonal environments associated with children’s emotional well-being. Parent-focused interventions may be relevant targets for future studies for children’s emotional well-being, while school-based anti-bullying and peer-bonding programs—especially targeting weight-based discrimination—may address key social risk factors. Policy approaches may address the disproportionate burden on low-income and minority families, embedding obesity and mental health services in equity frameworks. At the policy level, the findings suggest that holistic approaches to children’s health may be considered, taking into account both physical and mental aspects. Policymakers may also recognize that the mental health disparities associated with obesity differentially impact low-income and minority families, and therefore are of relevance to an equity framework that informs the process of planning and budgeting programs. While the NSCH dataset does not directly evaluate the effectiveness of interventions, these results highlight potential domains where supportive policies could be relevant.

While providing additional insights into the associations between psychosocial determinants and childhood obesity as well as mental health, several limitations should be acknowledged. This study has several limitations. First, the NSCH is cross-sectional and therefore does not permit conclusions about temporal ordering or causality; reverse causation remains plausible, including the possibility that mental health conditions or their treatment may influence body weight. Differences between retained and excluded participants were observed for BMI category, race/ethnicity, and poverty level, indicating potential selection bias due to the exclusion of participants with missing data. Notably, children with obesity were more likely to be excluded, suggesting that the observed associations may represent conservative estimates rather than inflated associations. Second, all key study variables were reported by the same parent or guardian, including child BMI inputs, mental health diagnoses, bullying exposure, peer difficulties, family resilience, and parental mental health, increasing the likelihood of Type II error This raises the possibility of common method variance and same-informant bias, whereby parental perceptions or mental health status may influence reporting across multiple domains, inflating observed associations. Third, BMI was derived from parent-reported height and weight rather than direct measurement, which may introduce potential misclassification. Fourth, the primary outcomes combined current and lifetime diagnosis status to preserve sample size, which may weaken temporal correspondence with current BMI; therefore, we report sensitivity analyses using current diagnosis outcomes separately. Fifth, some subgroup analyses, particularly those involving frequent bullying, may have been underpowered, leading to imprecise estimates and wider confidence intervals. Sixth, because multiple stratified analyses were conducted, some findings may reflect chance and should be interpreted as exploratory. Finally, these findings are specific to a U.S. survey context and may not generalize to other sociocultural or health-system settings. Future research should utilize longitudinal designs to establish causal directions, involve multiple informants to increase validity, and include qualitative or mixed-method approaches to explain counterintuitive findings such as the lack of a dose–response relationship for infrequent bullying. Future research should confirm the generalizability of these associations in comparative studies across diverse cultural and structural contexts.

In sum, obesity in children is embedded within a network of interpersonal and contextual influences that differentially predict LDD and LDA. These findings support further longitudinal and multi-informant research on how family and peer contexts relate to the co-occurrence of obesity and mental health problems in childhood, and they may help inform screening and intervention priorities across healthcare, education, and family systems.

## Supporting information

S1 Table Characteristics of retained and excluded participants before final analytic sample selection. (DOCX)

S2 Table Sensitivity analysis using current depression and current anxiety outcomes only. (DOCX)

S3 Table Unweighted and weighted cell counts for BMI category by bullying frequency. (DOCX)
