# Supplementary material for: Between the scales and the scars: The interpersonal moderators of the association between Body Mass Index and parent-reported depression/anxiety diagnoses in U.S. children: Evidence from the 2021–22 National Survey of Children’s Health (NSCH)
Source: PLOS Glob Public Health. 2026 May 20;6(5):e0006462. doi: 10.1371/journal.pgph.0006462 (PMC13189307; doi:10.1371/journal.pgph.0006462)
Supplement: S1 Table — (DOCX) [file pgph.0006462.s001.docx]

**S1_Table: Characteristics of retained and excluded participants before final analytic sample selection**

| Variable | Category | Retained n (Weighted %) | Excluded n (Weighted %) | P-value |
| --- | --- | --- | --- | --- |
| **Age group** | 6–11 years | 19,108  (49.39%) | 10,233 (47.79%) | 0.072 |
|  | 12–17 years | 21,712  (50.61%) | 13,482 (52.21%) |  |
| **Sex** | Male | 21,205  (50.89%) | 12,364 (52.19%) | 0.561 |
|  | Female | 19,615  (49.11%) | 11,351 (47.81%) |  |
| **BMI category** | Underweight | 3,613  (9.19%) | 1,639  (8.49%) | <0.001 |
|  | Normal | 25,952  (60.49%) | 11,731 (52.19%) |  |
|  | Overweight | 5,806  (15.09%) | 3,346  (16.59%) |  |
|  | Obese | 5,449  (15.24%) | 4,196  (22.74%) |  |
| Race/ethnicity | Hispanic | 5072  (23.49%) | 4449  (30.89%) | <0.001 |
|  | White-Non Hispanic | 28911  (57.29%) | 12798  (36.89%) |  |
|  | Black Non-Hispanic | 1445  (7.49%) | 2937  (21.29%) |  |
|  | Others/Multi Racial Non Hispanic | 5392  (11.59%) | 3531  (10.89%) |  |
| FPL | 0-99% | 3132  (10.59%) | 5380  (30.09%) | <0.001 |
|  | 100-199% | 5161  (16.19%) | 5484  (25.89%) |  |
|  | 200-399% | 12206 (  31.00%) | 6902  (26.00%) |  |
|  | 400% or greater | 20321  (42.09%) | 5949  (18.19%) |  |
