# Supplementary material for: Between the scales and the scars: The interpersonal moderators of the association between Body Mass Index and parent-reported depression/anxiety diagnoses in U.S. children: Evidence from the 2021–22 National Survey of Children’s Health (NSCH)
Source: PLOS Glob Public Health. 2026 May 20;6(5):e0006462. doi: 10.1371/journal.pgph.0006462 (PMC13189307; doi:10.1371/journal.pgph.0006462)
Supplement: S2 Table — (DOCX) [file pgph.0006462.s002.docx]

**S2_Table: Sensitivity analysis using current depression and current anxiety outcomes only**

| Outcome | BMI Category | aOR¹ | 95% CI | p-value |
| --- | --- | --- | --- | --- |
| Current Depression | Underweight vs Normal | 0.80 | 0.41–1.53 | 0.495 |
| Current Depression | Overweight vs Normal | 1.43 | 0.83–2.48 | 0.201 |
| Current Depression | Obese vs Normal | 1.26 | 0.81–1.96 | 0.313 |
| Current Anxiety | Underweight vs Normal | 0.78 | 0.45–1.36 | 0.386 |
| Current Anxiety | Overweight vs Normal | 0.71 | 0.41–1.25 | 0.240 |
| Current Anxiety | Obese vs Normal | 1.15 | 0.52–2.56 | 0.723 |

¹ aOR= Adjusted for age, sex, race/ethnicity, and federal poverty level (FPL).

**Sample size for sensitivity analysis:**

| Outcome | Cases | Non-cases |
| --- | --- | --- |
| Current Depression | 575 | 40,245 |
| Current Anxiety | 637 | 40,183 |
