# Supplementary material for: Between the scales and the scars: The interpersonal moderators of the association between Body Mass Index and parent-reported depression/anxiety diagnoses in U.S. children: Evidence from the 2021–22 National Survey of Children’s Health (NSCH)
Source: PLOS Glob Public Health. 2026 May 20;6(5):e0006462. doi: 10.1371/journal.pgph.0006462 (PMC13189307; doi:10.1371/journal.pgph.0006462)
Supplement: S3 Table — (DOCX) [file pgph.0006462.s003.docx]

**S3_Table: Unweighted and weighted cell counts for BMI category by bullying frequency**

| BMI Category | Bullying Frequency | Unweighted N | Weighted N | Weighted % of Analytic Sample |
| --- | --- | --- | --- | --- |
| Normal | Never | 15,744 | 11,465,731 | 39.30 |
| Normal | Occasional | 9,205 | 5,642,295 | 19.30 |
| Normal | Frequent | 1,003 | 555,726 | 1.90 |
| Underweight | Never | 2,067 | 1,642,101 | 5.62 |
| Underweight | Occasional | 1,391 | 951,949 | 3.26 |
| Underweight | Frequent | 155 | 76,649 | 0.26 |
| Overweight | Never | 3,381 | 2,855,189 | 9.78 |
| Overweight | Occasional | 2,124 | 1,353,366 | 4.63 |
| Overweight | Frequent | 301 | 193,960 | 0.66 |
| Obese | Never | 2,937 | 2,680,789 | 9.18 |
| Obese | Occasional | 2,113 | 1,504,677 | 5.15 |
| Obese | Frequent | 399 | 278,751 | 0.95 |
