# Supplementary material for: Between the scales and the scars: The interpersonal moderators of the association between Body Mass Index and parent-reported depression/anxiety diagnoses in U.S. children: Evidence from the 2021–22 National Survey of Children’s Health (NSCH)
Source: PLOS Glob Public Health. 2026 May 20;6(5):e0006462. doi: 10.1371/journal.pgph.0006462 (PMC13189307; doi:10.1371/journal.pgph.0006462)
Supplement: Attachment — Submitted filename: RE PGPH-D-25-02696_R1.pdf [file pgph.0006462.s007.pdf]

PLOS Global Public Health

Between the Scales and the Scars: The Interpersonal Burden of Body Weight on Children's Mental Health: Evidence from the 2021- 22 National Survey of Children's Health (NSCH)

--Manuscript Draft--

**Manuscript Number:**	PGPH-D-25-02696R1
**Article Type:**	Research Article
**Full Title:**	Between the Scales and the Scars: The Interpersonal Burden of Body Weight on Children's Mental Health: Evidence from the 2021- 22 National Survey of Children's Health (NSCH)
**Short Title:**	Interpersonal Determinants of the Body-Mind Burden in the U.S. Children
**Corresponding Author:**	Fnu Kajal<br>The University of Arizona Mel and Enid Zuckerman College of Public Health<br>Tucson, UNITED STATES OF AMERICA
**Order of Authors:**	Fnu Kajal
	Bin Suh
	Zhao Chen
	Maia ingram
	Scott Carvajal
	Karl Krupp
	Purnima Madhivanan
**Keywords:**	obesity, mentalhealth, interpersonal, NSCH, bullying
**Abstract:**	Background:<br>Childhood obesity has emerged as a significant public health concern, with an increasing body of evidence correlating it with a heightened risk of lifelong diagnosis of depression and anxiety(LDD/LDA). This study aims to examine the impact of less-explored interpersonal dynamics on the association between obesity and lifetime diagnoses of depression and anxiety in children.<br>Methods: A cross-sectional study of 40,820 U.S. children aged 6-17 used survey-weighted logistic regression on the 2021-22 NSCH data. The study examined the moderating effects of bullying, peer relationships, parental mental health, and family resilience on mental health outcomes (LDD & LDA), utilizing interaction terms categorized by Body Mass Index (BMI), adjusted for age, sex, race/ethnicity, and poverty levels.<br><br>Results:<br>Obesity was significantly associated with higher odds of both depression and anxiety in children aged 6-17 years. Obese children had 2.08 times higher odds of depression (aOR 2.08; 95% CI: 1.64-2.64) and 1.48 times higher odds of anxiety (aOR 1.48; 95% CI: 1.25-1.76) compared to normal-weight peers. Significant moderating effects were observed for depression (adjusted for age, sex, race/ethnicity and poverty levels): never bullied (aOR = 2.12; 95% CI: 1.45-3.11), maternal mental health (aOR = 1.70; 95% CI: 1.70- 2.70), and paternal mental health (aOR = 2.54; 95% CI: 1.51-4.29).<br>For anxiety, moderating effects were seen for making friends (aOR = 1.91; 95% CI: 1.13-3.22) and father mental health (aOR = 2.14; 95% CI: 1.34-3.41).<br>Conclusions:<br>Obesity is associated with interpersonal stressors, which in turn, significantly escalate the risk of the development of depression and anxiety in children. Public health interventions must focus on addressing modifiable relational environments, including bullying and familial relationships, to disrupt the pathways connecting obesity with mental illness in children.
**Additional Information:**	

Powered by Editorial Manager® and ProduXion Manager® from Aries Systems Corporation

Question	Response
**Financial Disclosure**<br><br>Enter a financial disclosure statement that describes the sources of funding for the work included in this submission and the role the funder(s) played. This includes grants and any commercial funding of the work or authors.<br><br>This statement will be typeset if the manuscript is accepted for publication.<br><br>*Please review the submission guidelines and the instructions link below for detailed requirements and guidance.*	The author(s) received no specific funding for this work."
**Competing Interests**<br><br>On behalf of all authors, disclose any competing interests that could be perceived to bias this work.<br><br>This statement will be typeset if the manuscript is accepted for publication.<br><br>*Please review the instructions link below and PLOS Global Public Health's competing interests policy to determine what information must be disclosed at submission.*	NA
**Data Availability**<br><br>Before publication, Authors are required to make fully available and without restriction all data underlying their findings. Please see our PLOS Data Policy page for detailed information on this policy.<br><br>A **Data Availability Statement**, detailing where the data can be accessed, is	All data and related meta-data underlying reported findings are available in public data repository (https://nschdata.org/browse/survey/results?q=10511&r=1)

required at first submission. Insert your Data Availability Statement in the box below.<br><br>Please see the data reporting section of our submission guidelines for instructions on what you need to include in your Data Availability Statement.<br><br>This statement will be typeset if the manuscript is accepted for publication.<br><br>*PLOS allows rare exemptions to address legal and ethical concerns. If you have legal or ethical restrictions, please use the box below to detail these in full sentences for the Journal team to consider.*	

Powered by Editorial Manager® and ProduXion Manager® from Aries Systems Corporation

Manuscript
Click here to access/download;Manuscript;Final Manuscript_PLOS.docx

Between the Scales and the Scars: The Interpersonal Burden of Body Weight on Children's Mental Health: Evidence from the 2021- 22 National Survey of Children's Health (NSCH)

Fnu Kajal[1], Bin Suh[2], Chen Zhao[3], Maia Ingram[4], , Scott Carvajal[5], Karl Krupp[6], Purnima Madhivanan[7]

Corresponding Author's Details:

[1]Fnu Kajal, M.D., M.Sc., DrPH Candidate, Mel & Enid Zuckerman College of Public Health, University of Arizona.
Email: kkajal@arizona.edu
ORCiD: https://orcid.org/0000-0002-7971-0237

[2]Bin Suh, Senior Epidemiologist, Arizona Department of Health Services.
Email: bin.suh@azdhs.gov
ORCiD: https://orcid.org/0000-0001-8853-4709

[3]Zhao Chen, PhD, MPH, Distinguished Professor, Epidemiology and Biostatistics, Mel & Enid Zuckerman College of Public Health, University of Arizona.
Email: zchen@arizona.edu
ORCiD: https://orcid.org/0000-0002-5659-0447

[4]Maia Ingram, MPH, Director of Participatory Research and Practice and Co-Director, Arizona Prevention Research Center, Mel & Enid Zuckerman College of Public Health, University of Arizona.
Email: maiai@arizona.edu
ORCiD: https://orcid.org/0000-0002-6517-7697

[5]Scott Carvajal, PhD, Departmental Chair and Professor, Health Promotion Sciences, Mel & Enid Zuckerman College of Public Health, University of Arizona..
Email: carvajal@arizona.edu
ORCiD: https://orcid.org/0009-0003-2106-5305

[6]Karl Krupp, MSc, PhD, Assistant Professor, Division of Public Health Practice & Translational Research, Mel & Enid Zuckerman College of Public Health, University of Arizona.
Email: kkrupp@arizona.edu
ORCiD: https://orcid.org/0000-0001-7001-9194

[7]Purnima Madhivanan, MBBS, MPH, PhD, Associate Professor, Health Promotion Sciences, Mel & Enid Zuckerman College of Public Health, University of Arizona.
Email: pmadhivanan@arizona.edu
ORCiD: https://orcid.org/0000-0001-7818-3394

Abstract

Background:

Childhood mental health issues have emerged as a significant public health concern, with an increasing body of evidence linking them to a higher risk of children's obesity. This study aims to examine the impact of less-explored interpersonal dynamics on the association between obesity and LDA/LDD in children.

Methods:

A cross-sectional study of 40,820 U.S. children aged 6-17 used survey-weighted logistic regression on the 2021-22 NSCH data. The study examined the moderating effects of bullying, peer relationships, parental mental health, and family resilience on mental health outcomes (LDD & LDA), utilizing interaction terms categorized by Body Mass Index (BMI), adjusted for age, sex, race/ethnicity, and poverty levels.

Results:

Obesity was significantly associated with higher odds of both **depression** and **anxiety** in children aged 6-17 years. Obese children had **2.08 times higher odds** of depression (aOR 2.08; 95% CI: 1.64-2.64) and **1.48 times higher odds** of anxiety (aOR 1.48; 95% CI: 1.25-1.76) compared to normal-weight peers. Significant moderating effects were observed for **depression (adjusted for age, sex, race/ethnicity and poverty levels)**: never bullied (aOR = 2.12; 95% CI: 1.45-3.11), maternal mental health (aOR = 1.70; 95% CI: 1.70- 2.70), and paternal mental health (aOR = 2.54; 95% CI: 1.51-4.29). For **anxiety**, moderating effects were seen for **making friends** (aOR = 1.91; 95% CI: 1.13-3.22) and **father mental health** (aOR = 2.14; 95% CI: 1.34-3.41).

Conclusions:

Obesity is associated with interpersonal stressors, especially bullying and adverse familial environments, which, in turn, significantly escalate the risk of the development of depression and anxiety in children. Public health interventions must focus on addressing modifiable relational environments, including bullying and familial relationships, to help disrupt the pathways through which obesity contributes to mental illness in children.

Comment [H1]: You were asked to add recommendation(s) the other time, but you did not. How do you contribute to the knowledge when there is no recommendation(s)

Introduction:

Childhood obesity has emerged as a pressing public health concern with significant implications for both physical and mental well-being. On a global scale, childhood obesity has proliferated at an unprecedented rate in recent decades. According to the World Health Organization, it was estimated in 2022 that more than 390 million children aged 5 to 19 were classified as overweight, and 160 million were obese (1). In the United States, nearly 20 percent of children experience obesity, and the rates of childhood obesity have increased fourfold since 1990 (2), forming what is increasingly recognized as a "Body–Mind Burden"(3,4). Concomitantly, the incidence of mental health issues in children has spiked, with nearly 50% of adolescents experiencing at least one mental illness, and emergency visits for mental health issues rising by 37% in recent years (5,6).

This epidemiological convergence is not coincidental. There is increasing evidence for a bidirectional relationship between obesity and mental health. Children with excess weight are more likely to experience depressive symptoms, anxiety, and social withdrawal, as shown across multiple meta-analyses (7–10). The reverse also holds: adolescents with depression have a 70% increased risk of obesity, particularly

among females (11,12).. Luppino et al. established this reciprocal risk in a seminal meta-analysis, finding that obesity heightens depression risk, and vice versa (13). Although biological and behavioural determinants (e.g., dietary intake and physical inactivity) play a role in these associations, they do not fully account for the emotional distress presented by children with obesity. Instead, the pathways seem to be socially mediated. Teasing/Bullying, stigma, parental mental health, family routines, and peer dynamics are increasingly implicated as effect modifiers in this relationship (14–16). Yet, few studies have comprehensively modelled these interpersonal variables.

Among these, bullying emerges as an exceptionally robust interpersonal risk factor. Children with obesity have up to 46% higher odds of being bullied, compared to their healthy-weight peers (17).

According to the standards established by the World Health Organization (WHO), obesity is defined as a measurement that surpasses two standard deviations above the WHO growth reference median (World Health Organization [WHO], 2024). The Centers for Disease Control and Prevention (CDC) defines childhood obesity as having a body mass index (BMI) that is at or exceeds the 95th percentile for age and sex (CDC US Government, 2024).

Victimization, especially related to weight, can exacerbate internalized stigma and trigger depressive and anxious symptomatology (18,19). Longitudinal studies show that the mental health impact of obesity is significantly magnified among children who are bullied (20,21). Additionally, children classified as bully-victims experience the

highest rates of depression, suicidal ideation, and behavioural problems (22,23). Although bullying is widely acknowledged as linked to obesity and mental health issues, few studies have analyzed bullying together with other interpersonal and family factors within a single framework, especially using nationally representative data.

Peer relationships more broadly also serve as salient moderators. Quality friendships and strong peer attachment buffer children against the psychological impact of social exclusion due to obesity (24,25). Increasing evidence indicates that secure peer attachment is protective for emotional health and well-being (26), whilst peer rejection evokes distress both within and outside the context of overt bullying (27,28).

In the familial sphere, parental mental health—particularly maternal depression—has consistently been linked to both child obesity and mental health issues. Maternal depression influences parenting styles, feeding practices, emotional availability, and home routines, all of which mediate the pathway from obesity to poor emotional outcomes (29–31). The risk is heightened with chronic depressive symptoms, as shown in cohort studies and meta-analyses (32,33). However, paternal mental health remains underexplored, and few studies have disaggregated effects by caregiver role or gender (34,35). The impact of paternal mental health remains relatively understudied**,** with limited analyses distinguishing effects across different caregiver roles. Emerging evidence indicates that fathers' psychological well-being may have unique influences on a child's emotional development.

Family resilience—defined as the ability of families to adapt positively to adversity—has also emerged as a protective factor. Findings from studies that used the NSCH data have shown that family resilience can attenuate the deleterious effects of

bullying and adversity on internalizing symptoms (36,37). For example, a study using data from the COVID-19 pandemic found that family functioning was positively related to adolescent anxiety and depression(38,39). Yet, its role in buffering the psychological consequences of obesity has been underexplored, with few studies testing interaction effects in nationally representative datasets (40,41).

Overall, previous research indicates that obesity might interact with the social environment to influence children’s emotional health, but few studies have simultaneously explored bullying, peer relations, parental mental health, and family resilience as factors moderating this link. To fill this gap, this study utilizes nationally representative NSCH 2021–22 data and a socio-ecological approach to examine how these interpersonal elements impact the relationship between BMI and LDD/LDA diagnoses in children aged 6–17.

Theoretical Framework:

Mental health conditions in children are multifactorial, necessitating multidimensional theoretical frameworks. This research is grounded in two complementary frameworks—the socio-ecological model and the diathesis-stress model—which explain how individual predispositions intersect with interpersonal and contextual stressors to affect mental health.

The **SEM**, originally proposed by Bronfenbrenner and expanded by McLeroy et al. into five levels of influence—Intrapersonal, Interpersonal, Institutional, Community, and Public Policy—underscores the dynamic interrelations between individuals and their environments (42,43). While intrapersonal factors like sleep and activity patterns are essential, interpersonal stressors such as bullying, parental mental

health, peer isolation, and family resilience are increasingly recognized as critical modifiers of mental health outcomes in children with obesity.

The diathesis-stress model dovetails with this ecological lens and explains how genetic and physiological predispositions intersect with environmental adversities to create mental illness (44,45). We view obesity in this framework as a biological condition and psychosocial stressor that is experienced with stigma and social rejection. Nonetheless, these adversities may be ameliorated by protective interpersonal factors (e.g., emotionally balanced parenting and positive peer support). In tandem, such models provide a comprehensive framework for understanding how relational life experiences impact the emotional consequences of childhood obesity.

METHODOLOGY

Data Source

The data for this study were obtained from the Child and Adolescent Health Measurement Initiative (CAHMI)/NSCH for the 2021-2022 period, which is publicly available (48). The NSCH, sponsored by the U.S. Department of Health and Human Services, is conducted by the Census Bureau and has been providing nationally representative data on children's health since 2002-2003. The survey uses instruments whose reliability and validity have been supported in numerous studies.

Study Design

This study is a secondary data analysis of a cross-sectional survey examining the association between obesity and mental health issues, particularly depression and

anxiety. It focuses on identifying key interpersonal determinants that modify the association between obesity and mental health issues in children.

Study Population

The study population consists of children and adolescents aged 6 to 17, as reported in the 2021-2022 NSCH survey (49). This diverse and nationally representative sample highlights a critical developmental period where obesity and mental health intersect, making it an ideal setting for investigating these issues and ensuring credible, actionable data to support research objectives.

The current research represented a sample size of 40,820 children, providing nationally representative data on their health and well-being. Data collection occurred between June 2021 and January 2022. The participants were the parents and guardians of children. The data were collected via the web (90%) and mail (10%), with an approximate response rate of 65%.

Data Collection Tools/Measures: All measures were parent-reported (49). This may introduce the potential for reporting bias, such as misclassification of BMI. However, many previous studies on NSCH data have indicated the acceptable validity of parent-reported measures (50,51)

Outcomes (Mental Health Measures): The outcome variables were ‘depression’ and ‘anxiety’ as assessed using the Revised Child Anxiety and Depression Scale (52). Regarding depression and anxiety, responses encompass the current diagnosis and historical data for children aged 6-17. The responses related to depression and anxiety were classified as binary responses for the purpose of analysis. Given the limited sample size for current diagnoses, we combined current and lifetime indicators to preserve statistical power; however, this approach may

obscure temporal differences. Lifetime diagnosis of depression (LDD)/ Lifetime Diagnosis of Anxiety (LDA) refers to children who have ever been diagnosed with depression/anxiety, including those who are currently depressed, and those who were previously diagnosed but no longer meet the criteria. We combined current depression/anxiety, and those who had ever been diagnosed as one category, and those who are not currently experiencing depression or anxiety, for this comparison.

Explanatory Variable (BMI): Obesity was measured using BMI based on parent-reported height and weight, categorized according to CDC guidelines (CDC, 2016) into four groups by age-adjusted percentile: Underweight (less than 5th percentile), Normal weight (5th to 84th percentile), Overweight (85th to 94th percentile), and Obese (95th percentile or above)

Covariates: Covariates included the child's age, sex, race/ethnicity, and federal poverty levels (FPL), which represent the socioeconomic status of the family. The question on the FPL is: "What is the income level (federal poverty level, FPL) of the household that this child lives in? The responses are divided into 0-99% FPL; 100-199% FPL; 200-399% FPL; 400% FPL or greater.

Moderators: These include interpersonal factors that reflect social relationships and peer interactions.

Bullying was assessed by asking respondents to report how often the child experienced bullying in the past 12 months. For the purpose of analysis, the responses were categorized into three categories: levels: "Never in past 12 months"= Never; "1-2 times in past 12 months" and "1-2 times per month," into Occasional and "1-2 times per week" and "Almost every day" into Frequent. This single-item bullying measure has been used in all NSCH cycles and aligns with CDC

Youth Risk Behavior Surveillance (YRBS) definitions of bullying. Prior NSCH documentation confirms the content validity and reliability of this item for population monitoring.

Making or keeping friends involved respondents, indicating the child's level of difficulty in forming and maintaining friendships. The responses provided for this evaluation included "No difficulty," "A little difficulty," and "A lot of difficulty." This indicator originates from the Strengths and Difficulties Questionnaire (SDQ) peer problems subscale, which has demonstrated construct validity and good internal consistency ($\alpha \approx 0.70$) in U.S. samples.

Father's/Mother’s mental health was evaluated by asking respondents to assess the mental health status of the father/mother, if he/she was the primary caregiver. The assessment options included "Excellent/very good", "Good", and "Fair/poor." This global self-rated item has been validated against standardized screening tools, such as the PHQ-2 and K6, showing a moderate to strong correlation in U.S. population surveys.

Family resilience was evaluated by respondents who assessed the family's ability to cope during difficult times. The question asked was: Does this child live in a home where the family demonstrates qualities of resilience during difficult times? The options included “All or most of the time to 0-1 items”, “All or most of the time to 2-3 items”, “All or most of the time to all 4 items.” These items comprise the NSCH Family Resilience Scale, which demonstrates strong internal consistency (Cronbach’s $\alpha = 0.80$) and established construct validity.

All moderator items are part of the validated NSCH item bank maintained by the Child and Adolescent Health Measurement Initiative (CAHMI). Psychometric

properties of these scales have been described in prior technical documentation and peer-reviewed analyses (53,54).

A non-response bias analysis by NSCH showed no signs of bias. We applied weights, stratification, and accounted for clustering to enhance generalizability to the U.S. population of children and provided an unbiased test of statistical significance. The analytic sample included only children with complete responses on BMI, depression, and anxiety questions. Children under 6 years were excluded to avoid sensitivities related to BMI calculations and varying developmental stages. Of the original 64,535 children aged 6–17 years, we excluded 23,715 cases with non-analytic codes (95 were less than 2 or 3 or 5 years old; 99 were missing or refused) for essential study variables. The ultimate analytic sample was comprised of 40,820 children who had valid information on all variables of interest. We compared the excluded and retained participants in terms of age, sex, race/ethnicity, and family income (Table S1); the distributions were broadly comparable. An inspection of the missing data pattern revealed no residual missingness and no need for multiple imputation.

IRB and Ethics Statement:

These survey results are openly available and de-identified; therefore, secondary analysis of the results is, according to federal guidelines, exempt from consideration by an Institutional Review Board (IRB).

Analysis:

The analysis used the R program and survey-weighted statistical procedures to address the NSCH’s complex sampling design, correcting for oversampling, non-

response, and stratification issues. The NSCH data included weights that adjust for unequal selection probabilities, non-response, and post-stratification to accurately represent the population, as per the NSCH survey codebook (49).

The descriptive analysis includes both unweighted and weighted frequencies and percentages across demographic subgroups. A bivariate analysis examined relationships between BMI categories and LDD and LDA, as well as interpersonal variables. Design-based chi-square tests identified statistically significant associations. LDD or LDA were analyzed across demographic and interpersonal variable categories, as well as BMI categories, for statistical associations. Survey-weighted logistic regression models were used to evaluate the association between BMI and LDD & LDA, controlling for covariates and testing interactions with interpersonal variables.. Adjusted odds ratios (aOR) and 95% confidence intervals (95%CI) are reported, with statistical significance set at $p < 0.05$. Survey design variables, including weights, strata, and primary sampling units, were incorporated using the svyglm() function in R, with models defined using a quasibinomial family to address overdispersion in binary outcomes. Given mild overdispersion in the binary outcomes, we used quasibinomial logistic models to obtain robust variance estimates. Multicollinearity was assessed using variance inflation factors (VIF < 2 for all variables). All moderator–BMI interactions were pre-specified based on the socio-ecological framework. Stratified interaction analyses were conducted as exploratory and hypothesis-generating. We did not apply formal corrections for multiple comparisons; therefore, these results should be interpreted with caution regarding type I error risk.

RESULTS

In this study, the sample (Table 1), represented approximately 29 million children in the United States. The sample was evenly distributed among children aged 6-11 (49.3%), and those aged 12-17 years (50.7%). Similarly, the sample was balanced between male (50.9%) and female (49.0%) participants. While 57.3% were non-Hispanic White, 7.5% were non-Hispanic Black, and 11.6% reported other/multiracial. Furthermore, it was found that 60.5% of the children had a normal BMI, while 15.1% were classified as overweight, and 15.3% obese. An LDA was reported in 13.3% of the participants, whereas an LDD occurred in 4.5%. In terms of interpersonal variables, 63.8% of children reported never being bullied within a year. For kids who were bullied, 26.6% were bullied once or twice within the past year, with 5.7% bullied once or twice per month, and lower percentages bullied several times (2.7% per week, 1.1% per day). Additionally, 4.2% of the children indicated difficulties in making friends. It is also noteworthy that 4.5% and 6.0% of children’s parental guardians (father/male and mother/female, respectively) exhibited poor mental health status. Lastly, only 5.2% of families demonstrated characteristics of full resilience across all four assessed items.

Table 1: Descriptive Stats (n=40820) of children 6-17 years in the 2021-2022 NSCH Survey:

Variable	Categories	Unweighted frequency	Weighted frequency (in millions) (Weighted %)
**Age**	6-11 years	19108	14.4 (49.3%)
	12-17 years	21712	14.8 (50.7%)
**Sex**	Male	21205	14.9 (50.9%)
	Female	19615	14.3 (49.1%)
**Race/Ethnicity**	Hispanic	5072	6.9 (23.5%)
	White, Non-Hispanic	28911	16.7 (57.3%)
	Black, Non-Hispanic	1445	2.2 (7.5%)

	Other/Multiracial, non-Hispanic	5392	3.4 (11.6%)
FPL*	0-99%	3132	3.1 (10.6%)
	100-199%	5161	4.7 (16.2%)
	200-399%	12206	9.0 (31.0%)
	400% or greater	20321	12.3 (42.1%)
BMI	UW	3613	2.7 (9.1%)
	Normal	25952	17.7 (60.5%)
	Overweight	5806	4.4 (15.1%)
	Obese	5449	4.5 (15.3%)
Anxiety	No anxiety	34173	25.3 (86.7%)
	LDA	6647	3.9 (13.3%)
Depression	No Depression	38434	27.9 (95.5%)
	LDD	2386	1.3 (4.5%)
**INTERPERSONAL FACTORS**			
Bullying	Never in past 12 months	24129	18.6 (63.8%)
	1-2 times in past 12 months	11959	7.8 (26.6%)
	1-2 times per month	2874	1.7 (5.7%)
	1-2 times per week	1281	0.8 (2.7%)
	Almost everyday	577	0.3 (1.1%)
Making or Keeping friends	No difficulty	30212	22.3 (76.4%)
	A little Difficulty	8532	5.7 (19.4%)
	A lot of Difficulty	2076	1.2 (4.2%)
Mental Health of Father	Excellent or Very Gooc	30420	22.0 (75.4%)
	Good	8405	5.9 (20.1%)
	Fair or Poor	1995	1.3 (4.5%)
Mental Health of Mother	Excellent or Very Gooc	29043	21.0 (71.8%)
	Good	9368	6.5 (22.2%)
	Fair or Poor	2409	1.7 (6.0%)
Family Resilience	All or most of time to all 4 items	1899	1.5 (5.2%)
	All or most of time to 2-3 items	3791	2.7 (9.2%)
	All of most of time to all 0-1 items	35130	25.0 (85.6%)

*FPL=Federal Poverty level

Overall, the descriptive characteristics show that about one in seven children were classified as obese, with significant variability across interpersonal factors like

bullying exposure and parental mental health, which provides the context for subsequent analyses.

Table S1 – Comparison of Sample Characteristics Before and After Exclusions

VARIABLE	CATEGORY	BEFORE EXCLUSION (N≈ 64,535)	AFTER EXCLUSION (N ≈ 40,820)
AGE (YEARS)	Mean ± SD	11.87 ± 3.54	11.78 ± 3.55
SEX	Male	33,569 (51.9%)	21,205 (52.0%)
	Female	30,966 (48.1%)	19,615 (48.0%)
RACE / ETHNICITY	White	9,521 (14.8%)	5,072 (12.4%)
	Hispanic	41,709 (64.6%)	28,911 (70.9%)
	Black	4,382 (6.8%)	1,445 (3.5%)
	Other / Multiracial	8,923 (13.8%)	5,392 (13.2%)
FEDERAL POVERTY LEVEL (FPL)	< 100%	8,512 (13.2%)	3,132 (7.7%)
	100–199%	10,645 (16.5%)	5,161 (12.6%)
	200–399%	19,108 (29.6%)	12,206 (29.9%)
	≥ 400%	26,270 (40.7%)	20,321 (49.8%)

The above supplementary table (Table S1) demonstrates that there were no significant demographic differences between participants who were included and those who were excluded.

The analysis in Table 2 demonstrates a significant association between obesity and the occurrence of LDD and LDA among children aged 6 to 17 years. In the initial model, obese children exhibited 1.60 times higher odds of experiencing LDD compared to those with a normal BMI (OR= 1.60; 95% CI: 1.27-2.02). Furthermore, after adjusting for age, race, sex, and FPL, this association intensified, revealing that obese children had 2.08 times higher odds of experiencing depression (aOR 2.08; 95%CI: 1.64-2.64). It was also observed that there exists a dose-response relationship, whereby overweight and obese children present progressively greater odds of depression than their normal weight counterparts.

Table 2: Multivariate Logistic Regression for BMI and LDD/LDA.

BMI Category	LDD (Unadjusted OR, 95% CI)	LDD (Adjusted[1] OR, 95% CI)	LDA (Unadjusted OR, 95% CI)	LDA (Adjusted[2] OR, 95% CI)
**Normal (reference)**	1.00	1.00	1.00	1.00
**Underweight**	0.63 (0.46–0.87)	0.91 (0.65–1.27)	0.85 (0.70–1.02)	1.03 (0.85–1.24)
**Overweight**	1.41 (1.07–1.84)	1.53 (1.18–1.97)	0.92 (0.80–1.07)	1.01 (0.87–1.18)
**Obese**	1.60 (1.27–2.02)	2.08 (1.64–2.64)	1.20 (1.01–1.43)	1.48 (1.25–1.76)

[1] Adjusted for age, sex, race/ethnicity, and federal poverty level (FPL).
[2] Adjusted for age, sex, and race/ethnicity.

These results demonstrate clearly that higher BMI is associated with increased odds of LDD and LDA after adjustment for demographic factors.

With anxiety, obese children had 1.20 times greater odds of LDA than normal weight children in the unadjusted analyses (OR=1.20; 95% CI: 1.01-1.43). After adjustment for age, sex and race/ethnicity, obese children had also significantly greater odds of LDA than their normal weight counterparts, having 1.48 times greater odds of anxiety (aOR 1.48; 95% CI: 1.25-1.76).

Stratified analysis (Table 3.1, 3.2 and 3.3) showed that the link between obesity and depression shifts depended on key social factors, especially bullying and the mental health of parents. Among children who were never bullied, those with obesity were more than twice as likely to experience LDD compared to their normal weight peers, when adjusted for age, sex, race/ethnicity and FPL (aOR 2.12; 95% CI: 1.45–3.11). This association also remained significant for children who were bullied occasionally (aOR 1.65; 95% CI: 1.14–2.39), though among children who were bullied frequently, the confidence interval for the obesity–depression association crossed the null (aOR 1.35; 95% CI: 0.87–2.08), indicating limited statistical evidence for an association in this subgroup. A similar trend was seen when looking at maternal mental health. Among children whose mothers had excellent or very good mental health, those with obesity had a 28% higher odds of being LDD (aOR 1.28; 95% CI: 1.02–1.62), while in children whose mothers reported poor mental health, the odds jumped to 70% higher (aOR 1.70; 95% CI: 1.07–2.70) as compared to normal weight children. The pattern was even more pronounced when looking at fathers: children with obesity had significantly higher odds of LDD whether their fathers reported excellent (aOR 1.54; 95% CI: 1.17–2.02), good (aOR 2.09; 95% CI: 1.35–3.22), or poor mental health (aOR 2.54; 95% CI: 1.51–4.29).

Similarly, Figures 1, 2, and 3 presented indicate that children with a history of frequent bullying exhibited significantly greater predicted probabilities of depression across all BMI ranges, particularly among children who were overweight or obese. Furthermore, children whose parents, both mothers and fathers, reported poor mental health demonstrated a significantly increased risk of depression, with a clear gradient observed from excellent to poor parental mental health. These findings corroborate the interactive roles of interpersonal exposures—namely, bullying and

parental psychological well-being—in modifying the association between body weight and mental health outcomes among children.

Table 3: Effect of the Interpersonal Factors on the adjusted Odds of LDD among Children 6-17 years with Obesity

Table 3.1: Adjusted Odds Ratios (aOR) for LDD by BMI Category, Stratified by Mother's Mental Health

Mother's Mental Health	BMI Category	aOR¹	95% CI	p-value
**Excellent / Very Good**	Underweight	1.04	0.80–1.35	0.766
	Overweight	0.94	0.77–1.14	0.516
	Obese	1.28	1.02–1.62	**0.035**
**Good**	Underweight	1.00	0.72–1.39	0.993
	Overweight	0.97	0.72–1.29	0.815
	Obese	1.34	0.99–1.82	0.061
**Fair / Poor**	Underweight	0.88	0.44–1.74	0.704
	Overweight	1.16	0.75–1.79	0.503
	Obese	1.70	1.07–2.70	**0.025**

¹ Adjusted for child age, sex, race/ethnicity, and federal poverty level (FPL).

Table 3.2: Adjusted Odds Ratios (aOR) for LDD by BMI Category, Stratified by Father's Mental Health

Father's Mental Health	BMI Category	aOR¹	95% CI	p-value
Excellent / Very Good	Underweight	0.96	0.62–1.49	0.862
	Overweight	1.36	1.02–1.82	0.037
	Obese	1.54	1.17–2.02	0.002
Good	Underweight	0.82	0.42–1.57	0.544
	Overweight	1.63	0.99–2.68	0.052
	Obese	2.09	1.35–3.22	0.001
Fair / Poor	Underweight	0.62	0.28–1.35	0.227
	Overweight	1.20	0.61–2.35	0.599
	Obese	2.54	1.51–4.29	0.001

¹ Adjusted for child age, sex, race/ethnicity, and federal poverty level (FPL).

Comment [H2]: Use this table structure as a guide for other tables. Redraw the tables in 7th Edition of APA format, i.e no column lines, but row lines at the topb and the bottom of the table. Please take to the correction.

Table 3.3: Adjusted Odds Ratios (aOR) for LDD by BMI Category, Stratified by Bullying Frequency

Bullying Frequency	BMI Category	aOR*	95% CI	p-value
**Never**	Underweight	1.09	0.59 – 2.03	0.781

	Overweight	1.07	0.73 – 1.56	0.745
	Obese	2.12	1.45 – 3.11	**0.000**
**Occasional**	Underweight	0.80	0.53 – 1.21	0.292
	Overweight	1.77	1.24 – 2.52	**0.002**
	Obese	1.65	1.14 – 2.39	**0.008**
**Frequent**	Underweight	0.69	0.32 – 1.45	0.324
	Overweight	1.51	0.81 – 2.81	0.194
	Obese	1.35	0.87 – 2.08	0.176

**Bullying Frequency**	**BMI Category**	**aOR[1]**	**95% CI**	**p-value**
**Never**	Underweight	1.09	0.59–2.03	0.781
	Overweight	1.07	0.73–1.56	0.745
	Obese	2.12	1.45–3.11	**<0.001**
**Occasional**	Underweight	0.80	0.53–1.21	0.292
	Overweight	1.77	1.24–2.52	**0.002**
	Obese	1.65	1.14–2.39	**0.008**
**Frequent**	Underweight	0.69	0.32–1.45	0.324
	Overweight	1.51	0.81–2.81	0.194
	Obese	1.35	0.87–2.08	0.176

[1] Adjusted for child age, sex, race/ethnicity, and federal poverty level (FPL).

Figure 1; Predicted Probability of Depression - Bullying Category

Figure 2: Predicted Probability of Depression- Mental Health of Father

Figure 3: Predicted Probability of Depression- Mental Health of Mother

Together, these stratified results suggest that the association between obesity and depression varies depending on the child’s bullying experiences and parental mental health, highlighting the moderating role of interpersonal environments.

In contrast, **difficulty making friends** and **family resilience (Table 3.4)** did not interact with obesity, but both were strong correlates of depression on their own. Children who had a little difficulty making friends had five times higher odds to report LDD (aOR 4.96; 95% CI: 4.07–6.05), and those with a lot of difficulty had almost thirteen times the odds (aOR 12.94; 95% CI: 10.03–16.44), compared to children who reported no difficulty. On the flip side, strong family resilience seemed to offer

protection. Children from families that were resilient to “most items” had 43% lower odds of LDD (aOR 0.57; 95% CI: 0.42–0.77), and those from families resilient to “some items” had 60% lower odds (aOR 0.40; 95% CI: 0.31–0.53) compared to families resilient to all items.

Table 3.4: Adjusted Odds Ratio for LDD by Difficulty in Making Friends and Family Resilience

Variable	Category	aOR[1]	95% CI
**Difficulty Making Friends**	No difficulty (reference)	1.00	1.00
	A little difficulty	4.96	4.07–6.05
	A lot of difficulty	12.94	10.03–16.44
**Family Resilience**	Resilient to all items (reference)	1.00	1.00
	To most items	0.57	0.42–0.77
	To some items	0.40	0.31–0.53

[1] Adjusted for child age, sex, race/ethnicity, and federal poverty level (FPL).

While making friends and family resilience did not interact with BMI, both remained independently associated with depression, suggesting additional pathways of psychosocial risk and buffering.

When examining the relationship of **body weight and social challenges** related to **LDA in children,** the impact of obesity appeared to vary depending on the child’s ability to make friends, and the mental health of their father. Table 4.1 shows that among children who reported **no difficulty making friends,** obesity was not significantly linked to anxiety when adjusted for age, sex, race/ethnicity and FPL (aOR 1.12; 95% CI: 0.87–1.44). The same pattern held for those with **a little difficulty,** where the odds of anxiety were slightly higher for children with obesity, but this was not statistically significant (aOR 1.27; 95% CI: 0.94–1.72). However, among children who had **a lot of difficulty making friends,** the findings were different:

children with obesity had twice the odds of experiencing anxiety compared to their normal-weight peers (aOR 1.91; 95% CI: 1.13–3.22).

Table 4: Effect of the Interpersonal Factors on the LDA among Children 6-17 years with Obesity

Table 4.1: Adjusted Odds Ratios (aOR) for LDA by BMI Category, Stratified by Difficulty in Making Friends

Difficulty in Making Friends	BMI Category	aOR[1]	95% CI	p-value
**No difficulty**	Underweight	1.05	0.80–1.39	0.724
	Overweight	1.06	0.84–1.33	0.642
	Obese	1.12	0.87–1.44	0.368
**Little difficulty**	Underweight	0.75	0.52–1.08	0.125
	Overweight	0.79	0.61–1.03	0.081
	Obese	1.27	0.94–1.72	0.121
**A lot of difficulty**	Underweight	1.34	0.79–2.28	0.277
	Overweight	1.38	0.89–2.14	0.146
	Obese	1.91	1.13–3.22	**0.015**

[1] Adjusted for child age, sex, race/ethnicity, and federal poverty level (FPL).

Table 4.2: Adjusted Odds Ratios (aOR) for LDA by BMI Category, Stratified by Father Mental Health

Father's Mental Health	BMI Category	aOR[1]	95% CI	p-value
**Excellent / Very Good**	Underweight	1.07	0.85–1.36	0.552
	Overweight	1.00	0.82–1.22	0.983
	Obese	1.19	0.95–1.48	0.126
**Good**	Underweight	0.92	0.63–1.35	0.666
	Overweight	0.78	0.59–1.04	0.089
	Obese	1.56	1.14–2.14	**0.006**
**Fair / Poor**	Underweight	1.21	0.65–2.24	0.549
	Overweight	1.55	0.95–2.54	0.078
	Obese	2.14	1.34–3.41	**0.001**

[1] Adjusted for child age, sex, race/ethnicity, and federal poverty level (FPL).

A similar trend appeared when examining **fathers' mental health (Table 4.2).** For children whose fathers reported **excellent or very good mental health,** obesity

among obese children, the confidence interval for the obesity–anxiety association crossed the null (aOR=1.19; 95% CI: 0.95–1.48), indicating limited statistical evidence for an association in this subgroup, after adjusting for age, sex, race/ethnicity and FPL. Among those whose fathers reported **good** mental health, obesity was associated with a 56% increase in odds of anxiety (aOR 1.56; 95% CI: 1.14–2.14). The most substantial effect was observed in children whose fathers had **fair or poor mental health**—here, obesity more than doubled the odds of experiencing anxiety (aOR 2.14; 95% CI: 1.34–3.41).

Thus, for LDA, the association between BMI and internalizing symptoms was strongest among children who had difficulty maintaining friendships and those whose fathers reported poorer mental health.

Figures 4 and 5 show that the likelihood of anxiety was notably higher in children with obesity when their father reported poor or fair mental health, exceeding 25%. Additionally, children who faced more challenges in forming friendships had a significantly higher risk of LDA across all BMI categories, especially among those with obesity. These results indicate that paternal mental health and social integration play a crucial role in modifying the association between BMI and LDA in children.

Figure 4 : Predicted Probability of LDA– Making Friends

Figure 5: Predicted Probability of LDA– Father's Mental Health

Discussion

Our findings are consistent with existing literature on the association between childhood obesity and severe mental health difficulties and reinforce the importance of recognizing obesity as an essential risk factor for depression and anxiety in childhood populations. It indicates how it is imperative to recognize obesity as an

essential risk factor for depression and anxiety in childhood populations (9,47,55). However, other longitudinal investigations have reported weaker or non-significant associations once baseline emotional symptoms or socioeconomic stressors are rigorously controlled, suggesting that obesity may not uniformly predict mental illness across all contexts (11,12). This research adds to the literature by revealing that interpersonal stressors exacerbate these risks, aligning with socio-ecological and diathesis-stress models. We observed heterogeneity across BMI groups, particularly between the pathways of depression and anxiety. While the literature corroborates this association for both depression and anxiety, our study has identified a nuance: BMI exhibits a stronger association with depression than with anxiety.

Although the literature recognizes bullying as a risk factor for depression and anxiety (56), especially demonstrating a dose–response relationship where more frequent victimization predicts greater internalizing risk (20,22,57), another paradox identified in this study was that infrequent bullying was more predictive of depression than frequent bullying. This interaction was absent in the development of anxiety in children. Interestingly, the link between obesity and depression was most noticeable among children who faced *occasional* bullying rather than frequent bullying. Since the study is cross-sectional, we can't explain why this pattern occurs. One possible explanation, backed by resilience research, is that ongoing or repeated adversity might trigger coping mechanisms over time, whereas infrequent or unresolved bullying could be more psychologically destabilizing.. We can hypothesize that these findings, although counterintuitive, may be due to social desirability bias or selection bias. We can assume that parents have underreported the frequency of bullying, as it engenders stigma or exposes vulnerabilities within the family, particularly when the child conceals the victimization. Nonetheless, this interpretation is speculative and

requires testing in future longitudinal studies. Consequently, we believe that our findings suggest the necessity for screening mental health issues, even amidst seemingly minor or infrequent peer conflicts.

Previous literature posited that maternal and paternal mental health have significant effects upon children's emotional regulation, attachment style, and behavior development (31,34,35). A few studies have reported weaker associations after adjusting for socioeconomic stressors, and found that genetic heritability may partially account for these relationships—indicating parental mental health effects are not uniformly environmental (30,33). The literature supported this phenomenon was more commonly observed in mother-child relationships than in father-child relationships. The strong link between poor maternal mental health and child depression in our results likely reflects the enduring influence of traditional caregiving roles. As primary caregivers, mothers often provide most of the emotional, educational, and health-related support. Depressed or anxious mothers may be less responsive, nurturing, or emotionally attuned to their children and therefore contribute to poorer caregiving quality, which then indirectly affects the psychosocial development of children, but this would be outside the scope of our research. Mothers face an outsize share of the spadework when it comes to scheduling, cares and comforting as a career so often, they are still very much the hub around which the child's social and emotional world whirls.

Despite other interpersonal stresses, resilience appears to be a buffer against depression (58,59). Studies reported inconsistent or limited moderation effects, showing that resilience does not uniformly protect against anxiety or chronic adversity (15,39). Our findings indicate that, while family resilience is a powerful

buffer against depression in children themselves classified as obese, it is not a powerful buffer against anxiety. The distinction may be traced back to the different cognitive and affective mechanisms underpinning these mental health issues. Depression is characterized by a negative schema of hopelessness and self-blame, which may improve more with cohesive family processes. In contrast, anxiety—associated with hyperarousal and vigilance—may necessitate non-family-centred interventions.

The importance of multi-level approaches that consider interpersonal context is reinforced by our findings. Paediatric screening practices could incorporate routine assessment of peer relationship difficulties and parental mental health where appropriate. School-based anti-bullying programs and caregiver-focused supports may help address some of the interpersonal environments associated with children's emotional well-being. Parent-focused interventions could enhance children's emotional well-being, while school-based anti-bullying and peer-bonding programs—especially targeting weight-based discrimination—may address key social risk factors. Policy approaches may address the disproportionate burden on low-income and minority families, embedding obesity and mental health services in equity frameworks. At a policy level, the findings suggest that holistic approaches may be considered for children's health, taking into account both physical and mental aspects. Policymakers may also recognize that the mental health disparities associated with obesity differentially impact low-income and minority families, and therefore are of relevance to an equity framework that informs the process of planning and budgeting programs. While the NSCH dataset does not directly evaluate the effectiveness of interventions, these results highlight potential domains where supportive policies could be relevant.

While providing additional insights into the associations between psychosocial determinants and childhood obesity as well as mental health, several limitations should be acknowledged. The NSCH design is cross-sectional and does not support causal inference. The reliance on parent reports of mental health symptoms and interpersonal behavior (e.g., bullying) may result in recall bias and social desirability. Additionally, given the number of stratified tests, there is an increased risk of type I error; thus, these interaction findings should be viewed as suggestive and requiring confirmation in future studies. The study was limited in its generalizability due to the U.S.-specific health, social, and policy context. Future research should utilize longitudinal designs to establish causal directions, involve multiple informants to increase validity, and include qualitative or mixed-method approaches to explain counterintuitive findings such as the lack of a dose–response relationship for infrequent bullying. Future research should confirm the generalizability of these associations in comparative studies across diverse cultural and structural contexts.

In sum, obesity in children is embedded within a network of interpersonal and contextual influences that differentially predict depression and anxiety. Multi-level, theory-driven interventions spanning healthcare, education, and family systems are essential to address these intertwined physical and mental health challenges.

Acknowledgements

The author sincerely thanks Dr. Madhivanan, Dr. Chen, Dr. Suh, Dr. Scott Carvajal, Dr. Karl Krupp, and Prof. Maia for their mentorship and guidance in statistics. Gratitude is also expressed to the Arizona Department of Health Services (AZDHS) for their continuous partnership and support in developing this dissertation project.

Additionally, the author values the technical infrastructure supplied by the University of Arizona, which facilitated computational and data management needs.

References

1. World Health Organization [WHO]. Obesity and overweight [Internet]. 2024 [cited 2024 Jul 2]. p. 76. Available from: https://www.who.int/news-room/fact-sheets/detail/obesity-and-overweight#:~:text=For adults%2C WHO defines overweight and obesity as,to be considered when defining overweight and obesity.
2. CDC US Government. Childhood Obesity Facts [Internet]. 2024. 2024 [cited 2025 Mar 15]. Available from: https://www.cdc.gov/obesity/childhood-obesity-facts/childhood-obesity-facts.html
3. Di Cesare M, Sorić M, Bovet P, Miranda JJ, Bhutta Z, Stevens GA, et al. The epidemiological burden of obesity in childhood: a worldwide epidemic requiring urgent action. BMC Med [Internet]. 2019;17(1):212. Available from: https://doi.org/10.1186/s12916-019-1449-8
4. Rankin J, Matthews L, Cobley S, Han A, Sanders R, Wiltshire H, et al. Psychological consequences of childhood obesity: psychiatric comorbidity and prevention. Adolesc Health Med Ther [Internet]. 2016; Available from: https://pubmed.ncbi.nlm.nih.gov/27881930
5. Choi H, Corredor-Waldron A, Currie J, Felton C. WHAT CAN TRENDS IN EMERGENCY DEPARTMENT VISITS TELL US ABOUT CHILD MENTAL HEALTH? Natl Bur Econ Res [Internet]. 2025; Available from: https://www.medicaid.gov/medicaid/benefits/early-and-periodic-screening-diagnostic-and-treatment
6. NIHCM Foundation. The State of Children's Health in the United States

[Internet]. 2023. 2023 [cited 2025 Mar 15]. Available from: https://nihcm.org/publications/the-state-of-childrens-health-in-the-united-states
7. Quek Y-H, Tam WWS, Zhang MWB, Ho RCM. Exploring the association between childhood and adolescent obesity and depression: a meta-analysis. Obes Rev an Off J Int Assoc Study Obes. 2017 Jul;18(7):742–54.
8. Fan Y, Wang D, Wang W, Chen H, Lin J, Lin W, et al. Depression, anxiety, and development of obesity from childhood to young adulthood in China. J Psychosom Res. 2024 Oct;185:111867.
9. Chen Y, Zhang J, Yuan L, Hu H, Li T, Zhao Y, et al. Obesity and risk of depressive disorder in children and adolescents: A meta-analysis of observational studies. Child Care Health Dev. 2024 Mar;50(2):e13237.
10. Sutaria S, Devakumar D, Yasuda SS, Das S, Saxena S. Is obesity associated with depression in children? Systematic review and meta-analysis. Arch Dis Child. 2019;104(1):64–74.
11. Mannan MA, Mamun A Al, Doi SAR, Clavarino A. Prospective Associations between Depression and Obesity for Adolescent Males and Females- A Systematic Review and Meta-Analysis of Longitudinal Studies. PLoS One [Internet]. 2016; Available from: https://pubmed.ncbi.nlm.nih.gov/27285386
12. Marmorstein NR, Iacono WG, Legrand LN. Obesity and depression in adolescence and beyond: reciprocal risks. Int J Obes [Internet]. 2014; Available from: https://pubmed.ncbi.nlm.nih.gov/24480863
13. Luppino FS, Wit L de, Bouvy PF, Stijnen T, Cuijpers P, Penninx BWJH, et al. Overweight, Obesity, and Depression A Systematic Review and Meta-analysis of Longitudinal Studies. Arch Gen Psychiatry [Internet]. 2010; Available from: https://pubmed.ncbi.nlm.nih.gov/20194822

14. Puhl RM, King KM. Weight discrimination and bullying. Best Pract Res Clin Endocrinol Metab. 2013 Apr;27(2):117–27.
15. Mesman E, Vreeker A, Hillegers M. Resilience and mental health in children and adolescents: An update of the recent literature and future directions. Curr Opin Psychiatry. 2021;34(6):586–92.
16. Buro AW, Salinas-Miranda A, Marshall J, Gray HL, Kirby RS. Obesity and neurodevelopmental and mental health conditions among adolescents aged 10–17 years: The National Survey of Children's Health 2017–2018. J Paediatr Child Health. 2022;58(10):1753–9.
17. Cheng S, Kaminga AC, Liu Q, Wu F, Wang Z, Wang X, et al. Association between weight status and bullying experiences among children and adolescents in schools: An updated meta-analysis. Child Abuse Negl [Internet]. 2022; Available from: https://doi.org/10.1016/j.chiabu.2022.105833
18. Blanco M, Solano S, Alcántara AI, Parks M, Román FJ, Sepúlveda AR. Psychological well-being and weight-related teasing in childhood obesity: a case-control study. Eat Weight Disord. 2020 Jun;25(3):751–9.
19. Şahın N, Kırlı U. The Relationship Between Peer Bullying and Anxiety-Depression Levels in Children With Obesity. Anatol J Psychiatry [Internet]. 2020; Available from: https://pubmed.ncbi.nlm.nih.gov/36425935
20. Pryor L, Brendgen M, Boivin M, Dubois L, Japel C, Falissard B, et al. Overweight during childhood and internalizing symptoms in early adolescence: The mediating role of peer victimization and the desire to be thinner. J Affect Disord [Internet]. 2016; Available from: https://pubmed.ncbi.nlm.nih.gov/27267292
21. Vuuren CL van, Wachter G, Veenstra R, Rijnhart JJM, Wal MF van der,

Chinapaw MJM, et al. Associations between overweight and mental health problems among adolescents, and the mediating role of victimization. BMC Public Health [Internet]. 2019; Available from: https://pubmed.ncbi.nlm.nih.gov/31113424

22. Rupp K, McCoy SM. Bullying Perpetration and Victimization among Adolescents with Overweight and Obesity in a Nationally Representative Sample. Child Obes [Internet]. 2019; Available from: https://pubmed.ncbi.nlm.nih.gov/31062988

23. Kozasa S, Oiji A, Kiyota A, Sawa T, Kim S-Y. Relationship between the experience of being a bully/victim and mental health in preadolescence and adolescence: a cross-sectional study. Ann Gen Psychiatry [Internet]. 2017; Available from: https://pubmed.ncbi.nlm.nih.gov/29075310

24. Wood MA. Friendship Security and Intimacy Moderate the Stability of Anxiety During Early Adolescence. 2013.

25. Adedeji A, Otto C, Kaman A, Reiss F, Devine J, Ravens-Sieberer U. Peer Relationships and Depressive Symptoms Among Adolescents: Results From the German BELLA Study. Front Psychol. 2022;12(January).

26. Gorrese A. Peer Attachment and Youth Internalizing Problems: A Meta-Analysis. Child Care Q. 2016;

27. Zimmer-Gembeck MJ, Clear SJ, Campbell SM. Peer relationships and stress: Indirect associations of dispositional mindfulness with depression, anxiety and loneliness via ways of coping. J Adolesc. 2021;

28. Chiu K, Clark DM, Leigh E. Prospective associations between peer functioning and social anxiety in adolescents: A systematic review and meta-analysis. J Affect Disord. 2020;

29. Lampard AM, Franckle RL, Davison KK. Maternal depression and childhood obesity: A systematic review. Prev Med (Baltim). 2014;59(1):60–7.

30. Foster BA, Weinstein K, Mojica CM, Davis MM. Parental Mental Health Associated With Child Overweight and Obesity, Examined Within Rural and Urban Settings, Stratified by Income. J Rural Heal Off J Am Rural Heal Assoc Natl Rural Heal Care Assoc. 2020 Jan;36(1):27–37.

31. Goodman SH, Rouse MH, Connell AM, Broth MR, Hall CM, Heyward D. Maternal Depression and Child Psychopathology: A Meta-Analytic Review. Clin Child Fam Psychol Rev [Internet]. 2011;14(1):1–27. Available from: https://doi.org/10.1007/s10567-010-0080-1

32. Wang L, Anderson JL, Dalton W, Wu T, Liu X, Zheng S, et al. Maternal Depressive Symptoms and the Risk of Overweight in Their Children. Matern Child Health J [Internet]. 2013; Available from: https://pubmed.ncbi.nlm.nih.gov/22833333

33. Hope S, Micali N, Deighton J, Law C. Maternal mental health at 5 years and childhood overweight or obesity at 11 years: evidence from the UK Millennium Cohort Study. Int J Obes [Internet]. 2019; Available from: https://doi.org/10.1038/s41366-018-0252-5

34. Connell AM, Goodman SH. The association between psychopathology in fathers versus mothers and children's internalizing and externalizing behavior problems: a meta-analysis. Psychol Bull. 2002 Sep;128(5):746–73.

35. Kluczniok D, Boedeker K, Hindi Attar C, Jaite C, Bierbaum A-L, Fuehrer D, et al. Emotional availability in mothers with borderline personality disorder and mothers with remitted major depression is differently associated with psychopathology among school-aged children. J Affect Disord. 2018

Apr;231:63–73.

36. Choi J. The Moderating Role of Family Resilience in the Relationship of Bullying Victimization, Adverse Childhood Experiences, and Poverty Status to Adolescents' Internalizing Disorders. J Aggress Maltreat Trauma. 2022;

37. Uddin J, Ahmmad Z, Uddin H, Tatch A. Family resilience and protective factors promote flourishing and school engagement among US children amid developmental disorder and adverse psychosocial exposure. Sociol Spectr. 2021;

38. Zhuo R, Yu Y, Shi X. Family Resilience and Adolescent Mental Health during COVID-19: A Moderated Mediation Model. Int J Environ Res Public Health. 2022;

39. Ao L, Cheng X, An D, An Y, Yuan G. Relationship between Perceived Family Resilience, Emotional Flexibility, and Anxiety Symptoms: a Parent-Adolescent Dyadic Perspective. J Youth Adolesc. 2024;

40. Bethell C, Gombojav N, Whitaker RC. Family Resilience And Connection Promote Flourishing Among US Children, Even Amid Adversity. Health Aff. 2019;

41. Ghandour R, Sherman L, Vladutiu C, Ali M, Lynch S, Bitsko S. Prevalence and Treatment of Depression, Anxiety, and Conduct Problems in US Children. J Pediatr [Internet]. 2019;206:256–67. Available from: https://pmc.ncbi.nlm.nih.gov/articles/PMC6673640/pdf/nihms-1009660.pdf

42. Bronfenbrenner U. Mental Health and Well-being: A Socio-Ecological Model [Internet]. University of Minnesota School of Public Health. 2021. p. 1–2. Available from: https://www.telethonkids.org.au/globalassets/media/documents/projects/sew---

arts/the-social-ecological-model-2.2021.pdf

43. Golden SD, Earp JAL. Social Ecological Approaches to Individuals and Their Contexts: Twenty Years of Health Education & Behavior Health Promotion Interventions. Heal Educ Behav. 2012;39(3):364–72.

44. Belsky J, Jonassaint C, Pluess M, Stanton M, Brummett B, Williams R. Vulnerability genes or plasticity genes? Mol Psychiatry. 2009;14(8):746–54.

45. Pierson C. The Social Environment, Genetics, And Obesity: Evaluation of Diathesis-Stress and Differential Susceptibility Frameworks. 2013.

46. Avila C, Holloway AC, Hahn MK, Morrison KM, Restivo M, Anglin R, et al. An Overview of Links Between Obesity and Mental Health. Curr Obes Rep. 2015;4(3):303–10.

47. Beltrán-Garrayo L, Solar M, Blanco M, Graell M, Sepúlveda AR. Examining associations between obesity and mental health disorders from childhood to adolescence: A case-control prospective study. Psychiatry Res. 2023;326(March).

48. National Survey of Children’s Health. Request an NSCH Data Set-Data Resource Center for Child and Adolescent Health-A project of the Child and Adolescent Health Measurement Initiative [Internet]. Available from: https://www.childhealthdata.org/dataset

49. HHS. 2021-2022 National Survey of Children’s Health. 2024.

50. Cree Robyn A, Bitsko Rebecca H, Danielson Melissa L, Wanga Valentine, Holbrook Joseph, Flory Kate, et al. Surveillance of ADHD Among Children in the United States: Validity and Reliability of Parent Report of Provider Diagnosis. J Atten Disord [Internet]. 2022 Nov 3;27(2):111–23. Available from: https://doi.org/10.1177/10870547221131979

51. Visser SN, Danielson ML, Bitsko RH, Holbrook JR. Convergent Validity of Parent-Reported ADHD Diagnosis: A Cross-Study Comparison. Ann Epidemiol. 2013;23(9):592.

52. Ebesutani C, Tottenham N, Chorpita B. The Revised Child Anxiety and Depression Scale - Parent Version: Extended Applicability and Validity for Use with Younger Youth and Children with Histories of Early-Life Caregiver Neglect. J Psychopathol Behav Assesss. 2015;37(4):705–18.

53. Blumberg SJ, Foster EB, Frasier AM, Et Al. Program and Collection Procedures Design and Operation of the National Survey of Children's Health, 2007. 2009;109.

54. US Census Bureau. 2021 National Survey of Children's Health: Source and Accuracy Statement [Internet]. 2021. Available from: https://www2.census.gov/programs-surveys/nsch/technical-documentation/source-and-accuracy/2021-NSCH-Source-and-Accuracy-Statement.pdf

55. Lindberg L, Hagman E, Danielsson P, Marcus C, Persson M. Anxiety and depression in children and adolescents with obesity: A nationwide study in Sweden. BMC Med. 2020;18(1):1–9.

56. Foody M, Samara M, O'Higgins Norman J. Bullying by siblings and peers: Poly-setting victimization and the association with problem behaviours and depression. Br J Educ Psychol. 2020 Jun;90 Suppl 1:138–57.

57. Van Vuuren CL, Wachter GG, Veenstra R, Rijnhart JJM, van der Wal MF, Chinapaw MJM, et al. Associations between overweight and mental health problems among adolescents, and the mediating role of victimization. BMC Public Health. 2019;

58. Anderson JR, Mayes TL, Fuller A, Hughes JL, Minhajuddin A, Trivedi MH. Experiencing bullying's impact on adolescent depression and anxiety: Mediating role of adolescent resilience. J Affect Disord. 2022 Aug;310:477–83.

59. Lin L, Chien Y, Chen Y, Wu C, Chiou H. Bullying Experiences , Depression , and the Moderating Role of Resilience Among Adolescents. 2022;10(May):1–11.

Figure 1

Click here to access/download;Figure;Plos - Figure 1.tif

Figure 1: Predicted Probability of Depression - Bullying Category

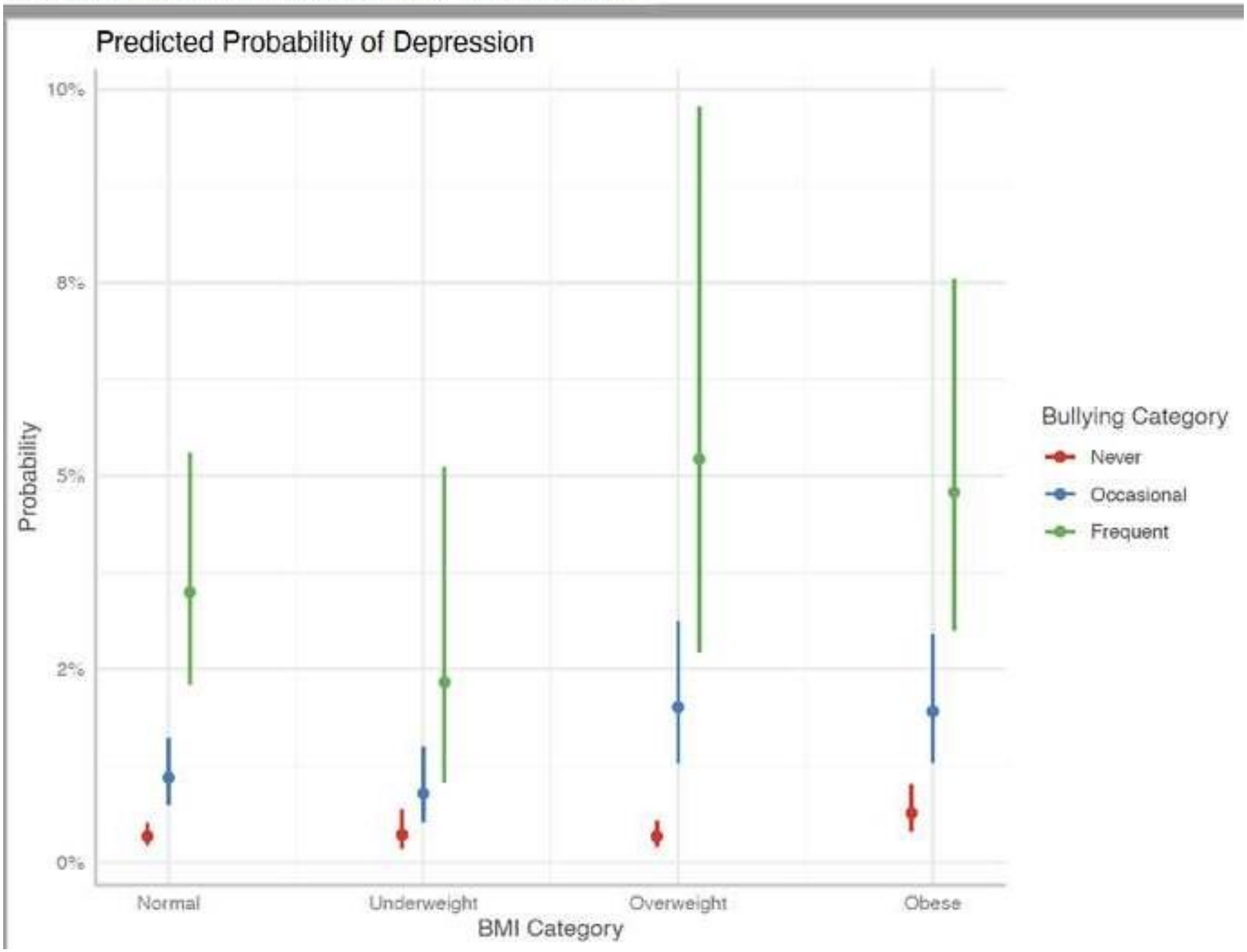

Figure 2

Click here to access/download;Figure;Plos - Figure 2.tif

Figure 2: Predicted Probability of Depression - Mental health of Father

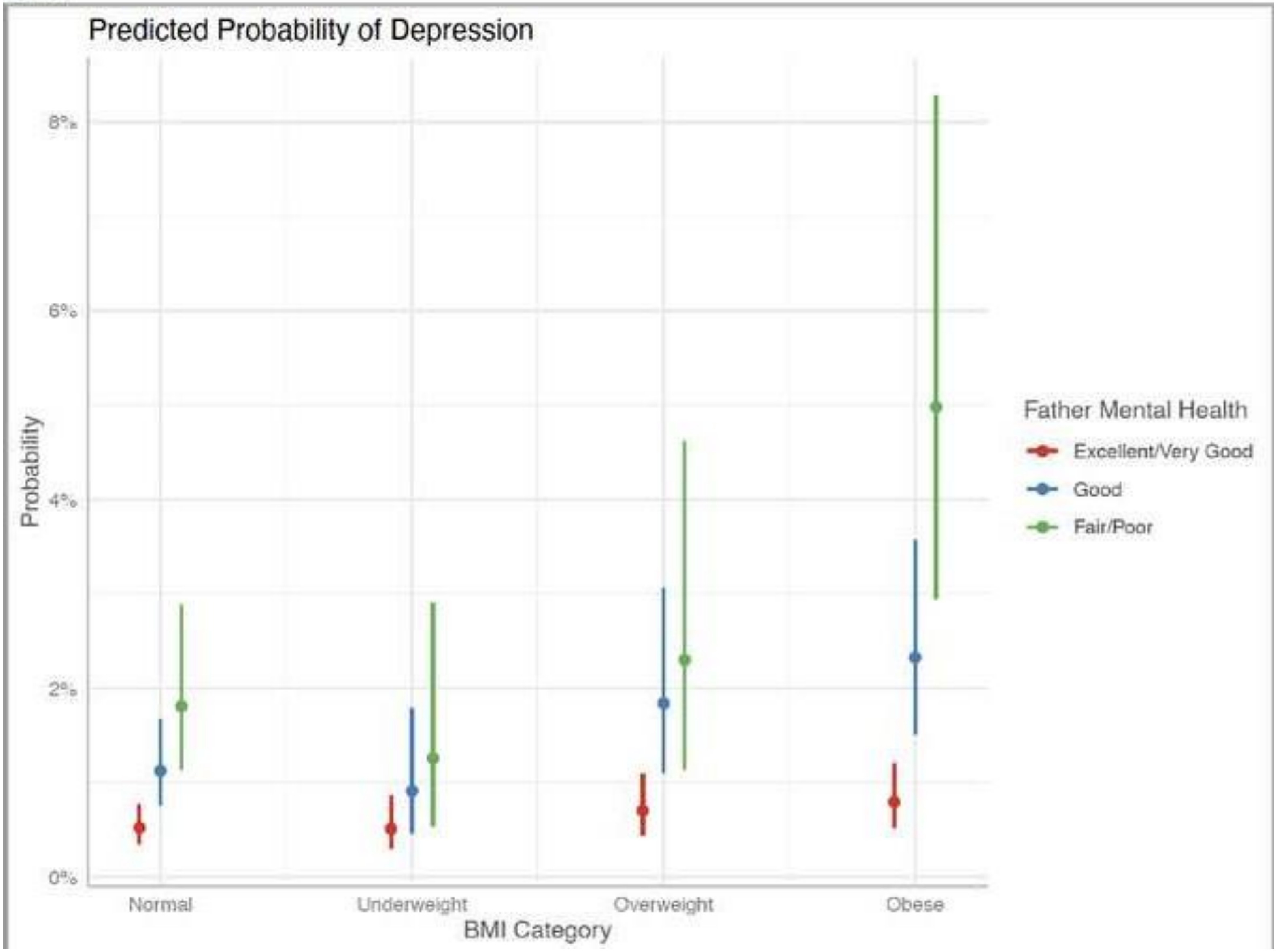

Figure 3

Click here to access/download;Figure;Plos - Figure 3.tif

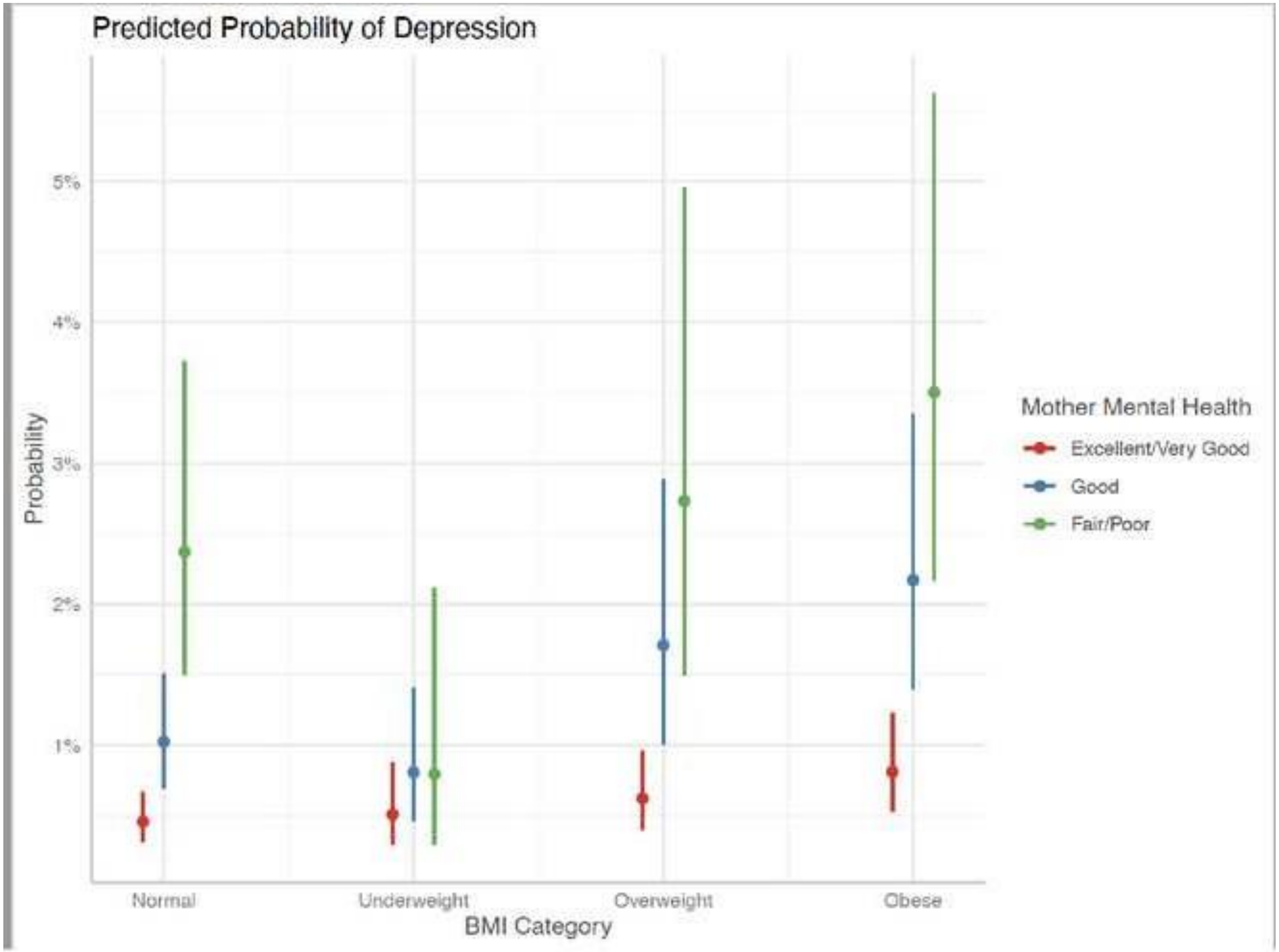

Figure 3: Predicted Probability of Depression - Mental Health of Mother

Figure 4

Click here to access/download;Figure;Plos - Figure 4.tif

Figure 4: Predicted Probability of Anxiety - Making Friends

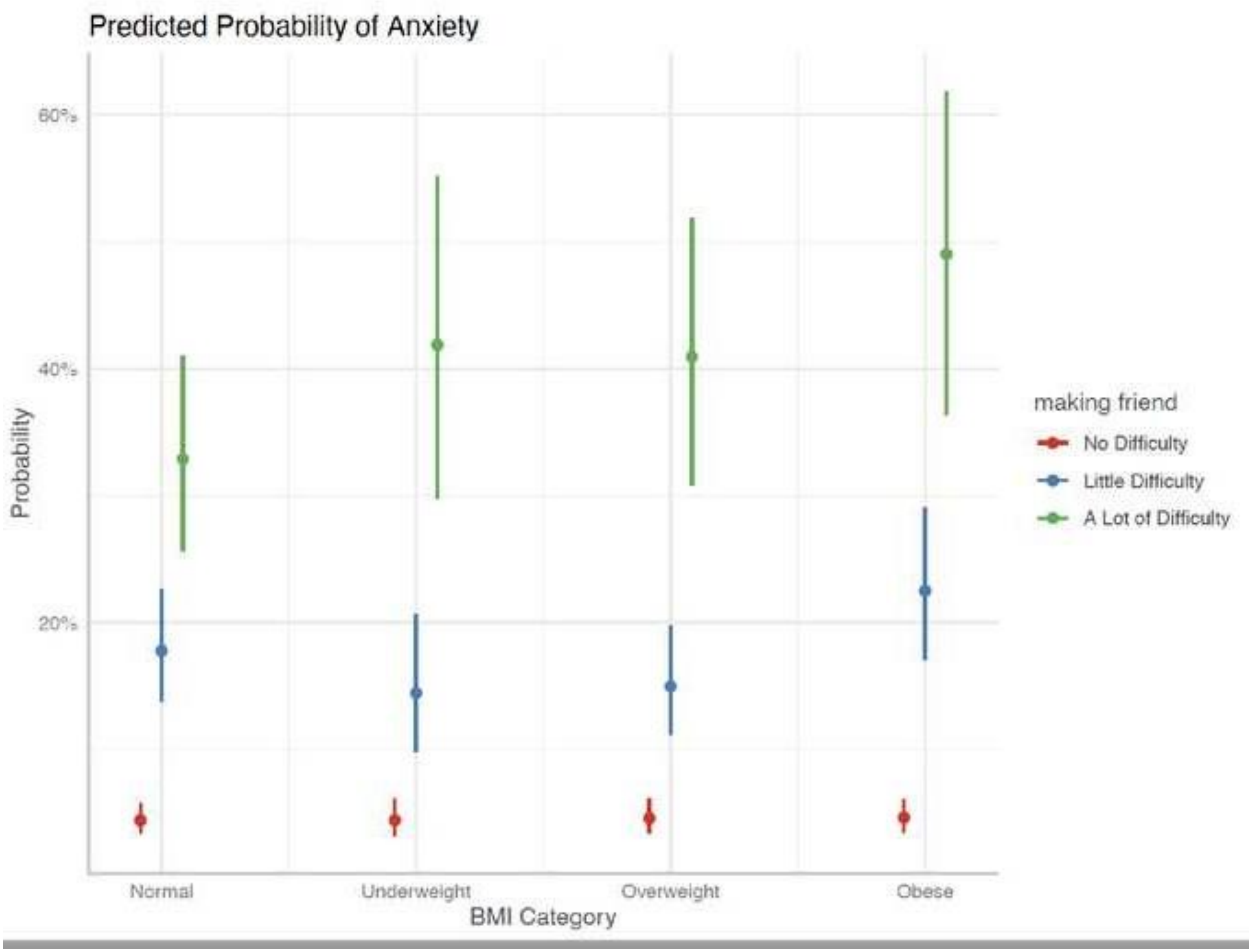

Figure 5

Click here to access/download;Figure;Plos - Figure 5.tif

Figure 5: Predicted Probability of Anxiety - Father's Mental Health

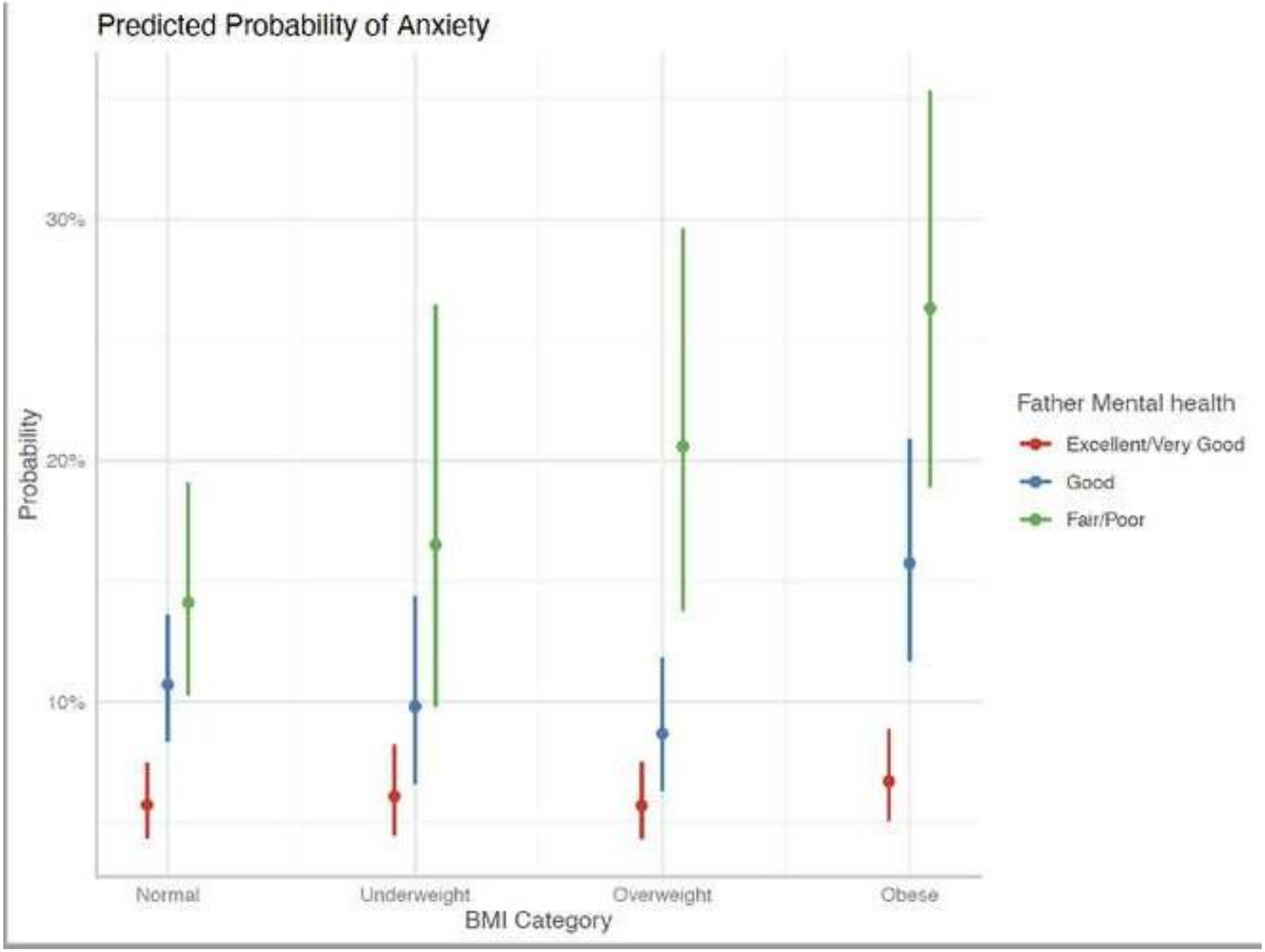

Between the Scales and the Scars: The Interpersonal Burden of Body Weight on Children's Mental Health: Evidence from the 2021- 22 National Survey of Children's Health (NSCH)

Fnu Kajal[1], Bin Suh[2], Chen Zhao[3], Maia Ingram[4], , Scott Carvajal[5], Karl Krupp[6], Purnima Madhivanan[7]

Corresponding Author's Details:

[1]Fnu Kajal, M.D., M.Sc., DrPH Candidate, Mel & Enid Zuckerman College of Public Health, University of Arizona.
Email: kkajal@arizona.edu
ORCiD: https://orcid.org/0000-0002-7971-0237

[2]Bin Suh, Senior Epidemiologist, Arizona Department of Health Services.
Email: bin.suh@azdhs.gov
ORCiD: https://orcid.org/0000-0001-8853-4709

[3]Zhao Chen, PhD, MPH, Distinguished Professor, Epidemiology and Biostatistics, Mel & Enid Zuckerman College of Public Health, University of Arizona.
Email: zchen@arizona.edu
ORCiD: https://orcid.org/0000-0002-5659-0447

[4]Maia Ingram, MPH, Director of Participatory Research and Practice and Co-Director, Arizona Prevention Research Center, Mel & Enid Zuckerman College of Public Health, University of Arizona.
Email: maiai@arizona.edu
ORCiD: https://orcid.org/0000-0002-6517-7697

[5]Scott Carvajal, PhD, Departmental Chair and Professor, Health Promotion Sciences, Mel & Enid Zuckerman College of Public Health, University of Arizona..
Email: carvajal@arizona.edu
ORCiD: https://orcid.org/0009-0003-2106-5305

[6]Karl Krupp, MSc, PhD, Assistant Professor, Division of Public Health Practice & Translational Research, Mel & Enid Zuckerman College of Public Health, University of Arizona.
Email: kkrupp@arizona.edu
ORCiD: https://orcid.org/0000-0001-7001-9194

[7]Purnima Madhivanan, MBBS, MPH, PhD, Associate Professor, Health Promotion Sciences, Mel & Enid Zuckerman College of Public Health, University of Arizona.
Email: pmadhivanan@arizona.edu
ORCiD: https://orcid.org/0000-0001-7818-3394

Formatted: Font: (Default) Arial
Formatted: Font: (Default) Arial
Formatted: Font: (Default) Arial
Formatted: Font: (Default) Arial
Formatted: Font: (Default) Arial
Formatted: Font: (Default) Arial
Formatted: Font: (Default) Arial
Formatted: Font: (Default) Arial
Formatted: Font: (Default) Arial
Formatted: Font: (Default) Arial
Formatted: Font: (Default) Arial
Formatted: Font: (Default) Arial
Formatted: Font: (Default) Arial
Formatted: Font: (Default) Arial

Abstract

Background:

Childhood mental health issues have~~obesity has~~ emerged as a significant public health concern, with an increasing body of evidence ~~correlating it with~~linking them to a higher~~eightened~~ risk of ~~lifelong diagnosis of depression and anxiety(LDD/LDA)~~children's obesity. This study aims to examine the impact of less-explored interpersonal dynamics on the association between obesity and LDA/LDD ~~lifetime diagnoses of depression and anxiety~~ in children.

Methods:

A cross-sectional study of 40,820 U.S. children aged 6-17 used survey-weighted logistic regression on the 2021-22 NSCH data. The study examined the moderating effects of bullying, peer relationships, parental mental health, and family resilience on mental health outcomes (LDD & LDA), utilizing interaction terms categorized by Body Mass Index (BMI), adjusted for age, sex, race/ethnicity, and poverty levels.

Results:

Obesity was significantly associated with higher odds of both **depression** and **anxiety** in children aged 6-17 years. Obese children had **2.08 times higher odds** of depression (aOR 2.08; 95% CI: 1.64-2.64) and **1.48 times higher odds** of anxiety (aOR 1.48; 95% CI: 1.25-1.76) compared to normal-weight peers. Significant moderating effects were observed for **depression (adjusted for age, sex, race/ethnicity and poverty levels)**: never bullied (aOR = 2.12; 95% CI: 1.45-3.11), maternal mental health (aOR = 1.70; 95% CI: 1.70- 2.70), and paternal mental health (aOR = 2.54; 95% CI: 1.51-4.29). For **anxiety**, moderating effects were seen for

making friends (aOR = 1.91; 95% CI: 1.13-3.22) and **father mental health** (aOR = 2.14; 95% CI: 1.34-3.41).

Conclusions:

Obesity is associated with interpersonal stressors, especially bullying and adverse familial environments, which, in turn, significantly escalate the risk of the development of depression and anxiety in children. Public health interventions must focus on addressing modifiable relational environments, including bullying and familial relationships, to help disrupt the pathways ~~connecting~~ through which obesity contributes to~~with~~ mental illness in children.

Introduction:

Childhood obesity has emerged as a pressing public health concern with significant implications for both physical and mental well-being. On a global scale, childhood obesity has proliferated at an unprecedented rate in recent decades. According to the World Health Organization, it was estimated in 2022 that more than 390 million children aged 5 to 19 were classified as overweight, and 160 million were obese (1). In the United States, nearly 20 percent of children experience obesity, and the rates of childhood obesity have increased fourfold since 1990 (2), forming what is increasingly recognized as a "Body–Mind Burden"(3,4). Concomitantly, the incidence of mental health issues in children has spiked, with nearly 50% of adolescents experiencing at least one mental illness, and emergency visits for mental health issues rising by 37% in recent years (5,6).

This epidemiological convergence is not coincidental. There is increasing evidence for a bidirectional relationship between obesity and mental health. Children with excess weight are more likely to experience depressive symptoms, anxiety, and

Formatted: Font: (Default) Arial

Formatted: Font: (Default) Arial, Not Bold

social withdrawal, as shown across multiple meta-analyses (7–10). ~~Individual meta-analyses suggest that children who are overweight or obese are at an elevated risk of experiencing depression (7,8), anxiety (9), and social withdrawal (10).~~ The reverse also holds: adolescents with depression have a 70% increased risk of obesity, particularly among females (11,12)~~(11,12)~~.. Luppino et al. established this reciprocal risk in a seminal meta-analysis, finding that obesity heightens depression risk, and vice versa (13)~~(13)~~.

Formatted: Font: (Default) Arial

Formatted: Font: (Default) Arial

Field Code Changed

Field Code Changed

Although biological and behavioural determinants (e.g., dietary intake and physical inactivity) play a role in these associations, they do not fully account for the emotional distress presented by children with obesity. Instead, the pathways seem to be socially mediated. Teasing/Bullying, stigma, parental mental health, family routines, and peer dynamics are increasingly implicated as effect modifiers in this relationship (14–16)~~(14–16)~~. Yet, few studies have comprehensively modelled these interpersonal variables.

Field Code Changed

Among these, bullying emerges as an exceptionally robust interpersonal risk factor. Children with obesity have up to 46% higher odds of being bullied, compared to their healthy-weight peers (17)~~(17)~~.

Field Code Changed

According to the standards established by the World Health Organization (WHO), obesity is defined as a measurement that surpasses two standard deviations above the WHO growth reference median (World Health Organization [WHO], 2024). The Centers for Disease Control and Prevention (CDC) defines childhood obesity as having a body mass index (BMI) that is at or exceeds the 95th percentile for age and sex (CDC US Government, 2024).

Victimization, especially related to weight, can exacerbate internalized stigma and trigger depressive and anxious symptomatology (18,19)(18,19). Longitudinal studies show that the mental health impact of obesity is significantly magnified among children who are bullied (20,21)(20,21). Additionally, children classified as bully-victims experience the highest rates of depression, suicidal ideation, and behavioural problems (22,23)(22,23). Although bullying is widely acknowledged as linked to obesity and mental health issues, few studies have analyzed bullying together with other interpersonal and family factors within a single framework, especially using nationally representative data.

Field Code Changed

Field Code Changed

Field Code Changed

Peer relationships more broadly also serve as salient moderators. Quality friendships and strong peer attachment buffer children against the psychological impact of social exclusion due to obesity (24,25)(24,25). Increasing evidence indicates, that secure peer attachment is protective for emotional health and well-being (26)(26), whilst peer rejection evokes distress both within and outside the context of overt bullying (27,28)(27,28).

Field Code Changed

Field Code Changed

Field Code Changed

In the familial sphere, parental mental health—particularly maternal depression—has consistently been linked to both child obesity and mental health issues. Maternal depression influences parenting styles, feeding practices, emotional availability, and home routines, all of which mediate the pathway from obesity to poor emotional outcomes (29–31)(29–31). The risk is heightened with chronic depressive symptoms, as shown in cohort studies and meta-analyses (32,33)(32,33). However, paternal mental health remains underexplored, and few studies have disaggregated effects by caregiver role or gender (34,35)(34,35). The impact of paternal mental health

Field Code Changed

Field Code Changed

Field Code Changed

Formatted: Font: Not Bold

remains relatively understudied, with limited analyses distinguishing effects across different caregiver roles. Emerging evidence indicates that fathers' psychological well-being may have unique influences on a child's emotional development.

Family resilience—defined as the ability of families to adapt positively to adversity—has also emerged as a protective factor. Findings from studies that used the NSCH data have shown that family resilience can attenuate the deleterious effects of bullying and adversity on internalizing symptoms (36,37)(36,37). For example, a study using data from the COVID-19 pandemic found that family functioning was positively related to adolescent anxiety and depression(38,39)(38,39). Yet, its role in buffering the psychological consequences of obesity has been underexplored, with few studies testing interaction effects in nationally representative datasets (40,41).

Overall, previous research indicates that obesity might interact with the social environment to influence children's emotional health, but few studies have simultaneously explored bullying, peer relations, parental mental health, and family resilience as factors moderating this link. To fill this gap, this study utilizes nationally representative NSCH 2021–22 data and a socio-ecological approach to examine how these interpersonal elements impact the relationship between BMI and LDD/LDA diagnoses in children aged 6–17.

Theoretical Framework:

Mental health conditions in children are multifactorial, necessitating multidimensional theoretical frameworks. This research is grounded in two complementary frameworks—the socio-ecological model and the diathesis-stress model—which

Formatted: Font: (Default) Arial

Field Code Changed

Field Code Changed

Formatted: Normal (Web)

Formatted: Font: (Default) Arial

explain how individual predispositions intersect with interpersonal and contextual stressors to affect mental healthadopts both the **socio-ecological model (SEM)** and the **diathesis-stress model** to conceptualize the interplay between obesity and mental health outcomes in youth.

The **SEM**, originally proposed by Bronfenbrenner and expanded by McLeroy et al. into five levels of influence—Intrapersonal, Interpersonal, Institutional, Community, and Public Policy—underscores the dynamic interrelations between individuals and their environments (42,43). While intrapersonal factors like sleep and activity patterns are essential, interpersonal stressors such as bullying, parental mental health, peer isolation, and family resilience are increasingly recognized as critical modifiers of mental health outcomes in children with obesity.

The diathesis-stress model dovetails with this ecological lens and explains how genetic and physiological predispositions intersect with environmental adversities to create mental illness (44,45). We view obesity in this framework as a biological condition and psychosocial stressor that is experienced with stigma and social rejection. Nonetheless, these adversities may be ameliorated by protective interpersonal factors (e.g., emotionally balanced parenting and positive peer support). In tandem, such models provide a comprehensive framework for understanding how relational life experiences impact the emotional consequences of childhood obesity.

A recent investigation identified the omission of considering social factors, such as bullying, peer relationships, and family environment, as a crucial issue (46,47). To address these critical gaps, the present study will explore how interpersonal variables—bullying, peer relationships, parental mental health, and family resilience—influence the association between BMI and mental health outcomes using

Formatted: Font: (Default) Arial

Formatted: Font: (Default) Arial

Field Code Changed

data from the 2021–22 National Survey of Children's Health (NSCH). To account for this, we employed the socio-ecological framework and survey-weighted logistic regression models that examined both main effects and interaction effects. This nested approach provided a valuable understanding of the relational and psychosocial dimensions of obesity and mental health in childhood, informing interventions that work beyond individual behavior to include family, peer, and social aspects.

METHODOLOGY

Data Source

The data for this study were obtained from the Child and Adolescent Health Measurement Initiative (CAHMI)/NSCH for the 2021-2022 period, which is publicly available (48). The NSCH, sponsored by the U.S. Department of Health and Human Services, is conducted by the Census Bureau and has been providing nationally representative data on children's health since 2002-2003. The survey uses instruments whose reliability and validity have been supported in numerous studies.

Study Design

This study is a secondary data analysis of a cross-sectional survey examining the association between obesity and mental health issues, particularly depression and anxiety. It focuses on identifying key interpersonal determinants that modify the association between obesity and mental health issues in children.

Study Population

The study population consists of children and adolescents aged 6 to 17, as reported in the 2021-2022 NSCH survey (49). This diverse and nationally representative sample highlights a critical developmental period where obesity and mental health intersect, making it an ideal setting for investigating these issues and ensuring credible, actionable data to support research objectives.

The current research represented a sample size of 40,820 children, providing nationally representative data on their health and well-being. Data collection occurred between June 2021 and January 2022. The participants were the parents and guardians of children. The data were collected via the web (90%) and mail (10%), with an approximate response rate of 65%. ~~About 90% of the sample received incentives.~~

Data Collection Tools/Measures: All measures were parent-reported (49). This may introduce the potential for reporting bias, such as misclassification of BMI. However, many previous studies on NSCH data have indicated the acceptable validity of parent-reported measures (50,51)

Outcomes (Mental Health Measures): The outcome variables were ‘depression’ and ‘anxiety’ as assessed using the Revised Child Anxiety and Depression Scale (52). Regarding depression and anxiety, responses encompass the current diagnosis and historical data for children aged 6-17. The responses related to depression and anxiety were classified as binary responses for the purpose of analysis. Given the limited sample size for current diagnoses, we combined current and lifetime indicators to preserve statistical power; however, this approach may obscure temporal differences. Lifetime diagnosis of depression (LDD)/ Lifetime Diagnosis of Anxiety (LDA) refers to children who have ever been diagnosed with

Formatted: Font: (Default) Arial

Formatted: Font: (Default) Arial

depression/anxiety, including those who are currently depressed, and those who were previously diagnosed but no longer meet the criteria. We combined current depression/anxiety, and those who had ever been diagnosed as one category, and those who are not currently experiencing depression or anxiety, for this comparison.

Explanatory Variable (BMI): Obesity was measured using ~~Body Mass Index (~~BMI~~)~~ based on parent-reported height and weight, categorized according to CDC guidelines (CDC, 2016) into four groups by age-adjusted percentile: Underweight (less than 5th percentile), Normal weight (5th to 84th percentile), Overweight (85th to 94th percentile), and Obese (95th percentile or above)

Covariates: Covariates included the child's age, sex, race/ethnicity, and federal poverty levels (FPL), which represent the socioeconomic status of the family. The question on the FPL is: "What is the income level (federal poverty level, FPL) of the household that this child lives in? The responses are divided into 0-99% FPL; 100-199% FPL; 200-399% FPL; 400% FPL or greater.

Moderators: These include interpersonal factors that reflect social relationships and peer interactions.

Bullying was assessed by asking respondents to report how often the child experienced bullying in the past 12 months. For the purpose of analysis, the responses were categorized into three categories: levels: "Never in past 12 months"= Never; "1-2 times in past 12 months" and "1-2 times per month," into Occasional and "1-2 times per week" and "Almost every day" into Frequent. This single-item bullying measure has been used in all NSCH cycles and aligns with CDC Youth Risk Behavior Surveillance (YRBS) definitions of bullying. Prior NSCH

documentation confirms the content validity and reliability of this item for population monitoring.

Making or keeping friends involved respondents, indicating the child's level of difficulty in forming and maintaining friendships. The responses provided for this evaluation included "No difficulty," "A little difficulty," and "A lot of difficulty." This indicator originates from the Strengths and Difficulties Questionnaire (SDQ) peer problems subscale, which has demonstrated construct validity and good internal consistency (α ≈ 0.70) in U.S. samples.

Father's/Mother's mental health was evaluated by asking respondents to assess the mental health status of the father/mother, if he/she was the primary caregiver. The assessment options included "Excellent/very good", "Good", and "Fair/poor." This global self-rated item has been validated against standardized screening tools, such as the PHQ-2 and K6, showing a moderate to strong correlation in U.S. population surveys.

Family resilience was evaluated by respondents who assessed the family's ability to cope during difficult times. The question asked was: Does this child live in a home where the family demonstrates qualities of resilience during difficult times? The options included "All or most of the time to 0-1 items", "All or most of the time to 2-3 items", "All or most of the time to all 4 items." These items comprise the NSCH Family Resilience Scale, which demonstrates strong internal consistency (Cronbach's α = 0.80) and established construct validity.

All moderator items are part of the validated NSCH item bank maintained by the Child and Adolescent Health Measurement Initiative (CAHMI). Psychometric

Formatted: Font: (Default) Arial

Formatted: Font: (Default) Arial

Formatted: Font: (Default) Arial

Formatted: Font: (Default) Arial

Formatted: Font: (Default) Arial

Formatted: Font: (Default) Arial

properties of these scales have been described in prior technical documentation and peer-reviewed analyses (53,54).

A non-response bias analysis by NSCH showed no signs of bias. We applied weights, stratification, and accounted for clustering to enhance generalizability to the U.S. population of children and provided an unbiased test of statistical significance. The analytic sample included only children with complete responses on BMI, depression, and anxiety questions. Children under 6 years were excluded to avoid sensitivities related to BMI calculations and varying developmental stages. Of the original 64,535 children aged 6–17 years, we excluded 23,715 cases with non-analytic codes (95 were less than 2 or 3 or 5 years old; 99 were missing or refused) for essential study variables. The ultimate analytic sample was comprised of 40,820 children who had valid information on all variables of interest. We compared the excluded and retained participants in terms of age, sex, race/ethnicity, and family income (Table S1); the distributions were broadly comparable. An inspection of the missing data pattern revealed no residual missingness and no need for multiple imputation.

Formatted: Font: (Default) Arial

Formatted: Font: (Default) Arial

Formatted: Font: (Default) Arial

Formatted: Font: (Default) Arial

Formatted: Font: (Default) Arial

IRB and Ethics Statement:

These survey results are openly available and de-identified; therefore, secondary analysis of the results is, according to federal guidelines, exempt from consideration by an Institutional Review Board (IRB).

Analysis:

The analysis used the R program and survey-weighted statistical procedures to address the NSCH's complex sampling design, correcting for oversampling, non-response, and stratification issues. The NSCH data included weights that adjust for unequal selection probabilities, non-response, and post-stratification to accurately represent the population, as per the NSCH survey codebook (49).

The descriptive analysis includes both unweighted and weighted frequencies and percentages across demographic subgroups. A bivariate analysis examined relationships between BMI categories and LDD and LDA, as well as interpersonal variables. Design-based chi-square tests identified statistically significant associations. LDD or LDA were analyzed across demographic and interpersonal variable categories, as well as BMI categories, for statistical associations. Survey-weighted logistic regression models were used to evaluate the association between BMI and LDD & LDA, controlling for covariates and testing interactions with interpersonal variables. Adjusted odds ratios (aOR) and 95% confidence intervals (95%CI) are reported, with statistical significance set at $p < 0.05$. Survey design variables, including weights, strata, and primary sampling units, were incorporated using the svyglm() function in R, with models defined using a quasibinomial family to address overdispersion in binary outcomes. Given mild overdispersion in the binary outcomes, we used quasibinomial logistic models to obtain robust variance estimates. Multicollinearity was assessed using variance inflation factors (VIF < 2 for all variables). All moderator–BMI interactions were pre-specified based on the socio-ecological framework. Stratified interaction analyses were conducted as exploratory and hypothesis-generating. We did not apply formal corrections for multiple comparisons; therefore, these results should be interpreted with caution regarding type I error risk.

Formatted: Font: (Default) Arial

Formatted: Font: (Default) Arial

RESULTS

In this study, the sample (Table 1), represented approximately 29 million children in the United States. The sample was evenly distributed among children aged 6-11 (49.3%), and those aged 12-17 years (50.7%). Similarly, the sample was balanced between male (50.9%) and female (49.0%) participants. While 57.3% were non-Hispanic White, 7.5% were ~~Non~~non-Hispanic Black, and 11.6% reported other/multiracial. Furthermore, it was found that 60.5% of the children had a normal BMI, while 15.1% were classified as overweight, and 15.3% obese. An LDA was reported in 13.3% of the participants, whereas an LDD occurred in 4.5%. In terms of interpersonal variables, 63.8% of children reported never being bullied within a year. For kids who were bullied, 26.6% were bullied once or twice within the past year, with 5.7% bullied once or twice per month, and lower percentages bullied several times (2.7% per week, 1.1% per day). Additionally, 4.2% of the children indicated difficulties in making friends. It is also noteworthy that 4.5% and 6.0% of children's parental guardians (father/male and mother/female, respectively) exhibited poor mental health status. Lastly, only 5.2% of families demonstrated characteristics of full resilience across all four assessed items.

Table 1: Descriptive Stats (n=40820) of ~~all~~ children 6-17 years in the 2021-2022 NSCH Survey~~y 21-22~~:

Variable	Categories	Unweighted frequency	Weighted frequency (in millions) (Weighted %)
**Age**	6-11 years	19108	14.4 (49.3%)
	12-17 years	21712	14.8 (50.7%)
**Sex**	Male	21205	14.9 (50.9%)
	Female	19615	14.3 (49.1%)
**Race/Ethnicity**	Hispanic	5072	6.9 (23.5%)

	White, Non-Hispanic	28911	16.7 (57.3%)
	Black, Non-Hispanic	1445	2.2 (7.5%)
	Other/Multiracial, non-Hispanic	5392	3.4 (11.6%)
**FPL*Federal poverty level**	0-99%FPL	3132	3.1 (10.6%)
	100-199%FPL	5161	4.7 (16.2%)
	200-399%FPL	12206	9.0 (31.0%)
	400% FPL or greater	20321	12.3 (42.1%)
**BMI**	UW	3613	2.7 (9.1%)
	Normal	25952	17.7 (60.5%)
	Overweight	5806	4.4 (15.1%)
	Obese	5449	4.5 (15.3%)
**Anxiety**	No anxiety	34173	25.3 (86.7%)
	LDAifetime diagnosis of anxiety	6647	3.9 (13.3%)
**Depression**	No Depression	38434	27.9 (95.5%)
	LDDifetime diagnosis of Depression	2386	1.3 (4.5%)
**INTERPERSONAL FACTORS**			
**Bullying**	Never in past 12 months	24129	18.6 (63.8%)
	1-2 times in past 12 months	11959	7.8 (26.6%)
	1-2 times per month	2874	1.7 (5.7%)
	1-2 times per week	1281	0.8 (2.7%)
	Almost everyday	577	0.3 (1.1%)
**Making or Keeping friends**	No difficulty	30212	22.3 (76.4%)
	A little Difficulty	8532	5.7 (19.4%)
	A lot of Difficulty	2076	1.2 (4.2%)
**Mental Health of Father**	Excellent or Very Gooc	30420	22.0 (75.4%)
	Good	8405	5.9 (20.1%)
	Fair or Poor	1995	1.3 (4.5%)
**Mental Health of Mother**	Excellent or Very Gooc	29043	21.0 (71.8%)
	Good	9368	6.5 (22.2%)
	Fair or Poor	2409	1.7 (6.0%)
**Family Resilience**	All or most of time to all 4 items	1899	1.5 (5.2%)
	All or most of time to 2-3 items	3791	2.7 (9.2%)
	All of most of time to all 0-1 items	35130	25.0 (85.6%)

- FPL=Federal Poverty level

- Overall, the descriptive characteristics show that about one in seven children were classified as obese, with significant variability across interpersonal factors like bullying exposure and parental mental health, which provides the context for subsequent analyses.

Formatted: Bulleted + Level: 1 + Aligned at: 0.25" + Indent at: 0.5"

~~Table S1 – Comparison of Sample Characteristics Before and After Exclusions~~

~~VARIABLE~~	~~CATEGORY~~	~~BEFORE EXCLUSION (N≈ 64,535)~~	~~AFTER EXCLUSION (N ≈ 40,820)~~
~~AGE (YEARS)~~	~~Mean ± SD~~	~~11.87 ± 3.54~~	~~11.78 ± 3.55~~
~~SEX~~	~~Male~~	~~33,569 (51.9%)~~	~~21,205 (52.0%)~~
	~~Female~~	~~30,966 (48.1%)~~	~~19,615 (48.0%)~~
~~RACE / ETHNICITY~~	~~White~~	~~9,521 (14.8%)~~	~~5,072 (12.4%)~~
	~~Hispanic~~	~~41,709 (64.6%)~~	~~28,911 (70.9%)~~
	~~Black~~	~~4,382 (6.8%)~~	~~1,445 (3.5%)~~
	~~Other / Multiracial~~	~~8,923 (13.8%)~~	~~5,392 (13.2%)~~
~~FEDERAL POVERTY LEVEL (FPL)~~	~~< 100%~~	~~8,512 (13.2%)~~	~~3,132 (7.7%)~~
	~~100–199%~~	~~10,645 (16.5%)~~	~~5,161 (12.6%)~~
	~~200–399%~~	~~19,108 (29.6%)~~	~~12,206 (29.9%)~~
	~~≥ 400%~~	~~26,270 (40.7%)~~	~~20,321 (49.8%)~~

Formatted: Font: (Default) Arial

Formatted: Font: (Default) Arial

Formatted: Font: (Default) Arial

Formatted: Font: (Default) Arial

Formatted: Font: (Default) Arial

Formatted: Font: (Default) Arial

Table S1 – Comparison of Sample Characteristics Before and After Exclusions

VARIABLE	CATEGORY	BEFORE EXCLUSION (N≈ 64,535)	AFTER EXCLUSION (N ≈ 40,820)
AGE (YEARS)	Mean ± SD	11.87 ± 3.54	11.78 ± 3.55

Formatted: Font: (Default) Arial

Formatted: Font: (Default) Arial

SEX	Male	33,569 (51.9%)	21,205 (52.0%)
	Female	30,966 (48.1%)	19,615 (48.0%)
RACE / ETHNICITY	White	9,521 (14.8%)	5,072 (12.4%)
	Hispanic	41,709 (64.6%)	28,911 (70.9%)
	Black	4,382 (6.8%)	1,445 (3.5%)
	Other / Multiracial	8,923 (13.8%)	5,392 (13.2%)
FEDERAL POVERTY LEVEL (FPL)	< 100%	8,512 (13.2%)	3,132 (7.7%)
	100–199%	10,645 (16.5%)	5,161 (12.6%)
	200–399%	19,108 (29.6%)	12,206 (29.9%)
	≥ 400%	26,270 (40.7%)	20,321 (49.8%)

Formatted: Font: (Default) Arial

Formatted: Font: (Default) Arial

Formatted: Font: (Default) Arial

Formatted: Font: (Default) Arial

The above supplementary table (Table S1) demonstrates that there were no significant demographic differences between participants who were included and those who were excluded.

The analysis in Table 2 demonstrates a significant association between obesity and the occurrence of LDD and LDA among children aged 6 to 17 years. In the initial model, obese children exhibited 1.60 times higher odds of experiencing LDD compared to those with a normal BMI (OR= 1.60; 95% CI: 1.27-2.02). Furthermore, after adjusting for age, race, sex, and FPL, this association intensified, revealing that

obese children had 2.08 times higher odds of experiencing depression (aOR 2.08; 95%CI: 1.64-2.64). It was also observed that there exists a dose-response relationship, whereby overweight and obese children present progressively greater odds of depression than their normal weight counterparts.

Table 2: Multivariate Logistic Regression for BMI and LDD/LDA.

Variable	BMI Category	Depression		Anxiety	
		Unadjusted	Adjusted*	Unadjusted	Adjusted**
		OR (95% CI)	OR (95% CI)	OR (95% CI)	OR (95% CI)
	Normal (reference	1.00	1.00	1.00	1.00
	UW	0.63(0.46-0.87)	0.91(0.65-1.27)	0.85(0.70-1.02	1.03(0.85-1.24)
	Overweight	1.41(1.07-1.84)	1.53(1.18-197)	0.92 (0.80-1.07	1.01 (0.87-1.18)
	Obese	1.60(1.27-2.02)	2.08(1.64-2.64)	1.20(1.01-1.43	1.48(1.25-1.76)

Adjusted*	Adjusted for age, sex, race and FPL levels	Adjusted**	Adjusted for age, sex, and race

BMI Category	LDD (Unadjusted OR, 95% CI)	LDD (Adjusted¹ OR, 95% CI)	LDA (Unadjusted OR, 95% CI)	LDA (Adjusted² OR, 95% CI)
Normal (reference)	1.00	1.00	1.00	1.00
Underweight	0.63 (0.46–0.87)	0.91 (0.65–1.27)	0.85 (0.70–1.02)	1.03 (0.85–1.24)
Overweight	1.41 (1.07–1.84)	1.53 (1.18–1.97)	0.92 (0.80–1.07)	1.01 (0.87–1.18)
Obese	1.60 (1.27–2.02)	2.08 (1.64–2.64)	1.20 (1.01–1.43)	1.48 (1.25–1.76)

[1] Adjusted for age, sex, race/ethnicity, and federal poverty level (FPL).
[2] Adjusted for age, sex, and race/ethnicity.

These results demonstrate a clearly that higher BMI is associated with increased odds of LDD and LDA after adjustment for demographic factors.

With anxiety, obese children had 1.20 times greater odds of LDA than normal weight children in the unadjusted analyses (OR=1.20; 95% CI: 1.01-1.43). After adjustment

Formatted Table

Formatted: Font: (Default) Arial

Formatted: Font: (Default) Arial

Formatted: Font: (Default) Arial

Formatted: Font: (Default) Arial

Formatted: Font: (Default) Arial

Formatted: Font: (Default) Arial

Formatted: Normal, Right: 0", Space Before: Auto, After: Auto, Line spacing: single

Formatted: Font: (Default) Arial

Formatted: Font: (Default) Arial

Formatted: Font: (Default) Arial

Formatted: Font: (Default) Arial

Formatted: Font color: Auto

for age, sex and race/ethnicity, obese children had also significantly greater odds of LDA than their normal weight counterparts, having 1.48 times greater odds of anxiety (aOR 1.48; 95% CI: -1.25-1.76).

Stratified analysis (Table 3.1, 3.2 and 3.3) showed that the link between obesity and depression shifts depended on key social factors, especially bullying and the mental health of parents. Among children who were never bullied, those with obesity were more than twice as likely to experience LDD compared to their normal weight peers, when adjusted for age, sex, race/ethnicity and FPL (aOR 2.12; 95% CI: :-1.45–3.11). This association also remained significant for children who were bullied occasionally (aOR 1.65; 95% CI: 1.14–2.39), though among children who were bullied frequently, the confidence interval for the obesity–depression association crossed the null (aOR 1.35; 95% CI: 0.87–2.08), indicating limited statistical evidence for an association in this subgroup.it was not statistically significant for those who were bullied frequently (aOR 1.35; 95% CI: 0.87–2.08). A similar trend was seen when looking at maternal mental health. Among children whose mothers had excellent or very good mental health, those with obesity had a 28% higher odds of being LDD (aOR 1.28; 95% CI: 1.02–1.62), while in children whose mothers reported poor mental health, the odds jumped to 70% higher (aOR 1.70; 95% CI: 1.07–2.70) as compared to normal weight children. The pattern was even more pronounced when looking at fathers: children with obesity had significantly higher odds of LDD whether their fathers reported excellent (aOR 1.54; 95% CI: 1.17–2.02), good (aOR 2.09; 95% CI: 1.35–3.22), or poor mental health (aOR 2.54; 95% CI: 1.51–4.29).

Similarly, Figures 1, 2, and 3 presented indicate that children with a history of frequent bullying exhibited significantly greater predicted probabilities of depression across all BMI ranges, particularly among children who were overweight or obese.

Formatted: Font: (Default) Arial

Furthermore, children whose parents, both mothers and fathers, reported poor mental health demonstrated a significantly increased risk of depression, with a clear gradient observed from excellent to poor parental mental health. These findings corroborate the interactive roles of interpersonal exposures—namely, bullying and parental psychological well-being—in modifying the association between body weight and mental health outcomes among children.

Table 3: Effect of the Interpersonal Factors on the Unadjusted and aAdjusted Odds of Lifetime Diagnosis of Depression (LDD) among Children 6-17 years with Obesity

Table 3.1: Adjusted Odds Ratios (aOR) for LDD by BMI Category, Stratified by Mother's Mental Health

Mother's Mental Health	BMI Category	aOR¹	95% CI	p-value
Excellent / Very Good	Underweight	1.04	0.80–1.35	0.766
	Overweight	0.94	0.77–1.14	0.516
	Obese	1.28	1.02–1.62	**0.035**
Good	Underweight	1.00	0.72–1.39	0.993
	Overweight	0.97	0.72–1.29	0.815
	Obese	1.34	0.99–1.82	0.061
Fair / Poor	Underweight	0.88	0.44–1.74	0.704
	Overweight	1.16	0.75–1.79	0.503
	Obese	1.70	1.07–2.70	**0.025**

¹ Adjusted for child age, sex, race/ethnicity, and federal poverty level (FPL).

Mother's Mental Health	BMI Category	aOR¹	95% CI	p-value
**Excellent/ Very Good**	Underweight	1.04	0.80 – 1.35	0.766
	Overweight	0.94	0.77 – 1.14	0.516
	Obese	1.28	1.02 – 1.62	**0.035**
**Good**	Underweight	1.00	0.72 – 1.39	0.993
	Overweight	0.97	0.72 – 1.29	0.815
	Obese	1.34	0.99 – 1.82	0.061
**Fair/Poor**	Underweight	0.88	0.44 – 1.74	0.704
	Overweight	1.16	0.75 – 1.79	0.503
	Obese	1.70	1.07 – 2.70	**0.025**

Table 3.2: Adjusted Odds Ratios (aOR) for LDD by BMI Category, Stratified by Father's Mental Health

Formatted: Font: (Default) Arial
Formatted: Font: (Default) Arial
Formatted: Centered
Formatted: Font: (Default) Arial
Formatted: Centered
Formatted: Font: (Default) Arial
Formatted: Centered
Formatted: Font: (Default) Arial
Formatted: Centered
Formatted: Font: (Default) Arial
Formatted: Centered
Formatted: Font: (Default) Arial
Formatted: Centered
Formatted: Font: (Default) Arial
Formatted: Centered
Formatted: Font: (Default) Arial
Formatted: Centered
Formatted: Font: (Default) Arial
Formatted: Centered
Formatted: Left
Formatted: Font: (Default) Arial

Father's Mental Health	BMI Category	aOR[1]	95% CI	p-value
Excellent / Very Good	Underweight	0.96	0.62–1.49	0.862
	Overweight	1.36	1.02–1.82	0.037
	Obese	1.54	1.17–2.02	0.002
Good	Underweight	0.82	0.42–1.57	0.544
	Overweight	1.63	0.99–2.68	0.052
	Obese	2.09	1.35–3.22	0.001
Fair / Poor	Underweight	0.62	0.28–1.35	0.227
	Overweight	1.20	0.61–2.35	0.599
	Obese	2.54	1.51–4.29	0.001
**Father's Mental Health**	**BMI Category**	**aOR***	**95% CI**	**p-value**

[1] Adjusted for child age, sex, race/ethnicity, and federal poverty level (FPL).

Table 3.3: Adjusted Odds Ratios (aOR) for LDD by BMI Category, Stratified by Bullying Frequency

**Bullying Frequency**	**BMI Category**	**aOR***	**95% CI**	**p-value**				
**Never**	Underweight	1.09	0.59–2.03	0.781				
	Overweight	1.07	0.73–1.56	0.745				
	Obese	2.12	1.45–3.11	**0.000**	**BMI Category**	**aOR***	**95% CI**	**p-value**
**Occasional**	Underweight	0.80	0.53–1.21	0.292				
	Overweight	1.77	1.24–2.52	**0.002**				

	Obese	1.65	1.14 – 2.39	0.008
Frequent	Underweight	0.69	0.32 – 1.45	0.324
	Overweight	1.51	0.81 – 2.81	0.194
	Obese	1.35	0.87 – 2.08	0.176
Bullying Frequency				
Never (data$bullied_2122 == 1 ~ "Never")	Underweight	1.09	0.59 – 2.03	0.781
	Overweight	1.07	0.73 – 1.56	0.745
	Obese	2.12	1.45 – 3.11	**0.000**
Occasional (data$bullied_2122 %in% c(2, 3) ~ "Occasional")	Underweight	0.80	0.53 – 1.21	0.292
	Overweight	1.77	1.24 – 2.52	**0.002**
	Obese	1.65	1.14 –	**0.008**

Formatted: Font: 12 pt

			2.39	
Frequent (data$bullied_2122 %in% c(4, 5) ~ "Frequent")	Underweight	0.69	0.32 – 1.45	0.324
	Overweight	1.51	0.81 – 2.81	0.194
	Obese	1.35	0.87 – 2.08	0.176

Bullying Frequency	BMI Category	aOR¹	95% CI	p-value
Never	Underweight	1.09	0.59–2.03	0.781
	Overweight	1.07	0.73–1.56	0.745
	Obese	2.12	1.45–3.11	**<0.001**
Occasional	Underweight	0.80	0.53–1.21	0.292
	Overweight	1.77	1.24–2.52	**0.002**
	Obese	1.65	1.14–2.39	**0.008**
Frequent	Underweight	0.69	0.32–1.45	0.324
	Overweight	1.51	0.81–2.81	0.194
	Obese	1.35	0.87–2.08	0.176

¹ Adjusted for child age, sex, race/ethnicity, and federal poverty level (FPL).

Figure 1; Predicted Probability of Depression - Bullying Category

Figure 2: Predicted Probability of Depression- Mental Health of Father

Figure 3: Predicted Probability of Depression- Mental Health of Mother

Together, these stratified results suggest that the association between obesity and depression varies depending on the child's bullying experiences and parental mental health, highlighting the moderating role of interpersonal environments.

In contrast, **difficulty making friends** and **family resilience (Table 3.4)** did not interact with obesity, but both were strong correlates of depression on their own.

Formatted: Font: (Default) Arial
Formatted Table
Formatted: Font: (Default) Arial
Formatted: Centered
Formatted: Font: (Default) Arial
Formatted: Centered
Formatted: Font: (Default) Arial
Formatted: Centered
Formatted: Font: (Default) Arial
Formatted: Centered
Formatted: Font: (Default) Arial
Formatted: Centered
Formatted: Font: (Default) Arial
Formatted: Centered
Formatted: Font: (Default) Arial
Formatted: Centered
Formatted: Font: (Default) Arial
Formatted: Centered
Formatted: Font: (Default) Arial
Formatted: Centered
Formatted: Not Highlight
Formatted: Font: (Default) Arial
Formatted: Font: (Default) Arial

Children who had a little difficulty making friends had five times higher odds to report LDD (aOR 4.96; 95% CI: 4.07–6.05), and those with a lot of difficulty had almost thirteen times the odds (aOR 12.94; 95% CI: 10.03–16.44), compared to children who reported no difficulty. On the flip side, strong family resilience seemed to offer protection. Children from families that were resilient to “most items” had 43% lower odds of LDD (aOR 0.57; 95% CI: 0.42–0.77), and those from families resilient to “some items” had 60% lower odds (aOR 0.40; 95% CI: 0.31–0.53) compared to families resilient to all items.

Table 3.4: Adjusted Odds Ratio for LDD by Difficulty in Making Friends and Family Resilience

Variable	Category	aOR[1]	95% CI
**Difficulty Making Friends**	No difficulty (reference)	1.00	1.00
	A little difficulty	4.96	4.07–6.05
	A lot of difficulty	12.94	10.03–16.44
**Family Resilience**	Resilient to all items (reference)	1.00	1.00
	To most items	0.57	0.42–0.77
	To some items	0.40	0.31–0.53

[1] Adjusted for child age, sex, race/ethnicity, and federal poverty level (FPL).

Variable		aOR*	95% CI
~~**Make friend**~~	~~**No Difficulty-Reference**~~	~~1.00~~	~~1.00~~
	~~**A little difficulty**~~	~~4.96~~	~~4.07-6.05~~
	~~**A lot of difficulty**~~	~~12.94~~	~~10.03-16.44~~
~~**Family Resilience**~~	~~**Resilient to all-Reference**~~	~~1.00~~	~~1.00~~
	~~**To most items**~~	~~0.57~~	~~0.42-0.77~~
	~~**To some items**~~	~~0.40~~	~~0.31-0.53~~

~~Adjusted* for age, sex, race/ethnicity and FPL.~~

While making friends and family resilience did not interact with BMI, both remained independently associated with depression, suggesting additional pathways of psychosocial risk and buffering.

Formatted: Font: (Default) Arial
Formatted Table
Formatted: Font: (Default) Arial
Formatted: Font: (Default) Arial
Formatted: Font: (Default) Arial
Formatted: Font: (Default) Arial
Formatted: Font: (Default) Arial
Formatted: Font: (Default) Arial
Formatted: Font: Not Bold
Formatted: Left
Formatted: Font: Not Bold
Formatted: Font: (Default) Arial

When examining the relationship of **body weight and social challenges** related to **LDA in children,** the impact of obesity appeared to vary depending on the child's ability to make friends, and the mental health of their father. Table 4.1 shows that among children who reported **no difficulty making friends,** obesity was not significantly linked to anxiety when adjusted for age, sex, race/ethnicity and FPL (aOR 1.12; 95% CI: 0.87–1.44). The same pattern held for those with **a little difficulty,** where the odds of anxiety were slightly higher for children with obesity, but this was not statistically significant (aOR 1.27; 95% CI: 0.94–1.72). However, among children who had **a lot of difficulty making friends,** the findings were different: children with obesity had twice the odds of experiencing anxiety compared to their normal-weight peers (aOR 1.91; 95% CI: 1.13–3.22).

Table 4: Effect of the Interpersonal Factors on the ~~Unadjusted and Adjusted Odds of Lifetime Diagnosis of Anxiety(~~LDA~~)~~ among Children 6-17 years with Obesity

Table 4.1: Adjusted Odds Ratios (aOR) for LDA by BMI Category, Stratified by Difficulty in Making Friends

Difficulty in Making Friends	BMI Category	aOR¹	95% CI	p-value
**No difficulty**	Underweight	1.05	0.80–1.39	0.724
	Overweight	1.06	0.84–1.33	0.642
	Obese	1.12	0.87–1.44	0.368
**Little difficulty**	Underweight	0.75	0.52–1.08	0.125
	Overweight	0.79	0.61–1.03	0.081
	Obese	1.27	0.94–1.72	0.121
**A lot of difficulty**	Underweight	1.34	0.79–2.28	0.277
	Overweight	1.38	0.89–2.14	0.146
	Obese	1.91	1.13–3.22	**0.015**

~~Difficulty in Making Friends~~	~~BMI Category~~	~~aOR*~~	~~95% CI~~	~~p-value~~
~~**No difficulty**~~	~~Underweight~~	~~1.05~~	~~0.80 – 1.39~~	~~0.724~~
	~~Overweight~~	~~1.06~~	~~0.84 – 1.33~~	~~0.642~~

Formatted: Font: (Default) Arial
Formatted Table
Formatted: Font: (Default) Arial
Formatted: Centered
Formatted: Font: (Default) Arial
Formatted: Centered
Formatted: Font: (Default) Arial
Formatted: Centered
Formatted: Font: (Default) Arial
Formatted: Centered
Formatted: Font: (Default) Arial
Formatted: Centered
Formatted: Font: (Default) Arial
Formatted: Centered
Formatted: Font: (Default) Arial
Formatted: Centered
Formatted: Font: (Default) Arial
Formatted: Centered
Formatted: Font: (Default) Arial
Formatted: Centered
Formatted: Font: Not Bold
Formatted: Left

	~~Obese~~	~~1.12~~	~~0.87 – 1.44~~	~~0.368~~
~~**Little Difficulty**~~	~~Underweight~~	~~0.75~~	~~0.52 – 1.08~~	~~0.125~~
	~~Overweight~~	~~0.79~~	~~0.61 – 1.03~~	~~0.081~~
	~~Obese~~	~~1.27~~	~~0.94 – 1.72~~	~~0.121~~
~~**A Lot of Difficulty**~~	~~Underweight~~	~~1.34~~	~~0.79 – 2.28~~	~~0.277~~
	~~Overweight~~	~~1.38~~	~~0.89 – 2.14~~	~~0.146~~
	~~**Obese**~~	~~**1.91**~~	~~**1.13 – 3.22**~~	~~**0.015**~~

[1] Adjusted for child age, sex, race/ethnicity, and federal poverty level (FPL).

Table 4.2: Adjusted Odds Ratios (aOR) for LDA~~Anxiety~~ by BMI Category, Stratified by Father Mental Health

Father's Mental Health	BMI Category	aOR[1]	95% CI	p-value
**Excellent / Very Good**	Underweight	1.07	0.85–1.36	0.552
	Overweight	1.00	0.82–1.22	0.983
	Obese	1.19	0.95–1.48	0.126
**Good**	Underweight	0.92	0.63–1.35	0.666
	Overweight	0.78	0.59–1.04	0.089
	Obese	1.56	1.14–2.14	**0.006**
**Fair / Poor**	Underweight	1.21	0.65–2.24	0.549
	Overweight	1.55	0.95–2.54	0.078
	Obese	2.14	1.34–3.41	**0.001**

[1] Adjusted for child age, sex, race/ethnicity, and federal poverty level (FPL).

~~Father's Mental Health~~	~~BMI Category~~	~~aOR*~~	~~95% CI~~	~~p-value~~
~~**Excellent/Very Good**~~	~~Underweight~~	~~1.07~~	~~0.85 – 1.36~~	~~0.552~~
	~~Overweight~~	~~1.00~~	~~0.82 – 1.22~~	~~0.983~~
	~~Obese~~	~~1.19~~	~~0.95 – 1.48~~	~~0.126~~
~~**Good**~~	~~Underweight~~	~~0.92~~	~~0.63 – 1.35~~	~~0.666~~
	~~Overweight~~	~~0.78~~	~~0.59 – 1.04~~	~~0.089~~
	~~**Obese**~~	~~**1.56**~~	~~**1.14 – 2.14**~~	~~**0.006**~~
~~**Fair/Poor**~~	~~Underweight~~	~~1.21~~	~~0.65 – 2.24~~	~~0.549~~
	~~Overweight~~	~~1.55~~	~~0.95 – 2.54~~	~~0.078~~
	~~**Obese**~~	~~**2.14**~~	~~**1.34 – 3.41**~~	~~**0.001**~~

~~Adjusted* for age, sex, race/ethnicity and FPL.~~

A similar trend appeared when examining **fathers' mental health (Table 4.2).** For children whose fathers reported ~~had~~ **excellent or very good mental health,** obesity among obese children, the confidence interval for the obesity–anxiety association crossed the null (aOR=1.19; 95% CI: 0.95–1.48), indicating limited statistical

Formatted: Font: Not Bold
Formatted: Left
Formatted: Font: (Default) Arial
Formatted Table
Formatted: Font: (Default) Arial
Formatted: Centered
Formatted: Font: (Default) Arial
Formatted: Centered
Formatted: Font: (Default) Arial
Formatted: Centered
Formatted: Font: (Default) Arial
Formatted: Centered
Formatted: Font: (Default) Arial
Formatted: Centered
Formatted: Font: (Default) Arial
Formatted: Centered
Formatted: Font: (Default) Arial
Formatted: Centered
Formatted: Font: (Default) Arial
Formatted: Centered
Formatted: Font: Not Bold
Formatted: Left
Formatted: Font: Not Bold
Formatted: Left
Formatted Table
Formatted: Font: (Default) Arial
Formatted: Font: (Default) Arial

evidence for an association in this subgroup~~was no~~, ~~t significantly linked to anxiety~~after ~~when~~ adjusting~~ted~~ for age, sex, race/ethnicity and FPL. ~~(aOR 1.19; 95% CI: 0.95–1.48).~~ Among those whose fathers reported **good** mental health, obesity was associated with a 56% increase in odds of anxiety (aOR 1.56; 95% CI: 1.14–2.14). The most substantial effect was observed~~seen~~ in children whose fathers had **fair or poor mental health**—here, obesity more than doubled the odds of experiencing anxiety (aOR 2.14; 95% CI: 1.34–3.41).

Thus, for LDA, the association between BMI and internalizing symptoms was strongest among children who had difficulty maintaining friendships and those whose fathers reported poorer mental health.

Formatted: Font: (Default) Arial, Not Italic
Formatted: Line spacing: Double
Formatted: Font: (Default) Arial, Not Italic
Formatted: Font: (Default) Arial, Not Italic
Formatted: Font: (Default) Arial

Figures 4 and 5 show that the likelihood of anxiety was notably higher in children with obesity when their father reported poor or fair mental health, exceeding 25%. Additionally, children who faced more challenges in forming friendships had a significantly higher risk of LDA~~anxiety~~ across all BMI categories, especially among those with obesity. These results indicate that paternal mental health and social integration play a crucial role in modifying the association between BMI and LDA~~anxiety~~ in children.

Figure 4 : Predicted Probability of LDA~~Anxiety~~ – Making Friends

Figure 5: Predicted Probability of LDA~~Anxiety~~ – Father's Mental Health

Discussion:

TOur findings are consistent with existing literature on the association between childhood obesity and severe mental health difficulties and reinforce the importance of recognizing obesity as an essential risk factor for depression and anxiety in childhood populations.his study provides strong evidence that obesity is significantly associated with both depression and anxiety, even after adjusting for age, sex, race/ethnicity, and poverty levels. This finding is consistent with the existing literature wherein our study reinforces the severe mental health difficulties experienced by obese childrenIt, and indicates how it is imperative to recognize obesity as an essential risk factor for depression and anxiety in childhood populations (9,47,55). However, other longitudinal investigations have reported weaker or non-significant associations once baseline emotional symptoms or socioeconomic stressors are rigorously controlled, suggesting that obesity may not uniformly predict mental illness across all contexts (11,12). This research adds contributes to the literature by revealing that interpersonal stressors exacerbate these risks, aligning with socio-ecological and diathesis-stress models. We observed heterogeneity across BMI groups, particularly between the pathways of depression and anxiety. While the literature corroborates this association for both depression and anxiety, our study has identified a nuance: BMI exhibits a stronger association with depression than with anxiety.

Although the literature recognizes bullying as a risk factor for depression and anxiety (56), especially demonstrating a dose–response relationship where more frequent victimization predicts greater internalizing risk (20,22,57), another paradox identified in this study was that infrequent bullying was more predictive of depression than frequent bullying. This interaction was absent in the development of anxiety in children. Interestingly, the link between obesity and depression was most noticeable

Formatted: Font: (Default) Arial

Formatted: Normal

Formatted: Font: (Default) Arial

Formatted: Font: (Default) Arial

Formatted: Font: (Default) Arial

Formatted: Font: (Default) Arial

Formatted: Font: (Default) Arial

Formatted: Font: (Default) Arial

among children who faced *occasional* bullying rather than frequent bullying. Since the study is cross-sectional, we can't explain why this pattern occurs. One possible explanation, backed by resilience research, is that ongoing or repeated adversity might trigger coping mechanisms over time, whereas infrequent or unresolved bullying could be more psychologically destabilizing.~~,~~ ~~The literature does not address this paradox, and~~ W~~w~~e can hypothesize that these findings, ~~while~~although counterintuitive, may ~~result~~be ~~from~~due to social desirability bias or selection bias. We can assume that parents have underreported the frequency of bullying, as it engenders stigma or exposes vulnerabilities within the family, particularly when the child conceals the victimization. Nonetheless, this interpretation is speculative and requires testing in future longitudinal studies. ~~Another plausible consideration is that children who experience bullying more frequently develop coping strategies, thereby becoming more resilient to the effects of bullying. Therefore, our results indicate that the cumulative impact of infrequent but "unresolved" bullying is greater or more detrimental for children than the frequent bullying that schools or parents actively address.~~ Consequently, we believe that our findings suggest the necessity for screening mental health issues, even amidst seemingly minor or infrequent peer conflicts.

~~The literature has been routine in positing~~Previous literature posited that maternal and paternal mental health have significant effects upon children's emotional regulation, attachment style, and behavior development (31,34,35)~~(31,34,35)~~. A few studies have reported weaker associations after adjusting for socioeconomic stressors, and found that genetic heritability may partially account for these relationships—indicating parental health effects are not uniformly environmental (30,33). The literature supported this phenomenon was more

Formatted: Font: (Default) Arial

Formatted: Font: (Default) Arial

Formatted: Font: (Default) Arial

Formatted: Font: (Default) Arial

Formatted: Font: (Default) Arial

Formatted: Font: (Default) Arial

commonly observed in mother-child relationships than in father-child relationships.~~that mother-child relationships affect mental health issues more than father-child relationships.~~ The strong link between poor maternal mental health and child depression in our results likely reflects the enduring influence of traditional caregiving roles. As primary caregivers, mothers often provide most of the emotional, educational, and health-related support. Depressed or anxious mothers may be less responsive, nurturing, or emotionally attuned to their children and therefore contribute to poorer caregiving quality, which then indirectly affects the psychosocial development of children, but this would be outside the scope of our research. Mothers face an outsize share of the spadework when it comes to scheduling, cares and comforting as a career so often, they are still very much the hub around which the child's social and emotional world whirls.

Despite other interpersonal stresses, resilience appears to be a buffer against depression (58,59). Studies reported inconsistent or limited moderation effects, showing that resilience does not uniformly protect against anxiety or chronic adversity (15,39). ~~Our~~ Our ffindings indicate that, while family resilience is a powerful buffer against depression in children themselves classified as obese, it is not a powerful buffer against anxiety. The distinction may be traced back to the different cognitive and affective mechanisms underpinning these mental health issues. Depression is characterized by a negative schema of hopelessness and self-blame, which may improve more with cohesive family processes. In contrast, anxiety—associated with hyperarousal and vigilance—may necessitate non-family-centred interventions.

Formatted: Font: (Default) Arial

Formatted: Font: (Default) Arial

The importance of multi-level approaches that consider interpersonal context is reinforced by our findings. Paediatric screening practices could incorporate routine assessment of peer relationship difficulties and parental mental health where appropriate. School-based anti-bullying programs and caregiver-focused supports may help address some of the interpersonal environments associated with children's emotional well-being. The findings of this research implore the necessity for multi-level, cross-sectoral interventions, ones that extend beyond the scope of solely biomedical or lifestyle interventions for childhood obesity. Paediatricians and mental health professionals are important in adopting a proactive stance toward screening for depression and anxiety in addition to weight status screening, particularly in children with peer relationship difficulties, bullying history, or parental history of mental health issues. Other findings support the relevance of psychoeducational interventions directed toward parents and caregivers, since parental mental health is a determining factor that influences the emotional growth of children. Parent-focused interventions could enhance children's emotional well-being, while school-based anti-bullying and peer-bonding programs—especially targeting weight-based discrimination—maycould address key social risk factors. Policy approaches mayshould address the disproportionate burden on low-income and minority families, embedding obesity and mental health services in equity frameworks. At a policy level, the findings suggest that holistic approaches may be considered forto children's health, thataking considerinto account both physical and mental aspects. Policymakers mayshould also recognize that the mental health disparities associated with obesity differentially impact low-income and minority families, and therefore are of relevance to an equity framework that informs the process of planning and budgeting programs. While the NSCH dataset does not directly evaluate the

Formatted: Font: (Default) Arial

Formatted: Font: (Default) Arial

Formatted: Font: (Default) Arial

Formatted: Font: (Default) Arial

effectiveness of interventions, these results highlight potential domains where supportive policies could be relevant.

While providing additional insights into the associations between psychosocial determinants and childhood obesity as well as mental health, several limitations should be acknowledged. The NSCH design is cross-sectional and does not support causal inference. The reliance on parent reports of mental health symptoms and interpersonal behavior (e.g., bullying) may result in recall bias and social desirability. Additionally, given the number of stratified tests, there is an increased risk of type I error; thus, these interaction findings should be viewed as suggestive and requiring confirmation in future studies. The study was limited in its generalizability ~~by~~due to the U.S.-specific health, social, and policy context. Future research should utilize longitudinal designs to establish causal directions, involve multiple informants to increase validity, and include qualitative or mixed-method approaches to explain counterintuitive findings such as the lack of a dose–response relationship for infrequent bullying. Future research should confirm the generalizability of these associations in comparative studies across diverse cultural and structural contexts.

Formatted: Font: (Default) Arial

In sum, obesity in children is embedded within a network of interpersonal and contextual influences that differentially predict depression and anxiety. Multi-level, theory-driven interventions spanning healthcare, education, and family systems are essential to address these intertwined physical and mental health challenges.

Acknowledgements

The author sincerely thanks Dr. Madhivanan, Dr. Chen, Dr. Suh, Dr. Scott Carvajal, Dr. Karl Krupp, and Prof. Maia for their mentorship and guidance in statistics. Gratitude is also expressed to the Arizona Department of Health Services (AZDHS)

for their continuous partnership and support in developing this dissertation project. Additionally, the author values the technical infrastructure supplied by the University of Arizona, which facilitated computational and data management needs.

References

1. World Health Organization [WHO]. Obesity and overweight [Internet]. 2024 [cited 2024 Jul 2]. p. 76. Available from: https://www.who.int/news-room/fact-sheets/detail/obesity-and-overweight#:~:text=For adults%2C WHO defines overweight and obesity as,to be considered when defining overweight and obesity.
2. CDC US Government. Childhood Obesity Facts [Internet]. 2024. 2024 [cited 2025 Mar 15]. Available from: https://www.cdc.gov/obesity/childhood-obesity-facts/childhood-obesity-facts.html
3. Di Cesare M, Sorić M, Bovet P, Miranda JJ, Bhutta Z, Stevens GA, et al. The epidemiological burden of obesity in childhood: a worldwide epidemic requiring urgent action. BMC Med [Internet]. 2019;17(1):212. Available from: https://doi.org/10.1186/s12916-019-1449-8
4. Rankin J, Matthews L, Cobley S, Han A, Sanders R, Wiltshire H, et al. Psychological consequences of childhood obesity: psychiatric comorbidity and prevention. Adolesc Health Med Ther [Internet]. 2016; Available from: https://pubmed.ncbi.nlm.nih.gov/27881930
5. Choi H, Corredor-Waldron A, Currie J, Felton C. WHAT CAN TRENDS IN EMERGENCY DEPARTMENT VISITS TELL US ABOUT CHILD MENTAL HEALTH? Natl Bur Econ Res [Internet]. 2025; Available from: https://www.medicaid.gov/medicaid/benefits/early-and-periodic-screening-diagnostic-and-treatment

6. NIHCM Foundation. The State of Children's Health in the United States [Internet]. 2023. 2023 [cited 2025 Mar 15]. Available from: https://nihcm.org/publications/the-state-of-childrens-health-in-the-united-states
7. Quek Y-H, Tam WWS, Zhang MWB, Ho RCM. Exploring the association between childhood and adolescent obesity and depression: a meta-analysis. Obes Rev an Off J Int Assoc Study Obes. 2017 Jul;18(7):742–54.
8. Fan Y, Wang D, Wang W, Chen H, Lin J, Lin W, et al. Depression, anxiety, and development of obesity from childhood to young adulthood in China. J Psychosom Res. 2024 Oct;185:111867.
9. Chen Y, Zhang J, Yuan L, Hu H, Li T, Zhao Y, et al. Obesity and risk of depressive disorder in children and adolescents: A meta-analysis of observational studies. Child Care Health Dev. 2024 Mar;50(2):e13237.
10. Sutaria S, Devakumar D, Yasuda SS, Das S, Saxena S. Is obesity associated with depression in children? Systematic review and meta-analysis. Arch Dis Child. 2019;104(1):64–74.
11. Mannan MA, Mamun A Al, Doi SAR, Clavarino A. Prospective Associations between Depression and Obesity for Adolescent Males and Females- A Systematic Review and Meta-Analysis of Longitudinal Studies. PLoS One [Internet]. 2016; Available from: https://pubmed.ncbi.nlm.nih.gov/27285386
12. Marmorstein NR, Iacono WG, Legrand LN. Obesity and depression in adolescence and beyond: reciprocal risks. Int J Obes [Internet]. 2014; Available from: https://pubmed.ncbi.nlm.nih.gov/24480863
13. Luppino FS, Wit L de, Bouvy PF, Stijnen T, Cuijpers P, Penninx BWJH, et al. Overweight, Obesity, and Depression A Systematic Review and Meta-analysis of Longitudinal Studies. Arch Gen Psychiatry [Internet]. 2010; Available from:

https://pubmed.ncbi.nlm.nih.gov/20194822

14. Puhl RM, King KM. Weight discrimination and bullying. Best Pract Res Clin Endocrinol Metab. 2013 Apr;27(2):117–27.

15. Mesman E, Vreeker A, Hillegers M. Resilience and mental health in children and adolescents: An update of the recent literature and future directions. Curr Opin Psychiatry. 2021;34(6):586–92.

16. Buro AW, Salinas-Miranda A, Marshall J, Gray HL, Kirby RS. Obesity and neurodevelopmental and mental health conditions among adolescents aged 10–17 years: The National Survey of Children's Health 2017–2018. J Paediatr Child Health. 2022;58(10):1753–9.

17. Cheng S, Kaminga AC, Liu Q, Wu F, Wang Z, Wang X, et al. Association between weight status and bullying experiences among children and adolescents in schools: An updated meta-analysis. Child Abuse Negl [Internet]. 2022; Available from: https://doi.org/10.1016/j.chiabu.2022.105833

18. Blanco M, Solano S, Alcántara AI, Parks M, Román FJ, Sepúlveda AR. Psychological well-being and weight-related teasing in childhood obesity: a case-control study. Eat Weight Disord. 2020 Jun;25(3):751–9.

19. Şahın N, Kırlı U. The Relationship Between Peer Bullying and Anxiety-Depression Levels in Children With Obesity. Anatol J Psychiatry [Internet]. 2020; Available from: https://pubmed.ncbi.nlm.nih.gov/36425935

20. Pryor L, Brendgen M, Boivin M, Dubois L, Japel C, Falissard B, et al. Overweight during childhood and internalizing symptoms in early adolescence: The mediating role of peer victimization and the desire to be thinner. J Affect Disord [Internet]. 2016; Available from: https://pubmed.ncbi.nlm.nih.gov/27267292

21. Vuuren CL van, Wachter G, Veenstra R, Rijnhart JJM, Wal MF van der, Chinapaw MJM, et al. Associations between overweight and mental health problems among adolescents, and the mediating role of victimization. BMC Public Health [Internet]. 2019; Available from: https://pubmed.ncbi.nlm.nih.gov/31113424
22. Rupp K, McCoy SM. Bullying Perpetration and Victimization among Adolescents with Overweight and Obesity in a Nationally Representative Sample. Child Obes [Internet]. 2019; Available from: https://pubmed.ncbi.nlm.nih.gov/31062988
23. Kozasa S, Oiji A, Kiyota A, Sawa T, Kim S-Y. Relationship between the experience of being a bully/victim and mental health in preadolescence and adolescence: a cross-sectional study. Ann Gen Psychiatry [Internet]. 2017; Available from: https://pubmed.ncbi.nlm.nih.gov/29075310
24. Wood MA. Friendship Security and Intimacy Moderate the Stability of Anxiety During Early Adolescence. 2013.
25. Adedeji A, Otto C, Kaman A, Reiss F, Devine J, Ravens-Sieberer U. Peer Relationships and Depressive Symptoms Among Adolescents: Results From the German BELLA Study. Front Psychol. 2022;12(January).
26. Gorrese A. Peer Attachment and Youth Internalizing Problems: A Meta-Analysis. Child Care Q. 2016;
27. Zimmer-Gembeck MJ, Clear SJ, Campbell SM. Peer relationships and stress: Indirect associations of dispositional mindfulness with depression, anxiety and loneliness via ways of coping. J Adolesc. 2021;
28. Chiu K, Clark DM, Leigh E. Prospective associations between peer functioning and social anxiety in adolescents: A systematic review and meta-analysis. J

Affect Disord. 2020;

29. Lampard AM, Franckle RL, Davison KK. Maternal depression and childhood obesity: A systematic review. Prev Med (Baltim). 2014;59(1):60–7.

30. Foster BA, Weinstein K, Mojica CM, Davis MM. Parental Mental Health Associated With Child Overweight and Obesity, Examined Within Rural and Urban Settings, Stratified by Income. J Rural Heal Off J Am Rural Heal Assoc Natl Rural Heal Care Assoc. 2020 Jan;36(1):27–37.

31. Goodman SH, Rouse MH, Connell AM, Broth MR, Hall CM, Heyward D. Maternal Depression and Child Psychopathology: A Meta-Analytic Review. Clin Child Fam Psychol Rev [Internet]. 2011;14(1):1–27. Available from: https://doi.org/10.1007/s10567-010-0080-1

32. Wang L, Anderson JL, Dalton W, Wu T, Liu X, Zheng S, et al. Maternal Depressive Symptoms and the Risk of Overweight in Their Children. Matern Child Health J [Internet]. 2013; Available from: https://pubmed.ncbi.nlm.nih.gov/22833333

33. Hope S, Micali N, Deighton J, Law C. Maternal mental health at 5 years and childhood overweight or obesity at 11 years: evidence from the UK Millennium Cohort Study. Int J Obes [Internet]. 2019; Available from: https://doi.org/10.1038/s41366-018-0252-5

34. Connell AM, Goodman SH. The association between psychopathology in fathers versus mothers and children's internalizing and externalizing behavior problems: a meta-analysis. Psychol Bull. 2002 Sep;128(5):746–73.

35. Kluczniok D, Boedeker K, Hindi Attar C, Jaite C, Bierbaum A-L, Fuehrer D, et al. Emotional availability in mothers with borderline personality disorder and mothers with remitted major depression is differently associated with

psychopathology among school-aged children. J Affect Disord. 2018 Apr;231:63–73.

36. Choi J. The Moderating Role of Family Resilience in the Relationship of Bullying Victimization, Adverse Childhood Experiences, and Poverty Status to Adolescents' Internalizing Disorders. J Aggress Maltreat Trauma. 2022;

37. Uddin J, Ahmmad Z, Uddin H, Tatch A. Family resilience and protective factors promote flourishing and school engagement among US children amid developmental disorder and adverse psychosocial exposure. Sociol Spectr. 2021;

38. Zhuo R, Yu Y, Shi X. Family Resilience and Adolescent Mental Health during COVID-19: A Moderated Mediation Model. Int J Environ Res Public Health. 2022;

39. Ao L, Cheng X, An D, An Y, Yuan G. Relationship between Perceived Family Resilience, Emotional Flexibility, and Anxiety Symptoms: a Parent-Adolescent Dyadic Perspective. J Youth Adolesc. 2024;

40. Bethell C, Gombojav N, Whitaker RC. Family Resilience And Connection Promote Flourishing Among US Children, Even Amid Adversity. Health Aff. 2019;

41. Ghandour R, Sherman L, Vladutiu C, Ali M, Lynch S, Bitsko S. Prevalence and Treatment of Depression, Anxiety, and Conduct Problems in US Children. J Pediatr [Internet]. 2019;206:256–67. Available from: https://pmc.ncbi.nlm.nih.gov/articles/PMC6673640/pdf/nihms-1009660.pdf

42. Bronfenbrenner U. Mental Health and Well-being: A Socio-Ecological Model [Internet]. University of Minnesota School of Public Health. 2021. p. 1–2. Available from:

https://www.telethonkids.org.au/globalassets/media/documents/projects/sew---arts/the-social-ecological-model-2.2021.pdf
43. Golden SD, Earp JAL. Social Ecological Approaches to Individuals and Their Contexts: Twenty Years of Health Education & Behavior Health Promotion Interventions. Heal Educ Behav. 2012;39(3):364–72.
44. Belsky J, Jonassaint C, Pluess M, Stanton M, Brummett B, Williams R. Vulnerability genes or plasticity genes? Mol Psychiatry. 2009;14(8):746–54.
45. Pierson C. The Social Environment, Genetics, And Obesity: Evaluation of Diathesis-Stress and Differential Susceptibility Frameworks. 2013.
46. Avila C, Holloway AC, Hahn MK, Morrison KM, Restivo M, Anglin R, et al. An Overview of Links Between Obesity and Mental Health. Curr Obes Rep. 2015;4(3):303–10.
47. Beltrán-Garrayo L, Solar M, Blanco M, Graell M, Sepúlveda AR. Examining associations between obesity and mental health disorders from childhood to adolescence: A case-control prospective study. Psychiatry Res. 2023;326(March).
48. National Survey of Children’s Health. Request an NSCH Data Set-Data Resource Center for Child and Adolescent Health-A project of the Child and Adolescent Health Measurement Initiative [Internet]. Available from: https://www.childhealthdata.org/dataset
49. HHS. 2021-2022 National Survey of Children’s Health. 2024.
50. Cree Robyn A, Bitsko Rebecca H, Danielson Melissa L, Wanga Valentine, Holbrook Joseph, Flory Kate, et al. Surveillance of ADHD Among Children in the United States: Validity and Reliability of Parent Report of Provider Diagnosis. J Atten Disord [Internet]. 2022 Nov 3;27(2):111–23. Available from:

https://doi.org/10.1177/10870547221131979
51. Visser SN, Danielson ML, Bitsko RH, Holbrook JR. Convergent Validity of Parent-Reported ADHD Diagnosis: A Cross-Study Comparison. Ann Epidemiol. 2013;23(9):592.
52. Ebesutani C, Tottenham N, Chorpita B. The Revised Child Anxiety and Depression Scale - Parent Version: Extended Applicability and Validity for Use with Younger Youth and Children with Histories of Early-Life Caregiver Neglect. J Psychopathol Behav Assesss. 2015;37(4):705–18.
53. Blumberg SJ, Foster EB, Frasier AM, Et Al. Program and Collection Procedures Design and Operation of the National Survey of Children's Health, 2007. 2009;109.
54. US Census Bureau. 2021 National Survey of Children's Health: Source and Accuracy Statement [Internet]. 2021. Available from: https://www2.census.gov/programs-surveys/nsch/technical-documentation/source-and-accuracy/2021-NSCH-Source-and-Accuracy-Statement.pdf
55. Lindberg L, Hagman E, Danielsson P, Marcus C, Persson M. Anxiety and depression in children and adolescents with obesity: A nationwide study in Sweden. BMC Med. 2020;18(1):1–9.
56. Foody M, Samara M, O'Higgins Norman J. Bullying by siblings and peers: Poly-setting victimization and the association with problem behaviours and depression. Br J Educ Psychol. 2020 Jun;90 Suppl 1:138–57.
57. Van Vuuren CL, Wachter GG, Veenstra R, Rijnhart JJM, van der Wal MF, Chinapaw MJM, et al. Associations between overweight and mental health problems among adolescents, and the mediating role of victimization. BMC

Public Health. 2019;

58. Anderson JR, Mayes TL, Fuller A, Hughes JL, Minhajuddin A, Trivedi MH. Experiencing bullying's impact on adolescent depression and anxiety: Mediating role of adolescent resilience. J Affect Disord. 2022 Aug;310:477–83.

59. Lin L, Chien Y, Chen Y, Wu C, Chiou H. Bullying Experiences , Depression , and the Moderating Role of Resilience Among Adolescents. 2022;10(May):1–11.

Manuscript Title: Between the Scales and the Scars: The Interpersonal Burden of Body Weight on Children's Mental Health: Evidence from the 2021–22 National Survey of Children's Health (NSCH)
Manuscript ID: PGPH-D-25-02696
Authors: Fnu Kajal et al.
Journal: PLOS Global Public Health

Dear Editors and Reviewers,

We sincerely thank the reviewers for their thoughtful and constructive comments. We carefully revised the manuscript to address all the points raised. Below, we provide a detailed, point-by-point response, including specific line numbers indicating where changes were made in the revised manuscript.

Reviewer 1 Comments and Author Responses

Introduction:

The introduction does a good job of summarizing the literature, but the research gap is not clearly defined. It would be helpful to highlight that few studies have closely examined the interpersonal and social factors in the connection between obesity and mental health.
Response: We agree. We clarified the research gap by explicitly noting the absence of studies examining multiple interpersonal factors simultaneously in nationally representative datasets.
Changes: Lines 114-117, 130–133 and 142-148.

Some citation clusters feel too dense (e.g., lines 87–93). Integrating references more smoothly into the narrative would improve readability.
Response: The relevant section has been rewritten for improved narrative flow.
Changes: Lines 86-88.

The introduction concludes with bullying, which is a strong point, but it would be better to conclude with a clear lead-in to the study's aims.
Response: Thank you for this suggestion. We revised the end of the Introduction to include a clear transition into the study aims and to explicitly state the purpose of the

analysis, as well as the socio-ecological framing. The revised concluding paragraph now summarizes the key interpersonal variables and states how these inform the research objectives.

Changes: Lines 114-117.

The text switches between "children" and "adolescents." Since the dataset covers the age range of 0–17, the authors should consider clarifying upfront what "children" means in this study.

Response: The study population defines the age group from 6-17 years.

Changes: Lines 185-186.

Methods:

Since all outcomes are parent-reported, there is a real risk of bias, especially for sensitive issues like mental health and bullying. Authors should address the validity of these reports and whether NSCH has validation data.

Response: We added a discussion of potential reporting bias and referenced NSCH validation documentation.

Changes: Lines 195-198.

Merging current and lifetime diagnoses of depression/anxiety into a single binary outcome could mask important differences. Authors should justify this decision or provide sensitivity analyses with separate categories.

Response: Thank you for this critical methodological observation. In the NSCH dataset, the depression and anxiety measures are structured as two-part questions indicating whether the child has **ever** been diagnosed and whether the condition is **current**. Given the relatively low prevalence of current diagnoses and the well-documented instability of episodic internalizing symptoms in youth, we used a combined lifetime indicator to ensure adequate statistical power and reduce sparse cell bias in stratified interaction models. This approach has been used in prior epidemiological analyses of NSCH mental health outcomes. Nonetheless, to address the reviewer's concern, we have now added justification for this analytic decision in the Methods section.

Changes: Lines 204-211.

Parent-reported height/weight may misclassify BMI. This limitation should be acknowledged, ideally with references to validation work.

Response: We agree and have now explicitly acknowledged the potential for measurement misclassification due to parent-reported height and weight. We added references to validation studies indicating that while parent-reported values may slightly underestimate BMI, the overall accuracy is generally acceptable for population-level research using NSCH and similar national surveys.

Changes: Lines 195-198.

Moderators (bullying, friendships, parental mental health, resilience) are well-chosen, but more detail on how they were measured would be helpful. Were these scales validated?

Response: Thank you for this helpful suggestion. We have now expanded the Methods section to detail how each moderator variable was assessed in the NSCH. These measures are standardized surveillance items used consistently across federal child health monitoring systems. Although they are single-item indicators rather than multi-item psychometric scales, prior NSCH technical documentation indicates that these indicators have acceptable construct validity for population-level behavioural surveillance. We also clarified that these measures capture caregiver-reported perceptions and thus reflect the interpersonal context as experienced and interpreted within family environments.

Changes: Lines 228-232, 235-238, 241-244, and 249-251.

Excluding 23,715 cases is significant. Did excluded participants differ from included ones in key demographics or health factors? A brief comparison would strengthen confidence.

Response: We included a brief comparison showing no significant demographic differences between participants who were included and those who were excluded.

Changes: Lines 324-326.

The analytic approach is sound, but it needs further justification. Why quasibinomial instead of standard logistic regression? Were multicollinearity checks done? Were interactions pre-specified?

Response: We have expanded the Methods section to justify our analytic choices. We used quasibinomial logistic regression to account for mild overdispersion in binary outcome models, which is recommended in large population survey analyses when variance exceeds the binomial assumption. We also conducted multicollinearity diagnostics using variance inflation factors (VIF), which indicated no concerning collinearity among covariates. Finally, the interaction terms for bullying, friendship difficulty, parental mental health, and family resilience were pre-specified based on the socio-ecological theoretical framework. These details have now been added to the manuscript.
Changes: Lines 292-299.

Authors should strive to maintain consistent terminology (e.g., LDD/LDA). Currently, it alternates between full and shorthand labels.
Response: We have now standardized terminology for the mental health outcome variables across the manuscript. The terms **“lifetime diagnosis of depression (LDD)”** and **“lifetime diagnosis of anxiety (LDA)”** are each defined once at first mention, and the corresponding abbreviations are used consistently throughout the text, tables, and figure notes. We also reviewed the manuscript to ensure that no inconsistent or outdated labels remain.
Changes: Lines 206-211, and in relevant places in methods and results.

The explanation of FPL categories could be simplified, perhaps with a small table instead of dense text.
Response: We agree that the previous text-based description of Federal Poverty Level (FPL) categories was dense. We replaced this paragraph with a concise table that summarizes each FPL category and its corresponding household income meaning. This improves readability and clarity for the reader.
Changes: Lines 318.

Results:

Double-check formatting and numbers. For example, in Table 2, some ORs/CI ranges look off (e.g., “1.53(1.18-197)”).

Response: Thank you for bringing this to our attention. We audited all odds ratios and confidence intervals and corrected both a numeric typo **and** formatting inconsistencies in Table 2 (and mirrored the same standards in Tables 3 and 4). Specifically, the adjusted OR for Overweight → Depression was mistakenly rendered as *1.53(1.18–197)* in the working Word file; this has been corrected to *1.53 (1.18–1.97)*. We also standardized spacing, symbols, and decimal precision across all tables. The corrected values and formatting are now reflected in the revised manuscript.

Changes: Narrative text: standardized “95%CI” → “95% CI” and spacing around “OR” (e.g., *OR=1.20 → OR 1.20* or *OR = 1.20*, per journal style) in the Results paragraph preceding Table 2.

Global formatting rules applied to all tables:

1. Space before CIs: *1.20(1.01–1.43) → 1.20 (1.01–1.43)*,
2. En dash (–) in CI ranges,
3. Two-decimal precision for ORs and CI bounds,
4. Consistent “aOR” label in table body and notes,
5. “95% CI” spelled with a space.

<u>The transitions between tables/figures and the narrative feel abrupt. Briefly summarizing the key takeaway after each would help readers follow along.</u>

Response: We agree that adding brief interpretive transition sentences would improve readability. We have now added a concise summary statement after each major table summarizing the key takeaway before moving into the stratified results. These additions do not introduce new interpretation but enhance narrative continuity and flow.

Changes: Lines: 320-323, 325-327, 340-341, 370-373, 397-399, and 415-417.

<u>With so many stratified analyses, the type I error risk rises. Were any corrections (Bonferroni, FDR) applied? If not, authors should consider acknowledging this.</u>

Response: We added statements in both the Methods and Discussion sections acknowledging this and describing the stratified analyses as exploratory.

Changes: Lines 297-299, and 549-551.

<u>For non-significant results, rather than just saying “not significant,” point to the CI overlap with the null to show strength of evidence.</u>

Response: We now reference confidence intervals crossing the null instead of stating "not significant."
Changes: Lines 352-356 and 444-449.

Discussion:

Some phrasing suggests causation when the design only supports associations. Use more cautious wording (e.g., "associated with" instead of "contributes to").
Response: All causal language has been replaced with associational phrasing.
Changes: Lines 477-479 and other relevant places.

The explanation for the distinction between infrequent and frequent bullying is speculative. Authors should clearly frame it as a hypothesis, cite relevant resilience literature, and avoid overextending the claim.
Response: This explanation is now explicitly framed as a hypothesis, supported by resilience literature, and identified as requiring longitudinal confirmation.
Changes: Lines 485-492.

The emphasis on equity and multi-level interventions is strong, but it is essential to avoid overstating what the dataset itself supports. Policy recommendations should be framed as potential applications, not direct conclusions from the data.
Response: We revised to emphasize that proposed interventions are suggested implications rather than conclusions directly derived from causal evidence.
Changes: Lines 526-529.

Also, the minor edits suggested by another reviewer (AJAO) are incorporated at relevant places:
You don't start Abstract with the independent variable, rather you start with the dependent variable (Children's Mental Health).
Response: Agreed
Changes: Lines 45-48.

Please, clarify between the conclusion and recommendation(s) of the study.
Response: Clarified
Changes: Lines 67-72.

Please expound these “few studies” with supported and related literature.
Response: Added the relevant literature.
Changes: Lines 141.

There is no flow in your statement. Please try to restructure the last paragraph of your introduction to show the reason upon which the research was conducted. You ended the “Introduction” abruptly.
Response: Added a para for better flow
Changes: Lines 142-148.

Where? Please cite the supported literature in form of Parenthetical citation(s).
Response: Added citation to data source
Changes: Line 175.

Please use APA format for your Tables
Response: Made edits to all tables
Changes: All tables.

Please ensure that while you write the literatures that support the findings, there is also a need to cite literature that negates the findings too. Do this to all your findings narrative.
Response: Made edits to the discussion accordingly
Changes: Lines added 471-474, 500-503, 516-518.

Please follow 7th Edition of APA referencing style in all your references
Response: Used Vancouver style as given under PLOS guidelines
Changes: No change made.

We are grateful to the reviewers for their insightful comments, which have strengthened the rigor, clarity, and interpretative balance of our manuscript. We believe the revised version is significantly improved and hope it will now meet the journal’s publication standards.

Thank you for your time and consideration.

Sincerely,

Fnu Kajal
