# Supplementary material for: Between the scales and the scars: The interpersonal moderators of the association between Body Mass Index and parent-reported depression/anxiety diagnoses in U.S. children: Evidence from the 2021–22 National Survey of Children’s Health (NSCH)
Source: PLOS Glob Public Health. 2026 May 20;6(5):e0006462. doi: 10.1371/journal.pgph.0006462 (PMC13189307; doi:10.1371/journal.pgph.0006462)
Supplement: Attachment — Submitted filename: REVIEWED ARTICLE-PROF AJAO.docx [file pgph.0006462.s004.docx]

PLOS Global Public Health

**Between the Scales and the Scars: The Interpersonal Burden of Body Weight on Children’s Mental Health: Evidence from the 2021- 22 National Survey of Children’s Health (NSCH)**

--Manuscript Draft--

| **Manuscript Number:** | PGPH-D-25-02696 |
| --- | --- |
| **Article Type:** | Research Article |
| **Full Title:** | Between the Scales and the Scars: The Interpersonal Burden of Body Weight on Children’s Mental Health: Evidence from the 2021- 22 National Survey of Children’s Health (NSCH) |
| **Short Title:** | Interpersonal Determinants of the Body-Mind Burden in the U.S. Children |
| **Corresponding Author:** | Fnu Kajal  The University of Arizona Mel and Enid Zuckerman College of Public Health Tucson, UNITED STATES OF AMERICA |
| **Order of Authors:** | Fnu Kajal |
|  | Bin Suh |
|  | Zhao Chen |
|  | Maia ingram |
|  | Scott Carvajal |
|  | Karl Krupp |
|  | Purnima Madhivanan |
| **Keywords:** | obesity, mentalhealth, interpersonal, NSCH, bullying |
| **Abstract:** | Background:  Childhood obesity has emerged as a significant public health concern, with an increasing body of evidence correlating it with a heightened risk of lifelong diagnosis of depression and anxiety(LDD/LDA). This study aims to examine the impact of less- explored interpersonal dynamics on the association between obesity and lifetime diagnoses of depression and anxiety in children.  Methods: A cross-sectional study of 40,820 U.S. children aged 6-17 used survey- weighted logistic regression on the 2021-22 NSCH data. The study examined the moderating effects of bullying, peer relationships, parental mental health, and family resilience on mental health outcomes (LDD & LDA), utilizing interaction terms categorized by Body Mass Index (BMI), adjusted for age, sex, race/ethnicity, and poverty levels.  Results:  Obesity was significantly associated with higher odds of both depression and anxiety in children aged 6-17 years. Obese children had 2.08 times higher odds of depression (aOR 2.08; 95% CI: 1.64-2.64) and 1.48 times higher odds of anxiety (aOR 1.48; 95% CI: 1.25-1.76) compared to normal-weight peers. Significant moderating effects were observed for depression (adjusted for age, sex, race/ethnicity and poverty levels): never bullied (aOR = 2.12; 95% CI: 1.45-3.11), maternal mental health (aOR = 1.70; 95% CI: 1.70- 2.70), and paternal mental health (aOR = 2.54; 95% CI: 1.51-4.29).  For anxiety, moderating effects were seen for making friends (aOR = 1.91; 95% CI: 1.13-3.22) and father mental health (aOR = 2.14; 95% CI: 1.34-3.41).  Conclusions:  Obesity is associated with interpersonal stressors, which in turn, significantly escalate the risk of the development of depression and anxiety in children. Public health interventions must focus on addressing modifiable relational environments, including bullying and familial relationships, to disrupt the pathways connecting obesity with mental illness in children. |
| **Additional Information:** |  |

| **Question** | **Response** |
| --- | --- |
| **Financial Disclosure**  Enter a financial disclosure statement that describes the sources of funding for the work included in this submission and the role the funder(s) played. This includes grants and any commercial funding of the work or authors.  This statement will be typeset if the manuscript is accepted for publication.  *Please review the* [*submission guidelines*](http://journals.plos.org/globalpublichealth/s/submission-guidelines#loc-financial-disclosure-statement) *and the instructions link below for detailed requirements and guidance.* | The author(s) received no specific funding for this work." |
| **Competing Interests**  On behalf of all authors, disclose any competing interests that could be perceived to bias this work.  This statement will be typeset if the manuscript is accepted for publication.  *Please review the instructions link below and PLOS Global Public Health’s* [*competing interests*](https://journals.plos.org/globalpublichealth/s/competing-interests) *policy to determine what information must be disclosed at submission.* | NA |
| **Data Availability**  Before publication, Authors are required to make fully available and without restriction all data underlying their findings. Please see our [PLOS Data](https://journals.plos.org/globalpublichealth/s/data-availability) [Policy](https://journals.plos.org/globalpublichealth/s/data-availability) page for detailed information on this policy.  A **Data Availability Statement**, detailing where the data can be accessed, is | All data and related meta-data underlying reported findings are available in public data repository (https://nschdata.org/browse/survey/results?q=10511&r=1) |

| required at first submission. Insert your Data Availability Statement in the box below.  Please see the [data reporting](https://journals.plos.org/globalpublichealth/s/submission-guidelines#loc-data-reporting) section of our submission guidelines for instructions on what you need to include in your Data Availability Statement.  This statement will be typeset if the manuscript is accepted for publication.  *PLOS allows rare exemptions to address legal and ethical concerns. If you have legal or ethical restrictions, please use the box below to detail these in full sentences for the Journal team to consider.* |  |
| --- | --- |

Manuscript	[Click here to access/download;Manuscript;Main text paper 1](https://www2.cloud.editorialmanager.com/pgph/download.aspx?id=626117&guid=e58b8a1e-3122-4f89-b903-f8ef34f3f733&scheme=1)[<https://www2.cloud.editorialmanager.com/pgph/download.aspx?id=626117&guid=e58b8a1e-3122-4f89-b903-f8ef34f3f733&scheme=1> V4.docx](https://www2.cloud.editorialmanager.com/pgph/download.aspx?id=626117&guid=e58b8a1e-3122-4f89-b903-f8ef34f3f733&scheme=1)

1 **Between the Scales and the Scars: The Interpersonal Burden of Body Weight**
2 **on Children’s Mental Health: Evidence from the 2021- 22 National Survey of**
3 **Children’s Health (NSCH)**
4 Fnu Kajal^1^, Bin Suh^2^, Chen Zhao^3^, Maia Ingram^4^, , Scott Carvajal^5^, Karl Krupp^6^,
5 Purnima Madhivanan^7^

# 6 Corresponding Author’s Details:

7 ^1^Fnu Kajal, M.D., M.Sc., DrPH Candidate, Mel & Enid Zuckerman College of Public
8 Health, University of Arizona.
9 Email: [kkajal@arizona.edu](mailto:kkajal@arizona.edu)
10 ORCiD: <https://orcid.org/0000-0002-7971-0237>

11

12 ^2^Bin Suh, Senior Epidemiologist, Arizona Department of Health Services.
13 Email: [bin.suh@azdhs.gov](mailto:bin.suh@azdhs.gov)
14 ORCiD: <https://orcid.org/0000-0001-8853-4709>

15

16 ^3^Zhao Chen, PhD, MPH, Distinguished Professor, Epidemiology and Biostatistics, Mel
17 & Enid Zuckerman College of Public Health, University of Arizona.
18 Email: [zchen@arizona.edu](mailto:zchen@arizona.edu)
19 ORCiD: <https://orcid.org/0000-0002-5659-0447>

20

21 ^4^Maia Ingram, MPH, Director of Participatory Research and Practice and Co-Director,
22 Arizona Prevention Research Center, Mel & Enid Zuckerman College of Public Health,
23 University of Arizona.
24 Email: [maiai@arizona.edu](mailto:maiai@arizona.edu)
25 ORCiD: <https://orcid.org/0000-0002-6517-7697>

26

27 ^5^Scott Carvajal, PhD, Departmental Chair and Professor, Health Promotion Sciences,
28 Mel & Enid Zuckerman College of Public Health, University of Arizona..
29 Email: [carvajal@arizona.edu](mailto:carvajal@arizona.edu)
30 ORCiD: <https://orcid.org/0009-0003-2106-5305>

31

32 ^6^Karl Krupp, MSc, PhD, Assistant Professor, Division of Public Health Practice &
33 Translational Research, Mel & Enid Zuckerman College of Public Health, University of
34 Arizona.
35 Email: [kkrupp@arizona.edu](mailto:kkrupp@arizona.edu)
36 ORCiD: <https://orcid.org/0000-0001-7001-9194>

37

38 ^7^Purnima Madhivanan, MBBS, MPH, PhD, Associate Professor, Health Promotion
39 Sciences, Mel & Enid Zuckerman College of Public Health, University of Arizona.
40 Email: [pmadhivanan@arizona.edu](mailto:pmadhivanan@arizona.edu)
41 ORCiD: <https://orcid.org/0000-0001-7818-3394>

42

43	**Abstract**

44

**Background:**

45 Childhood obesity has emerged as a significant public health concern, with an
46 increasing body of evidence correlating it with a heightened risk of lifelong diagnosis
47 of depression and anxiety(LDD/LDA). This study aims to examine the impact of less-
48 explored interpersonal dynamics on the association between obesity and lifetime
49 diagnoses of depression and anxiety in children.

50

**Methods:**

51 A cross-sectional study of 40,820 U.S. children aged 6-17 used survey-weighted
52 logistic regression on the 2021-22 NSCH data. The study examined the moderating
53 effects of bullying, peer relationships, parental mental health, and family resilience
54 on mental health outcomes (LDD & LDA), utilizing interaction terms categorized by
55 Body Mass Index (BMI), adjusted for age, sex, race/ethnicity, and poverty levels.

56

57

**Results:**

58 Obesity was significantly associated with higher odds of both **depression** and
59 **anxiety** in children aged 6-17 years. Obese children had **2.08 times higher odds** of
60 depression (aOR 2.08; 95% CI: 1.64-2.64) and **1.48 times higher odds** of anxiety
61 (aOR 1.48; 95% CI: 1.25-1.76) compared to normal-weight peers. Significant
62 moderating effects were observed for **depression (adjusted for age, sex,**
63 **race/ethnicity and poverty levels)**: never bullied (aOR = 2.12; 95% CI: 1.45-3.11),
64 maternal mental health (aOR = 1.70; 95% CI: 1.70- 2.70), and paternal mental health
65 (aOR = 2.54; 95% CI: 1.51-4.29). For **anxiety**, moderating effects were seen for
66 **making friends** (aOR = 1.91; 95% CI: 1.13-3.22) and **father mental health** (aOR =
67 2.14; 95% CI: 1.34-3.41).

68

**Conclusions:**

69 Obesity is associated with interpersonal stressors, which in turn, significantly escalate
70 the risk of the development of depression and anxiety in children. Public health
71 interventions must focus on addressing modifiable relational environments, including
72 bullying and familial relationships, to disrupt the pathways connecting obesity with
73 mental illness in children.

# 74 Introduction:

75 Childhood obesity has emerged as a pressing public health concern with significant
76 implications for both physical and mental well-being. On a global scale, childhood
77 obesity has proliferated at an unprecedented rate in recent decades. According to
78 the World Health Organization, it was estimated in 2022 that more than 390 million
79 children aged 5 to 19 were classified as overweight, and 160 million were obese
80 (1). In the United States, nearly 20 percent of children experience obesity, and the
81 rates of childhood obesity have increased fourfold since 1990 (2), forming what is
82 increasingly recognized as a “Body–Mind Burden”(3,4). Concomitantly, the incidence
83 of mental health issues in children has spiked, with nearly 50% of adolescents
84 experiencing at least one mental illness, and emergency visits for mental health
85 issues rising by 37% in recent years (5,6).
86 This epidemiological convergence is not coincidental. There is increasing evidence
87 for a bidirectional relationship between obesity and mental health. Individual meta-
88 analyses suggest that children who are overweight or obese are at an elevated risk of
89 experiencing depression (7,8), anxiety (9), and social withdrawal (10). The reverse
90 also holds: adolescents with depression have a 70% increased risk of obesity,
91 particularly among females (11,12).. Luppino et al. established this reciprocal risk in a
92 seminal meta-analysis, finding that obesity heightens depression risk, and vice versa
93 (13).
94 Although biological and behavioural determinants (e.g., dietary intake and physical
95 inactivity) play a role in these associations, they do not fully account for the emotional
96 distress presented by children with obesity. Instead, the pathways seem to be
97 socially mediated. Teasing/Bullying, stigma, parental mental health, family routines,
98 and peer dynamics are increasingly implicated as effect modifiers in this relationship
99 (14–16). Yet, few studies have comprehensively modelled these interpersonal
100 variables.
101 Among these, bullying emerges as an exceptionally robust interpersonal risk factor.
102 Children with obesity have up to 46% higher odds of being bullied, compared to their

103

104

105

106

107

108

109

110

healthy-weight peers (17).

***According to the standards established by the World Health Organization (WHO), obesity is defined as a measurement that surpasses two standard deviations above the WHO growth reference median (World Health***

***Organization [WHO], 2024). The Centers for Disease Control and***

***Prevention (CDC) defines childhood obesity as having a body mass index (BMI) that is at or exceeds the 95th percentile for age and sex (CDC US Government, 2024).***

111 Victimization, especially related to weight, can exacerbate internalized stigma and
112 trigger depressive and anxious symptomatology (18,19). Longitudinal studies show
113 that the mental health impact of obesity is significantly magnified among children who
114 are bullied (20,21). Additionally, children classified as bully-victims experience the
115 highest rates of depression, suicidal ideation, and behavioural problems (22,23).
116 Peer relationships more broadly also serve as salient moderators. Quality friendships
117 and strong peer attachment buffer children against the psychological impact of social
118 exclusion due to obesity (24,25). Increasing evidence indicates, that secure peer
119 attachment is protective for emotional health and well-being (26), whilst peer rejection
120 evokes distress both within and outside the context of overt bullying (27,28).
121 In the familial sphere, parental mental health—particularly maternal depression—has
122 consistently been linked to both child obesity and mental health issues. Maternal
123 depression influences parenting styles, feeding practices, emotional availability, and
124 home routines, all of which mediate the pathway from obesity to poor emotional
125 outcomes (29–31). The risk is heightened with chronic depressive symptoms, as
126 shown in cohort studies and meta-analyses (32,33). However, paternal mental health
127 remains underexplored, and few studies have disaggregated effects by caregiver role
128 or gender (34,35).
129 Family resilience—defined as the ability of families to adapt positively to adversity—
130 has also emerged as a protective factor. Findings from studies that used the NSCH
131 data have shown that family resilience can attenuate the deleterious effects of
132 bullying and adversity on internalizing symptoms (36,37). For example, a study using
133 data from the COVID-19 pandemic found that family functioning was positively
134 related to adolescent anxiety and depression(38,39). Yet, its role in buffering the
135 psychological consequences of obesity has been underexplored, with few studies
136 testing interaction effects in nationally representative datasets.

# 137 Theoretical Framework:

138 Mental health conditions in children are multifactorial, necessitating multidimensional
139 theoretical frameworks. This research adopts both the **socio-ecological model**
140 **(SEM)** and the **diathesis-stress model** to conceptualize the interplay between
141 obesity and mental health outcomes in youth.
142 The **SEM**, originally proposed by Bronfenbrenner and expanded by McLeroy et al.
143 into five levels of influence—Intrapersonal, Interpersonal, Institutional, Community,
144 and Public Policy—underscores the dynamic interrelations between individuals and
145 their environments (40,41). While intrapersonal factors like sleep and activity
146 patterns are essential, interpersonal stressors such as bullying, parental mental
147 health, peer isolation, and family resilience are increasingly recognized as critical
148 modifiers of mental health outcomes in children with obesity.
149 The diathesis-stress model dovetails with this ecological lens and explains how
150 genetic and physiological predispositions intersect with environmental adversities to
151 create mental illness (42,43). We view obesity in this framework as a biological
152 condition and psychosocial stressor that is experienced with stigma and social
153 rejection. Nonetheless, these adversities may be ameliorated by protective
154 interpersonal factors (e.g., emotionally balanced parenting and positive peer
155 support). In tandem, such models provide a comprehensive framework for
156 understanding how relational life experiences impact the emotional consequences of
157 childhood obesity.
158 A recent investigation identified the omission of considering social factors, such as
159 bullying, peer relationships, and family environment, as a crucial issue (44,45). To
160 address these critical gaps, the present study will explore how interpersonal
161 variables—bullying, peer relationships, parental mental health, and family
162 resilience—influence the association between BMI and mental health outcomes using
163 data from the 2021–22 National Survey of Children’s Health (NSCH). To account for
164 this, we employed the socio-ecological framework and survey-weighted logistic
165 regression models that examined both main effects and interaction effects. This
166 nested approach provided a valuable understanding of the relational and
167 psychosocial dimensions of obesity and mental health in childhood, informing
168 interventions that work beyond individual behavior to include family, peer, and social
169 aspects.

# 170 METHODOLOGY

171 **Data Source**
172 The data for this study were obtained from the Child and Adolescent Health
173 Measurement Initiative (CAHMI)/NSCH for the 2021-2022 period, which is publicly
174 available. The NSCH, sponsored by the U.S. Department of Health and Human
175 Services, is conducted by the Census Bureau and has been providing nationally
176 representative data on children's health since 2002-2003. The survey uses
177 instruments whose reliability and validity have been supported in numerous studies.

178

# 179 Study Design

180 This study is a secondary data analysis of a cross-sectional survey examining the
181 association between obesity and mental health issues, particularly depression and
182 anxiety. It focuses on identifying key interpersonal determinants that modify the
183 association between obesity and mental health issues in children.

# 184 Study Population

185 The study population consists of children and adolescents aged 6 to 17, as reported
186 in the 2021-2022 NSCH survey (46). This diverse and nationally representative
187 sample highlights a critical developmental period where obesity and mental health
188 intersect, making it an ideal setting for investigating these issues and ensuring
189 credible, actionable data to support research objectives.
190 The current research represented a sample size of 40,820 children, providing
191 nationally representative data on their health and well-being. Data collection occurred
192 between June 2021 and January 2022. The participants were the parents and
193 guardians of children. The data were collected via the web (90%) and mail (10%),
194 with an approximate response rate of 65%. About 90% of the sample received
195 incentives.
196 **Data Collection Tools/Measures:** All measures were parent-reported (46).
197 **Outcomes (Mental Health Measures):** The outcome variables were ‘depression’
198 and ‘anxiety’ as assessed using the Revised Child Anxiety and Depression Scale
199 (47). Regarding depression and anxiety, responses encompass the current
200 diagnosis and historical data for children aged 6-17. The responses related to
201 depression and anxiety were classified as binary responses for the purpose of
202 analysis. Lifetime diagnosis of depression (LDD)/ Lifetime Diagnosis of Anxiety
203 (LDA) refers to children who have ever been diagnosed with depression/anxiety,
204 including those who are currently depressed, and those who were previously
205 diagnosed but no longer meet the criteria. We combined current depression/anxiety,
206 and those who had ever been diagnosed as one category, and those who are not
207 currently experiencing depression or anxiety, for this comparison.
208 **Explanatory Variable (BMI):** Obesity was measured using Body Mass Index (BMI)
209 based on parent-reported height and weight, categorized according to CDC
210 guidelines (CDC, 2016) into four groups by age-adjusted percentile: Underweight
211 (less than 5th percentile), Normal weight (5th to 84th percentile), Overweight (85th to
212 94th percentile), and Obese (95th percentile or above)
213 **Covariates:** Covariates included the child’s age, sex, race/ethnicity, and federal
214 poverty levels (FPL), which represent the socioeconomic status of the family. The
215 question on the FPL is: “What is the income level (federal poverty level, FPL) of the
216 household that this child lives in? The responses are divided into 0-99% FPL; 100-
217 199% FPL; 200-399% FPL; 400% FPL or greater.
218 **Moderators:** These include interpersonal factors that reflect social relationships and
219 peer interactions.
220 Bullying was assessed by asking respondents to report how often the child
221 experienced bullying in the past 12 months. For the purpose of analysis, the
222 responses were categorized into three categories: levels: “Never in past 12
223 months”= Never; “1-2 times in past 12 months” and "1-2 times per month," into
224 Occasional and “1-2 times per week” and “Almost every day” into Frequent.
225 Making or keeping friends involved respondents indicating the child's level of difficulty
226 in forming and maintaining friendships. The responses provided for this evaluation
227 included "No difficulty," "A little difficulty," and "A lot of difficulty."
228 Father's/Mother’s mental health was evaluated by asking respondents to assess the
229 mental health status of the father/mother, if he/she was the primary caregiver. The
230 assessment options included "Excellent/very good", "Good", and "Fair/poor."
231 Family resilience was evaluated by respondents who assessed the family's ability to
232 cope during difficult times. The question asked was: Does this child live in a home
233 where the family demonstrates qualities of resilience during difficult times? The
234 options included “All or most of the time to 0-1 items”, “All or most of the time to 2-3
235 items”, “All or most of the time to all 4 items.”
236 A non-response bias analysis by NSCH showed no signs of bias. We applied
237 weights, stratification, and accounted for clustering to enhance generalizability to the
238 U.S. population of children and provided an unbiased test of statistical significance.
239 The analytic sample included only children with complete responses on BMI,
240 depression, and anxiety questions. Children under 6 years were excluded to avoid
241 sensitivities related to BMI calculations and varying developmental stages. Of the
242 original 64,535 children aged 6–17 years, we excluded 23,715 cases with non-
243 analytic codes (95 were less than 2 or 3 or 5 years old; 99 were missing or refused)
244 for essential study variables. The ultimate analytic sample was comprised of 40,820
245 children who had valid information on all variables of interest. An inspection of the
246 missing data pattern revealed no residual missingness and no need for multiple
247 imputation.

# 248 IRB and Ethics Statement:

249 These survey results are openly available and de-identified; therefore, secondary
250 analysis of the results is, according to federal guidelines, exempt from consideration
251 by an Institutional Review Board (IRB).

# 252 Analysis:

253 The analysis used the R program and survey-weighted statistical procedures to
254 address the NSCH’s complex sampling design, correcting for oversampling, non-
255 response, and stratification issues. The NSCH data included weights that adjust for
256 unequal selection probabilities, non-response, and post-stratification to accurately
257 represent the population, as per the NSCH survey codebook (46).
258 The descriptive analysis includes both unweighted and weighted frequencies and
259 percentages across demographic subgroups. A bivariate analysis examined
260 relationships between BMI categories and LDD and LDA, as well as interpersonal
261 variables. Design-based chi-square tests identified statistically significant
262 associations. LDD or LDA were analyzed across demographic and interpersonal
263 variable categories, as well as BMI categories, for statistical associations. Survey-
264 weighted logistic regression models were used to evaluate the association between
265 BMI and LDD & LDA, controlling for covariates and testing interactions with
266 interpersonal variables. Adjusted odds ratios (aOR) and 95% confidence intervals
267 (95%CI) are reported, with statistical significance set at p < 0.05. Survey design
268 variables, including weights, strata, and primary sampling units, were incorporated
269 using the svyglm() function in R, with models defined using a quasibinomial family to
270 address overdispersion in binary outcomes.

# 271 RESULTS

272 In this study, the sample (Table 1), represented approximately 29 million children in
273 the United States. The sample was evenly distributed among children aged 6-11
274 (49.3%), and those aged 12-17 years (50.7%). Similarly, the sample was balanced
275 between male (50.9%) and female (49.0%) participants. While 57.3% were non-
276 Hispanic White, 7.5% were Non-Hispanic Black, and 11.6% reported
277 other/multiracial. Furthermore, it was found that 60.5% of the children had a normal
278 BMI, while 15.1% were classified as overweight, and 15.3% obese. An LDA was
279 reported in 13.3% of the participants, whereas an LDD occurred in 4.5%. In terms of
280 interpersonal variables, 63.8% of children reported never being bullied within a year.
281 For kids who were bullied, 26.6% were bullied once or twice within the past year, with
282 5.7% bullied once or twice per month, and lower percentages bullied several times
283 (2.7% per week, 1.1% per day). Additionally, 4.2% of the children indicated difficulties
284 in making friends. It is also noteworthy that 4.5% and 6.0% of children’s parental
285 guardians (father/male and mother/female, respectively) exhibited poor mental health
286 status. Lastly, only 5.2% of families demonstrated characteristics of full resilience
287 across all four assessed items.

2

| 88 **Table 1: Descriptive Stats (n=40820) of all children 6-17 years in NSCH Survey 21-22:** | | | |
| --- | --- | --- | --- |
| **Variable** | **Categories** | **Unweighted frequency** | **Weighted frequency (in millions)** |
|  |  |  | **(Weighted %)** |
| **Age** | 6-11 years | 19108 | 14.4 (49.3%) |
|  | 12-17 years | 21712 | 14.8 (50.7%) |
| **Sex** | Male | 21205 | 14.9 (50.9%) |
|  | Female | 19615 | 14.3 (49.1%) |
| **Race/Ethnicity** | Hispanic | 5072 | 6.9 (23.5%) |
|  | White, Non-Hispanic | 28911 | 16.7 (57.3%) |
|  | Black, Non-Hispanic | 1445 | 2.2 (7.5%) |
|  | Other/Multiracial, non-Hispanic | 5392 | 3.4 (11.6%) |
| **Federal poverty level** | 0-99%FPL | 3132 | 3.1 (10.6%) |
|  | 100-199%FPL | 5161 | 4.7 (16.2%) |
|  | 200-399%FPL | 12206 | 9.0 (31.0%) |
|  | 400% FPL or greater | 20321 | 12.3 (42.1%) |
| **BMI** | UW | 3613 | 2.7 (9.1%) |
|  | Normal | 25952 | 17.7 (60.5%) |
|  | Overweight | 5806 | 4.4 (15.1%) |
|  | Obese | 5449 | 4.5 (15.3%) |
| **Anxiety** | No anxiety | 34173 | 25.3 (86.7%) |
|  | Lifetime diagnosis of anxiety | 6647 | 3.9 (13.3%) |
| **Depression** | No Depression | 38434 | 27.9 (95.5%) |
|  | Lifetime diagnosis of Depression | 2386 | 1.3 (4.5%) |
| **INTERPERSONAL FACTORS** | | | |

| **Bullying** | Never  in past 12 months | 24129 | 18.6 (63.8%) |
| --- | --- | --- | --- |
|  | 1-2 times  in past 12 months | 11959 | 7.8 (26.6%) |
|  | 1-2 times per month | 2874 | 1.7 (5.7%) |
|  | 1-2 times per week | 1281 | 0.8 (2.7%) |
|  | Almost everyday | 577 | 0.3 (1.1%) |
| **Making or Keeping friends** | No difficulty | 30212 | 22.3 (76.4%) |
|  | A little Difficulty | 8532 | 5.7 (19.4%) |
|  | A lot of Difficulty | 2076 | 1.2 (4.2%) |
| **Mental Health of Father** | Excellent or Very Good | 30420 | 22.0 (75.4%) |
|  | Good | 8405 | 5.9 (20.1%) |
|  | Fair or Poor | 1995 | 1.3 (4.5%) |
| **Mental Health of Mother** | Excellent or Very Good | 29043 | 21.0 (71.8%) |
|  | Good | 9368 | 6.5 (22.2%) |
|  | Fair or Poor | 2409 | 1.7 (6.0%) |
| **Family Resilience** | All or most of time to all 4 items | 1899 | 1.5 (5.2%) |
|  | All or most of time to 2-3 items | 3791 | 2.7 (9.2%) |
|  | All of most of time to all 0-1 items | 35130 | 25.0 (85.6%) |

289

290 The analysis in Table 2 demonstrates a significant association between obesity and
291 the occurrence of LDD and LDA among children aged 6 to 17 years. In the initial
292 model, obese children exhibited 1.60 times higher odds of experiencing LDD
293 compared to those with a normal BMI (OR= 1.60; 95% CI: 1.27-2.02). Furthermore,
294 after adjusting for age, race, sex, and FPL, this association intensified, revealing that
295 obese children had 2.08 times higher odds of experiencing depression (aOR 2.08;
296 95%CI: 1.64-2.64). It was also observed that there exists a dose-response
297 relationship, whereby overweight and obese children present progressively greater
298 odds of depression than their normal weight counterparts.

299

**Table 2: Multivariate Logistic Regression for BMI and LDD/LDA.**

| **Variable** | **BMI Category** | | **Depression** | | **Anxiety** | |
| --- | --- | --- | --- | --- | --- | --- |
|  |  | | **Unadjusted** | **Adjusted*** | **Unadjusted** | **Adjusted**** |
|  |  |  | **OR (95% CI)** | **OR (95% CI)** | **OR (95% CI)** | **OR (95% CI)** |
|  | ormal (referenc | | 1.00 | 1.00 | 1.00 | 1.00 |
|  | UW | | 0.63(0.46-0.87) | 0.91(0.65-1.27) | 0.85(0.70-1.02 | 1.03(0.85-1.24) |
|  | Overweight | | 1.41(1.07-1.84) | 1.53(1.18-197) | 0.92 (0.80-1.07 | 1.01 (0.87-1.18) |
|  | Obese | | 1.60(1.27-2.02) | 2.08(1.64-2.64) | 1.20(1.01-1.43 | 1.48(1.25-1.76) |
| **Adjusted*** | | Adjusted for age, sex, race and FPL levels | | **Adjusted**** Adjusted for age, sex, and race | | |

300

301 With anxiety, obese children had 1.20 times greater odds of LDA than normal weight
302 children in the unadjusted analyses (OR=1.20; 95% CI: 1.01-1.43). After adjustment
303 for age, sex and race/ethnicity, obese children had also significantly greater odds of
304 LDA than their normal weight counterparts, having 1.48 times greater odds of anxiety
305 (aOR 1.48; 95% CI: 1.25-1.76).
306 Stratified analysis (Table 3.1, 3.2 and 3.3) showed that the link between obesity and
307 depression shifts depended on key social factors, especially bullying and the mental
308 health of parents. Among children who were never bullied, those with obesity were
309 more than twice as likely to experience LDD compared to their normal weight peers,
310 when adjusted for age, sex, race/ethnicity and FPL (aOR 2.12; 95% CI: 1.45–3.11).
311 This association also remained significant for children who were bullied occasionally
312 (aOR 1.65; 95% CI: 1.14–2.39), though it was not statistically significant for those
313 who were bullied frequently (aOR 1.35; 95% CI: 0.87–2.08). A similar trend was seen
314 when looking at maternal mental health. Among children whose mothers had
315 excellent or very good mental health, those with obesity had a 28% higher odds of
316 being LDD (aOR 1.28; 95% CI: 1.02–1.62), while in children whose mothers reported
317 poor mental health, the odds jumped to 70% higher (aOR 1.70; 95% CI: 1.07–2.70)
318 as compared to normal weight children. The pattern was even more pronounced
319 when looking at fathers: children with obesity had significantly higher odds of LDD
320 whether their fathers reported excellent (aOR 1.54; 95% CI: 1.17–2.02), good (aOR
321 2.09; 95% CI: 1.35–3.22), or poor mental health (aOR 2.54; 95% CI: 1.51–4.29).
322 Similarly, Figures 1, 2, and 3 presented indicate that children with a history of
323 frequent bullying exhibited significantly greater predicted probabilities of depression
324 across all BMI ranges, particularly among children who were overweight or obese.
325 Furthermore, children whose parents, both mothers and fathers, reported poor
326 mental health demonstrated a significantly increased risk of depression, with a clear
327 gradient observed from excellent to poor parental mental health. These findings
328 corroborate the interactive roles of interpersonal exposures—namely, bullying and
329 parental psychological well-being—in modifying the association between body weight
330 and mental health outcomes among children.

331

**Table 3: Effect of the Interpersonal Factors on the Unadjusted and Adjusted Odds of Lifetime Diagnosis of Depression (LDD) among Children 6-17 years with Obesity**

332

# 333 Table 3.1: Adjusted Odds Ratios (aOR) for LDD by BMI Category, Stratified

334 **by Mother’s Mental Health**

335

| **Mother's**  **Mental Health** | **BMI**  **Category** | **aOR*** | **95% CI** | **p-value** |
| --- | --- | --- | --- | --- |
| **Excellent/ Very Good** | Underweight | 1.04 | 0.80 – 1.35 | 0.766 |
|  | Overweight | 0.94 | 0.77 – 1.14 | 0.516 |
|  | Obese | 1.28 | 1.02 – 1.62 | **0.035** |
| **Good** | Underweight | 1.00 | 0.72 – 1.39 | 0.993 |
|  | Overweight | 0.97 | 0.72 – 1.29 | 0.815 |
|  | Obese | 1.34 | 0.99 – 1.82 | 0.061 |
| **Fair/Poor** | Underweight | 0.88 | 0.44 – 1.74 | 0.704 |
|  | Overweight | 1.16 | 0.75 – 1.79 | 0.503 |
|  | Obese | 1.70 | 1.07 – 2.70 | **0.025** |

336

# 337 Table 3.2: Adjusted Odds Ratios (aOR) for LDDby BMI Category, Stratified

338 **by Father’s Mental Health**

339

| **Father’s Mental Health** | | **BMI**  **Category** | **aOR*** | **95% CI** | **p-value** |
| --- | --- | --- | --- | --- | --- |
| **Excellent/Very Good** | Underweight | | 0.96 | 0.62 – 1.49 | 0.862 |
|  | Overweight | | 1.36 | 1.02 – 1.82 | **0.037** |
|  | Obese | | 1.54 | 1.17 – 2.02 | **0.002** |
| **Good** | Underweight | | 0.82 | 0.42 – 1.57 | 0.544 |
|  | Overweight | | 1.63 | 0.99 – 2.68 | 0.052 |
|  | Obese | | 2.09 | 1.35 – 3.22 | **0.001** |
| **Fair/Poor** | Underweight | | 0.62 | 0.28 – 1.35 | 0.227 |
|  | Overweight | | 1.20 | 0.61 – 2.35 | 0.599 |
|  | Obese | | 2.54 | 1.51 – 4.29 | **0.001** |

340

# 341 Table 3.3: Adjusted Odds Ratios (aOR) for LDD by BMI Category, Stratified

342 **by Bullying Frequency**

343

| **Bullying Frequency** | **BMI**  **Category** | **aOR*** | **95% CI** | **p-value** |
| --- | --- | --- | --- | --- |
| **Never ( data$bullied_2122**  **== 1 ~ "Never")** | Underweight | 1.09 | 0.59 – 2.03 | 0.781 |
|  | Overweight | 1.07 | 0.73 – 1.56 | 0.745 |
|  | Obese | 2.12 | 1.45 – 3.11 | **0.000** |
| **Occasional (data$bullied_2122**  **%in% c(2, 3) ~ "Occasional")** | Underweight | 0.80 | 0.53 – 1.21 | 0.292 |
|  | Overweight | 1.77 | 1.24 – 2.52 | **0.002** |
|  | Obese | 1.65 | 1.14 – 2.39 | **0.008** |
| **Frequent (data$bullied_2122**  **%in% c(4, 5) ~ "Frequent")** | Underweight | 0.69 | 0.32 – 1.45 | 0.324 |
|  | Overweight | 1.51 | 0.81 – 2.81 | 0.194 |
|  | Obese | 1.35 | 0.87 – 2.08 | 0.176 |

344

345 **Figure 1; Predicted Probability of Depression - Bullying Category**

# 346 Figure 2: Predicted Probability of Depression- Mental Health of Father

347

# 348 Figure 3: Predicted Probability of Depression- Mental Health of Mother

349

350

351 In contrast, **difficulty making friends** and **family resilience (Table 3.4)** did not
352 interact with obesity, but both were strong correlates of depression on their own.
353 Children who had a little difficulty making friends had five times higher odds to report
354 LDD (aOR 4.96; 95% CI: 4.07–6.05), and those with a lot of difficulty had almost
355 thirteen times the odds (aOR 12.94; 95% CI: 10.03–16.44), compared to children
356 who reported no difficulty. On the flip side, strong family resilience seemed to offer
357 protection. Children from families that were resilient to “most items” had 43% lower
358 odds of LDD (aOR 0.57; 95%CI: 0.42–0.77), and those from families resilient to
359 “some items” had 60% lower odds (aOR 0.40; 95% CI: 0.31–0.53) compared to
360 families resilient to all items.

# 361 Table 3.4: Adjusted Odds Ratio for LDD by Difficulty in Making Friends and Family

362 **Resilience**

363

|  | **Variable** | **aOR*** | **95% CI** |
| --- | --- | --- | --- |
| **Make friend** | **No Difficulty-Reference** | 1.00 | 1.00 |
|  | **A little difficulty** | 4.96 | 4.07-6.05 |
|  | **A lot of difficulty** | 12.94 | 10.03-16.44 |
|  | **Resilient to all-Reference** | 1.00 | 1.00 |
| **Family Resilience** | **To most items** | 0.57 | 0.42-0.77 |
|  | **To some items** | 0.40 | 0.31-0.53 |

364	Adjusted* for age, sex, race/ethnicity and FPL.

365

366 When examining the relationship of **body weight and social challenges** related
367 to **LDA in children,** the impact of obesity appeared to vary depending on the child’s
368 ability to make friends, and the mental health of their father. Table 4.1 shows that
369 among children who reported **no difficulty making friends,** obesity was not
370 significantly linked to anxiety when adjusted for age, sex, race/ethnicity and FPL
371 (aOR 1.12; 95% CI: 0.87–1.44). The same pattern held for those with **a little**
372 **difficulty,** where the odds of anxiety were slightly higher for children with obesity, but
373 this was not statistically significant (aOR 1.27; 95% CI: 0.94–1.72). However, among
374 children who had **a lot of difficulty making friends,** the findings were different:
375 children with obesity had twice the odds of experiencing anxiety compared to their
376 normal-weight peers (aOR 1.91; 95% CI: 1.13–3.22).

377

378

**Table 4: Effect of the Interpersonal Factors on the Unadjusted and Adjusted Odds of Lifetime Diagnosis of Anxiety(LDA) among Children 6-17 years with Obesity**

379

380

# 381 Table 4.1: Adjusted Odds Ratios (aOR) for LDA by BMI Category, Stratified by

382 **Difficulty in Making Friends**

383

| **Difficulty in Mak**  **Friends** | **BMI Category** | **aOR*** | **95% CI** | **p-value** |
| --- | --- | --- | --- | --- |
| **No difficulty** | Underweight | 1.05 | 0.80 – 1.39 | 0.724 |
|  | Overweight | 1.06 | 0.84 – 1.33 | 0.642 |
|  | Obese | 1.12 | 0.87 – 1.44 | 0.368 |
| **Little Difficulty** | Underweight | 0.75 | 0.52 – 1.08 | 0.125 |
|  | Overweight | 0.79 | 0.61 – 1.03 | 0.081 |
|  | Obese | 1.27 | 0.94 – 1.72 | 0.121 |
| **A Lot of Difficulty** | Underweight | 1.34 | 0.79 – 2.28 | 0.277 |
|  | Overweight | 1.38 | 0.89 – 2.14 | 0.146 |
|  | **Obese** | **1.91** | **1.13 – 3.22** | **0.015** |

384

# 385 Table 4.2: Adjusted Odds Ratios (aOR) for Anxiety by BMI Category, Stratified by

386 **Father Mental Health**

387

| **Father’s Mental Health** | **BMI Category** | **aOR*** | **95% CI** | **p-value** |
| --- | --- | --- | --- | --- |
| **Excellent/Very Good** | Underweight | 1.07 | 0.85 – 1.36 | 0.552 |
|  | Overweight | 1.00 | 0.82 – 1.22 | 0.983 |
|  | Obese | 1.19 | 0.95 – 1.48 | 0.126 |
| **Good** | Underweight | 0.92 | 0.63 – 1.35 | 0.666 |
|  | Overweight | 0.78 | 0.59 – 1.04 | 0.089 |
|  | **Obese** | **1.56** | **1.14 – 2.14** | **0.006** |
| **Fair/Poor** | Underweight | 1.21 | 0.65 – 2.24 | 0.549 |
|  | Overweight | 1.55 | 0.95 – 2.54 | 0.078 |
|  | **Obese** | **2.14** | **1.34 – 3.41** | **0.001** |

388 Adjusted* for age, sex, race/ethnicity and FPL.
389 A similar trend appeared when examining **fathers’ mental health (Table 4.2).** For
390 children whose fathers had **excellent or very good mental health,** obesity was not
391 significantly linked to anxiety when adjusted for age, sex, race/ethnicity and FPL(aOR
392 1.19; 95% CI: 0.95–1.48). Among those whose fathers reported **good** mental health,
393 obesity was associated with a 56% increase in odds of anxiety (aOR 1.56; 95% CI:
394 1.14–2.14). The most substantial effect was seen in children whose fathers had **fair**
395 **or poor mental health**—here, obesity more than doubled the odds of experiencing
396 anxiety (aOR 2.14; 95% CI: 1.34–3.41).
397 Figures 4 and 5 show that the likelihood of anxiety was notably higher in children with
398 obesity when their father reported poor or fair mental health, exceeding 25%.
399 Additionally, children who faced more challenges in forming friendships had a
400 significantly higher risk of anxiety across all BMI categories, especially among those
401 with obesity. These results indicate that paternal mental health and social integration
402 play a crucial role in modifying the association between BMI and anxiety in children.
403 **Figure 4 : Predicted Probability of Anxiety – Making Friends**

# 404 Figure 5: Predicted Probability of Anxiety – Father’s Mental Health

405 **Discussion:**
406 This study provides strong evidence that obesity is significantly associated with both
407 depression and anxiety, even after adjusting for age, sex, race/ethnicity, and poverty
408 levels. This finding is consistent with the existing literature wherein our study
409 reinforces the severe mental health difficulties experienced by obese children, and
410 indicates how it is imperative to recognize obesity as an essential risk factor for
411 depression and anxiety in childhood populations (10,45,48). This research
412 contributes to the literature by revealing that interpersonal stressors exacerbate these
413 risks, aligning with socio-ecological and diathesis-stress models. We observed
414 heterogeneity across BMI groups, particularly between the pathways of depression
415 and anxiety. While the literature corroborates this association for both depression and
416 anxiety, our study has identified a nuance: BMI exhibits a stronger association with
417 depression than with anxiety.
418 Although the literature recognizes bullying as a risk factor for depression and anxiety
419 (49), another paradox identified in this study was that infrequent bullying was more
420 predictive of depression than frequent bullying. This interaction was absent in the
421 development of anxiety in children. The literature does not address this paradox, and
422 we can hypothesize that these findings, while counterintuitive, may result from social
423 desirability bias or selection bias. We can assume that parents have underreported
424 the frequency of bullying, as it engenders stigma or exposes vulnerabilities within the
425 family, particularly when the child conceals the victimization. Another plausible
426 consideration is that children who experience bullying more frequently develop
427 coping strategies, thereby becoming more resilient to the effects of bullying.
428 Therefore, our results indicate that the cumulative impact of infrequent but
429 “unresolved” bullying is greater or more detrimental for children than the frequent
430 bullying that schools or parents actively address. Consequently, we believe that our
431 findings suggest the necessity for screening mental health issues, even amidst
432 seemingly minor or infrequent peer conflicts.
433 The literature has been routine in positing that maternal and paternal mental health
434 have significant effects upon children’s emotional regulation, attachment style, and
435 behavior development (31,34,35). The literature supported that mother-child
436 relationships affect mental health issues more than father-child relationships. The
437 strong link between poor maternal mental health and child depression in our results
438 likely reflects the enduring influence of traditional caregiving roles. As primary
439 caregivers, mothers often provide most of the emotional, educational, and health-
440 related support. Depressed or anxious mothers may be less responsive, nurturing, or
441 emotionally attuned to their children and therefore contribute to poorer caregiving
442 quality, which then indirectly affects the psychosocial development of children, but
443 this would be outside the scope of our research. Mothers face an outsize share of
444 the spadework when it comes to scheduling, cares and comforting as a career so
445 often, they are still very much the hub around which the child's social and emotional
446 world whirls.
447 Despite other interpersonal stresses, resilience appears to be a buffer against
448 depression (50,51). Our findings indicate that, while family resilience is a powerful
449 buffer against depression in children themselves classified as obese, it is not a
450 powerful buffer against anxiety. The distinction may be traced back to the different
451 cognitive and affective mechanisms underpinning these mental health issues.
452 Depression is characterized by a negative schema of hopelessness and self-blame,
453 which may improve more with cohesive family processes. In contrast, anxiety—
454 associated with hyperarousal and vigilance—may necessitate non-family-centred
455 interventions.
456 The findings of this research implore the necessity for multi-level, cross-sectoral
457 interventions, ones that extend beyond the scope of solely biomedical or lifestyle
458 interventions for childhood obesity. Paediatricians and mental health professionals
459 are important in adopting a proactive stance toward screening for depression and
460 anxiety in addition to weight status screening, particularly in children with peer
461 relationship difficulties, bullying history, or parental history of mental health issues.
462 Other findings support the relevance of psychoeducational interventions directed
463 toward parents and caregivers, since parental mental health is a determining factor
464 that influences the emotional growth of children. Parent-focused interventions could
465 enhance children’s emotional well-being, while school-based anti-bullying and peer-
466 bonding programs—especially targeting weight-based discrimination—could address
467 key social risk factors. Policy approaches should address the disproportionate burden
468 on low-income and minority families, embedding obesity and mental health services
469 in equity frameworks. At a policy level, the findings suggest holistic approaches to
470 children's health that consider both physical and mental aspects. Policymakers
471 should also recognize that the mental health disparities associated with obesity
472 differentially impact low-income and minority families, and therefore are of relevance
473 to an equity framework that informs the process of planning and budgeting programs.
474 While providing additional insights into the associations between psychosocial
475 determinants and childhood obesity as well as mental health, several limitations
476 should be acknowledged. The NSCH design is cross-sectional and does not support
477 causal inference. The reliance on parent reports of mental health symptoms and
478 interpersonal behavior (e.g., bullying) may result in recall bias and social desirability.
479 The study was limited in its generalizability by the U.S.-specific health, social and
480 policy context. Future research should utilize longitudinal designs to establish causal
481 directions, involve multiple informants to increase validity, and include qualitative or
482 mixed-method approaches to explain counterintuitive findings such as the lack of a
483 dose–response relationship for infrequent bullying. Future research should confirm
484 the generalizability of these associations in comparative studies across diverse
485 cultural and structural contexts.
486 In sum, obesity in children is embedded within a network of interpersonal and
487 contextual influences that differentially predict depression and anxiety. Multi-level,
488 theory-driven interventions spanning healthcare, education, and family systems are
489 essential to address these intertwined physical and mental health challenges.

# 490 Acknowledgements

491 The author sincerely thanks Dr. Madhivanan, Dr. Chen, Dr. Suh, Dr. Scott Carvajal,
492 Dr. Karl Krupp, and Prof. Maia for their mentorship and guidance in statistics.
493 Gratitude is also expressed to the Arizona Department of Health Services (AZDHS)
494 for their continuous partnership and support in developing this dissertation project.
495 Additionally, the author values the technical infrastructure supplied by the University
496 of Arizona, which facilitated computational and data management needs.

# 497 References

498 1.	World Health Organization [WHO]. Obesity and overweight [Internet]. 2024
499 [cited 2024 Jul 2]. p. 76. Available from: https:/[/www.who.int/news-room/fact-](http://www.who.int/news-room/fact-)
500 sheets/detail/obesity-and-overweight#:~:text=For adults%2C WHO defines
501 overweight and obesity as,to be considered when defining overweight and
502 obesity.
503 2.	CDC US Government. Childhood Obesity Facts [Internet]. 2024. 2024 [cited
504 2025 Mar 15]. Available from: https://[www.cdc.gov/obesity/childhood-obesity-](http://www.cdc.gov/obesity/childhood-obesity-)
505 facts/childhood-obesity-facts.html
506 3.	Di Cesare M, Sorić M, Bovet P, Miranda JJ, Bhutta Z, Stevens GA, et al. The
507 epidemiological burden of obesity in childhood: a worldwide epidemic requiring
508 urgent action. BMC Med [Internet]. 2019;17(1):212. Available from:
509 https://doi.org/10.1186/s12916-019-1449-8
510 4.	Rankin J, Matthews L, Cobley S, Han A, Sanders R, Wiltshire HD, et al.
511 Psychological consequences of childhood obesity: psychiatric comorbidity and
512 prevention. Adolesc Health Med Ther. 2016;Volume 7:125–46.
513 5.	Choi H, Corredor-Waldron A, Currie J, Felton C. WHAT CAN TRENDS IN
514 EMERGENCY DEPARTMENT VISITS TELL US ABOUT CHILD MENTAL
515 HEALTH? Natl Bur Econ Res [Internet]. 2025; Available from:
516 https://[www.medicaid.gov/medicaid/benefits/early-and-periodic-screening-](http://www.medicaid.gov/medicaid/benefits/early-and-periodic-screening-)
517 diagnostic-and-treatment
518 6.	NIHCM Foundation. The State of Children’s Health in the United States
519 [Internet]. 2023. 2023 [cited 2025 Mar 15]. Available from:
520 https://nihcm.org/publications/the-state-of-childrens-health-in-the-united-states
521 7.	Quek Y-H, Tam WWS, Zhang MWB, Ho RCM. Exploring the association
522 between childhood and adolescent obesity and depression: a meta-analysis.
523 Obes Rev an Off J Int Assoc Study Obes. 2017 Jul;18(7):742–54.
524 8.	Sutaria S, Devakumar D, Yasuda SS, Das S, Saxena S. Is obesity associated
525 with depression in children? Systematic review and meta-analysis. Arch Dis
526 Child. 2019;104(1):64–74.
527 9.	Fan Y, Wang D, Wang W, Chen H, Lin J, Lin W, et al. Depression, anxiety,
528 and development of obesity from childhood to young adulthood in China. J
529 Psychosom Res. 2024 Oct;185:111867.
530 10.	Chen Y, Zhang J, Yuan L, Hu H, Li T, Zhao Y, et al. Obesity and risk of
531 depressive disorder in children and adolescents: A meta-analysis of
532 observational studies. Child Care Health Dev. 2024 Mar;50(2):e13237.
533 11.	Mannan MA, Mamun A Al, Doi SAR, Clavarino A. Prospective Associations
534 between Depression and Obesity for Adolescent Males and Females- A
535 Systematic Review and Meta-Analysis of Longitudinal Studies. PLoS One
536 [Internet]. 2016; Available from: https://pubmed.ncbi.nlm.nih.gov/27285386
537 12.	Marmorstein NR, Iacono WG, Legrand LN. Obesity and depression in
538 adolescence and beyond: reciprocal risks. Int J Obes [Internet]. 2014;
539 Available from: https://pubmed.ncbi.nlm.nih.gov/24480863
540 13.	Luppino FS, Wit L de, Bouvy PF, Stijnen T, Cuijpers P, Penninx BWJH, et al.
541 Overweight, Obesity, and Depression A Systematic Review and Meta-analysis
542 of Longitudinal Studies. Arch Gen Psychiatry [Internet]. 2010; Available from:
543 https://pubmed.ncbi.nlm.nih.gov/20194822
544 14.	Puhl RM, King KM. Weight discrimination and bullying. Best Pract Res Clin

545	Endocrinol Metab. 2013 Apr;27(2):117–27.

546 15.	Mesman E, Vreeker A, Hillegers M. Resilience and mental health in children
547 and adolescents: An update of the recent literature and future directions. Curr
548 Opin Psychiatry. 2021;34(6):586–92.
549 16.	Buro AW, Salinas-Miranda A, Marshall J, Gray HL, Kirby RS. Obesity and
550 neurodevelopmental and mental health conditions among adolescents aged
551 10–17 years: The National Survey of Children’s Health 2017–2018. J Paediatr
552 Child Health. 2022;58(10):1753–9.
553 17.	Cheng S, Kaminga AC, Liu Q, Wu F, Wang Z, Wang X, et al. Association
554 between weight status and bullying experiences among children and
555 adolescents in schools: An updated meta-analysis. Child Abuse Negl
556 [Internet]. 2022; Available from: https://doi.org/10.1016/j.chiabu.2022.105833
557 18.	Blanco M, Solano S, Alcántara AI, Parks M, Román FJ, Sepúlveda AR.
558 Psychological well-being and weight-related teasing in childhood obesity: a
559 case-control study. Eat Weight Disord. 2020 Jun;25(3):751–9.
560 19.	Şahın N, Kırlı U. The Relationship Between Peer Bullying and Anxiety-
561 Depression Levels in Children With Obesity. Anatol J Psychiatry [Internet].
562 2020; Available from: https://pubmed.ncbi.nlm.nih.gov/36425935
563 20.	Pryor L, Brendgen M, Boivin M, Dubois L, Japel C, Falissard B, et al.
564 Overweight during childhood and internalizing symptoms in early adolescence:
565 The mediating role of peer victimization and the desire to be thinner. J Affect
566 Disord [Internet]. 2016; Available from:
567 https://pubmed.ncbi.nlm.nih.gov/27267292
568 21.	Vuuren CL van, Wachter G, Veenstra R, Rijnhart JJM, Wal MF van der,
569 Chinapaw MJM, et al. Associations between overweight and mental health
570 problems among adolescents, and the mediating role of victimization. BMC
571 Public Health [Internet]. 2019; Available from:
572 https://pubmed.ncbi.nlm.nih.gov/31113424
573 22.	Rupp K, McCoy SM. Bullying Perpetration and Victimization among
574 Adolescents with Overweight and Obesity in a Nationally Representative
575 Sample. Child Obes [Internet]. 2019; Available from:
576 https://pubmed.ncbi.nlm.nih.gov/31062988
577 23.	Kozasa S, Oiji A, Kiyota A, Sawa T, Kim S-Y. Relationship between the
578 experience of being a bully/victim and mental health in preadolescence and
579 adolescence: a cross-sectional study. Ann Gen Psychiatry [Internet]. 2017;
580 Available from: https://pubmed.ncbi.nlm.nih.gov/29075310
581 24.	Wood MA. Friendship Security and Intimacy Moderate the Stability of Anxiety
582 During Early Adolescence. 2013.
583 25.	Adedeji A, Otto C, Kaman A, Reiss F, Devine J, Ravens-Sieberer U. Peer
584 Relationships and Depressive Symptoms Among Adolescents: Results From
585 the German BELLA Study. Front Psychol. 2022;12(January).
586 26.	Gorrese A. Peer Attachment and Youth Internalizing Problems: A Meta-
587 Analysis. Child Care Q. 2016;
588 27.	Zimmer‐Gembeck MJ, Clear SJ, Campbell SM. Peer relationships and stress:
589 Indirect associations of dispositional mindfulness with depression, anxiety and
590 loneliness via ways of coping. J Adolesc. 2021;
591 28.	Chiu K, Clark DM, Leigh E. Prospective associations between peer functioning
592 and social anxiety in adolescents: A systematic review and meta-analysis. J
593 Affect Disord. 2020;
594 29.	Lampard AM, Franckle RL, Davison KK. Maternal depression and childhood
595 obesity: A systematic review. Prev Med (Baltim). 2014;59(1):60–7.
596 30.	Foster BA, Weinstein K, Mojica CM, Davis MM. Parental Mental Health
597 Associated With Child Overweight and Obesity, Examined Within Rural and
598 Urban Settings, Stratified by Income. J Rural Heal Off J Am Rural Heal Assoc
599 Natl Rural Heal Care Assoc. 2020 Jan;36(1):27–37.
600 31.	Goodman SH, Rouse MH, Connell AM, Broth MR, Hall CM, Heyward D.
601 Maternal Depression and Child Psychopathology: A Meta-Analytic Review.
602 Clin Child Fam Psychol Rev [Internet]. 2011;14(1):1–27. Available from:
603 https://doi.org/10.1007/s10567-010-0080-1
604 32.	Wang L, Anderson JL, Dalton W, Wu T, Liu X, Zheng S, et al. Maternal
605 Depressive Symptoms and the Risk of Overweight in Their Children. Matern
606 Child Health J [Internet]. 2013; Available from:
607 https://pubmed.ncbi.nlm.nih.gov/22833333
608 33.	Hope S, Micali N, Deighton J, Law C. Maternal mental health at 5 years and
609 childhood overweight or obesity at 11 years: evidence from the UK Millennium
610 Cohort Study. Int J Obes [Internet]. 2019; Available from:
611 https://doi.org/10.1038/s41366-018-0252-5
612 34.	Connell AM, Goodman SH. The association between psychopathology in
613 fathers versus mothers and children’s internalizing and externalizing behavior
614 problems: a meta-analysis. Psychol Bull. 2002 Sep;128(5):746–73.
615 35.	Kluczniok D, Boedeker K, Hindi Attar C, Jaite C, Bierbaum A-L, Fuehrer D, et
616 al. Emotional availability in mothers with borderline personality disorder and
617 mothers with remitted major depression is differently associated with
618 psychopathology among school-aged children. J Affect Disord. 2018
619 Apr;231:63–73.
620 36.	Choi J. The Moderating Role of Family Resilience in the Relationship of
621 Bullying Victimization, Adverse Childhood Experiences, and Poverty Status to
622 Adolescents’ Internalizing Disorders. J Aggress Maltreat Trauma. 2022;
623 37.	Uddin J, Ahmmad Z, Uddin H, Tatch A. Family resilience and protective factors
624 promote flourishing and school engagement among US children amid
625 developmental disorder and adverse psychosocial exposure. Sociol Spectr.
626 2021;
627 38.	Zhuo R, Yu Y, Shi X. Family Resilience and Adolescent Mental Health during
628 COVID-19: A Moderated Mediation Model. Int J Environ Res Public Health.
629 2022;
630 39.	Ao L, Cheng X, An D, An Y, Yuan G. Relationship between Perceived Family
631 Resilience, Emotional Flexibility, and Anxiety Symptoms: a Parent-Adolescent
632 Dyadic Perspective. J Youth Adolesc. 2024;
633 40.	Bronfenbrenner U. Mental Health and Well-being: A Socio-Ecological Model
634 [Internet]. University of Minnesota School of Public Health. 2021. p. 1–2.
635 Available from:
636 https://[www.telethonkids.org.au/globalassets/media/documents/projects/sew---](http://www.telethonkids.org.au/globalassets/media/documents/projects/sew---)
637 arts/the-social-ecological-model-2.2021.pdf
638 41.	Golden SD, Earp JAL. Social Ecological Approaches to Individuals and Their
639 Contexts: Twenty Years of Health Education & Behavior Health Promotion
640 Interventions. Heal Educ Behav. 2012;39(3):364–72.
641 42.	Belsky J, Jonassaint C, Pluess M, Stanton M, Brummett B, Williams R.
642 Vulnerability genes or plasticity genes? Mol Psychiatry. 2009;14(8):746–54.
643 43.	Pierson C. The Social Environment, Genetics, And Obesity: Evaluation of
644 Diathesis-Stress and Differential Susceptibility Frameworks. 2013.
645 44.	Avila C, Holloway AC, Hahn MK, Morrison KM, Restivo M, Anglin R, et al. An
646 Overview of Links Between Obesity and Mental Health. Curr Obes Rep.
647 2015;4(3):303–10.
648 45.	Beltrán-Garrayo L, Solar M, Blanco M, Graell M, Sepúlveda AR. Examining
649 associations between obesity and mental health disorders from childhood to
650 adolescence: A case-control prospective study. Psychiatry Res.
651 2023;326(March).
652 46.	HHS. 2021-2022 National Survey of Children’s Health. 2024.
653 47.	Ebesutani C, Tottenham N, Chorpita B. The Revised Child Anxiety and
654 Depression Scale - Parent Version: Extended Applicability and Validity for Use
655 with Younger Youth and Children with Histories of Early-Life Caregiver
656 Neglect. J Psychopathol Behav Assesss. 2015;37(4):705–18.
657 48.	Lindberg L, Hagman E, Danielsson P, Marcus C, Persson M. Anxiety and
658 depression in children and adolescents with obesity: A nationwide study in
659 Sweden. BMC Med. 2020;18(1):1–9.
660 49.	Foody M, Samara M, O’Higgins Norman J. Bullying by siblings and peers:
661 Poly-setting victimization and the association with problem behaviours and
662 depression. Br J Educ Psychol. 2020 Jun;90 Suppl 1:138–57.
663 50.	Anderson JR, Mayes TL, Fuller A, Hughes JL, Minhajuddin A, Trivedi MH.
664 Experiencing bullying’s impact on adolescent depression and anxiety:
665 Mediating role of adolescent resilience. J Affect Disord. 2022 Aug;310:477–83.
666 51.	Lin L, Chien Y, Chen Y, Wu C, Chiou H. Bullying Experiences , Depression ,
667 and the Moderating Role of Resilience Among Adolescents. 2022;10(May):1–
668 11.

669	1.

670

671

672

673

674

675

676

Figure 1	[Click here to access/download;Figure;Plos - Figure 1.docx](https://www2.cloud.editorialmanager.com/pgph/download.aspx?id=625784&guid=ba0453e3-0d54-4997-a9ef-bfca7ce30648&scheme=1)[<https://www2.cloud.editorialmanager.com/pgph/download.aspx?id=625784&guid=ba0453e3-0d54-4997-a9ef-bfca7ce30648&scheme=1>](https://www2.cloud.editorialmanager.com/pgph/download.aspx?id=625784&guid=ba0453e3-0d54-4997-a9ef-bfca7ce30648&scheme=1)

*Figure 1: Predicted Probability of Depression - Bullying Category*

Figure 2	[Click here to access/download;Figure;Plos - Figure 2.docx](https://www2.cloud.editorialmanager.com/pgph/download.aspx?id=625785&guid=6d549836-fe02-4383-b2e8-723372f2bac3&scheme=1)[<https://www2.cloud.editorialmanager.com/pgph/download.aspx?id=625785&guid=6d549836-fe02-4383-b2e8-723372f2bac3&scheme=1>](https://www2.cloud.editorialmanager.com/pgph/download.aspx?id=625785&guid=6d549836-fe02-4383-b2e8-723372f2bac3&scheme=1)

*Figure 2: Predicted Probability of Depression - Mental health of Father*

Figure 3	[Click here to access/download;Figure;Plos - Figure 3.docx](https://www2.cloud.editorialmanager.com/pgph/download.aspx?id=625786&guid=5ee44294-d5e0-418f-9327-b555b77cb9da&scheme=1)[<https://www2.cloud.editorialmanager.com/pgph/download.aspx?id=625786&guid=5ee44294-d5e0-418f-9327-b555b77cb9da&scheme=1>](https://www2.cloud.editorialmanager.com/pgph/download.aspx?id=625786&guid=5ee44294-d5e0-418f-9327-b555b77cb9da&scheme=1)

*Figure 3: Predicted Probability of Depression - Mental Health of Mother*

Figure 4	[Click here to access/download;Figure;Plos - Figure 4.docx](https://www2.cloud.editorialmanager.com/pgph/download.aspx?id=625787&guid=f18481f1-5710-4f31-a982-8fb0ec72520a&scheme=1)[<https://www2.cloud.editorialmanager.com/pgph/download.aspx?id=625787&guid=f18481f1-5710-4f31-a982-8fb0ec72520a&scheme=1>](https://www2.cloud.editorialmanager.com/pgph/download.aspx?id=625787&guid=f18481f1-5710-4f31-a982-8fb0ec72520a&scheme=1)

*Figure 4: Predicted Probability of Anxiety - Making Friends*

Figure 5	[Click here to access/download;Figure;Plos - Figure 5.docx](https://www2.cloud.editorialmanager.com/pgph/download.aspx?id=625788&guid=1ddbff4c-937b-4506-a690-8674e4640f1a&scheme=1)[<https://www2.cloud.editorialmanager.com/pgph/download.aspx?id=625788&guid=1ddbff4c-937b-4506-a690-8674e4640f1a&scheme=1>](https://www2.cloud.editorialmanager.com/pgph/download.aspx?id=625788&guid=1ddbff4c-937b-4506-a690-8674e4640f1a&scheme=1)

*Figure 5: Predicted Probability of Anxiety - Father’s Mental Health*

Abstract

[Click here to access/download](https://www2.cloud.editorialmanager.com/pgph/download.aspx?id=626118&guid=eb4d97ed-4a83-4977-9bef-d0d60997a573&scheme=1)

[**Supporting Information** ABSTRACT_paper 1.docx](https://www2.cloud.editorialmanager.com/pgph/download.aspx?id=626118&guid=eb4d97ed-4a83-4977-9bef-d0d60997a573&scheme=1)
