# Supplementary material for: Between the scales and the scars: The interpersonal moderators of the association between Body Mass Index and parent-reported depression/anxiety diagnoses in U.S. children: Evidence from the 2021–22 National Survey of Children’s Health (NSCH)
Source: PLOS Glob Public Health. 2026 May 20;6(5):e0006462. doi: 10.1371/journal.pgph.0006462 (PMC13189307; doi:10.1371/journal.pgph.0006462)
Supplement: Attachment — Submitted filename: Response to Reviewers_PLOS.docx [file pgph.0006462.s006.docx]

**Manuscript Title:** Between the Scales and the Scars: The Interpersonal Burden of Body Weight on Children’s Mental Health: Evidence from the 2021–22 National Survey of Children’s Health (NSCH)
**Manuscript ID:** PGPH-D-25-02696
**Authors:** Fnu Kajal et al.
**Journal:** PLOS Global Public Health

Dear Editors and Reviewers,

We sincerely thank the reviewers for their thoughtful and constructive comments. We carefully revised the manuscript to address all the points raised. Below, we provide a detailed, point-by-point response, including specific line numbers indicating where changes were made in the revised manuscript.

**Reviewer 1 Comments and Author Responses**

**Introduction:**

The introduction does a good job of summarizing the literature, but the research gap is not clearly defined. It would be helpful to highlight that few studies have closely examined the interpersonal and social factors in the connection between obesity and mental health.

**Response:** We agree. We clarified the research gap by explicitly noting the absence of studies examining multiple interpersonal factors simultaneously in nationally representative datasets.
**Changes:** Lines 114-117, 130–133 and 142-148.

Some citation clusters feel too dense (e.g., lines 87–93). Integrating references more smoothly into the narrative would improve readability.

**Response:** The relevant section has been rewritten for improved narrative flow.
**Changes:** Lines 86-88.

The introduction concludes with bullying, which is a strong point, but it would be better to conclude with a clear lead-in to the study’s aims.

**Response:** Thank you for this suggestion. We revised the end of the Introduction to include a clear transition into the study aims and to explicitly state the purpose of the analysis, as well as the socio-ecological framing. The revised concluding paragraph now summarizes the key interpersonal variables and states how these inform the research objectives.

**Changes**: Lines 114-117.

The text switches between “children” and “adolescents.” Since the dataset covers the age range of 0–17, the authors should consider clarifying upfront what “children” means in this study.

**Response:** The study population defines the age group from 6-17 years.
**Changes:** Lines 185-186.

**Methods:**

Since all outcomes are parent-reported, there is a real risk of bias, especially for sensitive issues like mental health and bullying. Authors should address the validity of these reports and whether NSCH has validation data.

**Response:** We added a discussion of potential reporting bias and referenced NSCH validation documentation.
**Changes:** Lines 195-198.

Merging current and lifetime diagnoses of depression/anxiety into a single binary outcome could mask important differences. Authors should justify this decision or provide sensitivity analyses with separate categories.

**Response:** Thank you for this critical methodological observation. In the NSCH dataset, the depression and anxiety measures are structured as two-part questions indicating whether the child has **ever** been diagnosed and whether the condition is **current**. Given the relatively low prevalence of current diagnoses and the well-documented instability of episodic internalizing symptoms in youth, we used a combined lifetime indicator to ensure adequate statistical power and reduce sparse cell bias in stratified interaction models. This approach has been used in prior epidemiological analyses of NSCH mental health outcomes. Nonetheless, to address the reviewer’s concern, we have now added justification for this analytic decision in the Methods section.

**Changes**: Lines 204-211.

Parent-reported height/weight may misclassify BMI. This limitation should be acknowledged, ideally with references to validation work.

**Response:** We agree and have now explicitly acknowledged the potential for measurement misclassification due to parent-reported height and weight. We added references to validation studies indicating that while parent-reported values may slightly underestimate BMI, the overall accuracy is generally acceptable for population-level research using NSCH and similar national surveys.

**Changes:** Lines 195-198.

Moderators (bullying, friendships, parental mental health, resilience) are well-chosen, but more detail on how they were measured would be helpful. Were these scales validated?

**Response**: Thank you for this helpful suggestion. We have now expanded the Methods section to detail how each moderator variable was assessed in the NSCH. These measures are standardized surveillance items used consistently across federal child health monitoring systems. Although they are single-item indicators rather than multi-item psychometric scales, prior NSCH technical documentation indicates that these indicators have acceptable construct validity for population-level behavioural surveillance. We also clarified that these measures capture caregiver-reported perceptions and thus reflect the interpersonal context as experienced and interpreted within family environments.

**Changes**: Lines 228-232, 235-238, 241-244, and 249-251.

Excluding 23,715 cases is significant. Did excluded participants differ from included ones in key demographics or health factors? A brief comparison would strengthen confidence.

**Response:** We included a brief comparison showing no significant demographic differences between participants who were included and those who were excluded.

**Changes**: Lines 324-326.

The analytic approach is sound, but it needs further justification. Why quasibinomial instead of standard logistic regression? Were multicollinearity checks done? Were interactions pre-specified?

**Response**: We have expanded the Methods section to justify our analytic choices. We used quasibinomial logistic regression to account for mild overdispersion in binary outcome models, which is recommended in large population survey analyses when variance exceeds the binomial assumption. We also conducted multicollinearity diagnostics using variance inflation factors (VIF), which indicated no concerning collinearity among covariates. Finally, the interaction terms for bullying, friendship difficulty, parental mental health, and family resilience were pre-specified based on the socio-ecological theoretical framework. These details have now been added to the manuscript.

**Changes**: Lines 292-299.

Authors should strive to maintain consistent terminology (e.g., LDD/LDA). Currently, it alternates between full and shorthand labels.

**Response**: We have now standardized terminology for the mental health outcome variables across the manuscript. The terms **“lifetime diagnosis of depression (LDD)”** and **“lifetime diagnosis of anxiety (LDA)”** are each defined once at first mention, and the corresponding abbreviations are used consistently throughout the text, tables, and figure notes. We also reviewed the manuscript to ensure that no inconsistent or outdated labels remain.

**Changes**: Lines 206-211, and in relevant places in methods and results.

The explanation of FPL categories could be simplified, perhaps with a small table instead of dense text.

**Response:** We agree that the previous text-based description of Federal Poverty Level (FPL) categories was dense. We replaced this paragraph with a concise table that summarizes each FPL category and its corresponding household income meaning. This improves readability and clarity for the reader.

**Changes**: Lines 318.

**Results:**

Double-check formatting and numbers. For example, in Table 2, some ORs/CI ranges look off (e.g., “1.53(1.18-197)”).

**Response**: Thank you for bringing this to our attention. We audited all odds ratios and confidence intervals and corrected both **a numeric typo and formatting inconsistencies** in Table 2 (and mirrored the same standards in Tables 3 and 4). Specifically, the adjusted OR for **Overweight → Depression** was mistakenly rendered as 1.53(1.18–197) in the working Word file; this has been corrected to 1.53 (1.18–1.97). We also standardized spacing, symbols, and decimal precision across all tables. The corrected values and formatting are now reflected in the revised manuscript.

**Changes: Narrative text:** standardized “95%CI” → “95% CI” and spacing around “OR” (e.g., OR=1.20 → OR 1.20 or OR = 1.20, per journal style) in the Results paragraph preceding Table 2.

**Global formatting rules applied to all tables:**

1. Space before CIs: 1.20(1.01–1.43) → 1.20 (1.01–1.43),
2. En dash (–) in CI ranges,
3. Two-decimal precision for ORs and CI bounds,
4. Consistent “aOR” label in table body and notes,
5. “95% CI” spelled with a space.

The transitions between tables/figures and the narrative feel abrupt. Briefly summarizing the key takeaway after each would help readers follow along.

**Response**: We agree that adding brief interpretive transition sentences would improve readability. We have now added a concise summary statement after each major table summarizing the key takeaway before moving into the stratified results. These additions do not introduce new interpretation but enhance narrative continuity and flow.

**Changes**: Lines: 320-323, 325-327, 340-341, 370-373, 397-399, and 415-417.

With so many stratified analyses, the type I error risk rises. Were any corrections (Bonferroni, FDR) applied? If not, authors should consider acknowledging this.

**Response:** We added statements in both the Methods and Discussion sections acknowledging this and describing the stratified analyses as exploratory.
**Changes:** Lines 297-299, and 549-551.

For non-significant results, rather than just saying “not significant,” point to the CI overlap with the null to show strength of evidence.

**Response:** We now reference confidence intervals crossing the null instead of stating “not significant.”
**Changes:** Lines 352-356 and 444-449.

**Discussion:**

Some phrasing suggests causation when the design only supports associations. Use more cautious wording (e.g., “associated with” instead of “contributes to”).

**Response:** All causal language has been replaced with associational phrasing.
**Changes:** Lines 477-479 and other relevant places.

The explanation for the distinction between infrequent and frequent bullying is speculative. Authors should clearly frame it as a hypothesis, cite relevant resilience literature, and avoid overextending the claim.

**Response:** This explanation is now explicitly framed as a hypothesis, supported by resilience literature, and identified as requiring longitudinal confirmation.
**Changes:** Lines 485-492.

The emphasis on equity and multi-level interventions is strong, but it is essential to avoid overstating what the dataset itself supports. Policy recommendations should be framed as potential applications, not direct conclusions from the data.

**Response:** We revised to emphasize that proposed interventions are suggested implications rather than conclusions directly derived from causal evidence.
**Changes:** Lines 526-529.

**Also, the minor edits suggested by another reviewer (AJAO) are incorporated at relevant places:**

You don’t start Abstract with the independent variable, rather you start with the dependent variable (Children’s Mental Health).

**Response:** Agreed

**Changes:** Lines 45-48.

Please, clarify between the conclusion and recommendation(s) of the study.

**Response**: Clarified

**Changes**: Lines 67-72.

Please expound these “few studies” with supported and related literature.

**Response**: Added the relevant literature.

**Changes**: Lines 141.

There is no flow in your statement. Please try to restructure the last paragraph of your introduction to show the reason upon which the research was conducted. You ended the “Introduction” abruptly.

**Response**: Added a para for better flow

**Changes**: Lines 142-148.

Where? Please cite the supported literature in form of Parenthetical citation(s).

**Response**: Added citation to data source

**Changes:** Line 175.

Please use APA format for your Tables

**Response**: Made edits to all tables

**Changes**: All tables.

Please ensure that while you write the literatures that support the findings, there is also a need to cite literature that negates the findings too. Do this to all your findings narrative.

**Response:** Made edits to the discussion accordingly

**Changes**: Lines added 471-474, 500-503, 516-518.

Please follow 7^th^ Edition of APA referencing style in all your references

**Response**: Used Vancouver style as given under PLOS guidelines

**Changes**: No change made.

We are grateful to the reviewers for their insightful comments, which have strengthened the rigor, clarity, and interpretative balance of our manuscript. We believe the revised version is significantly improved and hope it will now meet the journal’s publication standards.

Thank you for your time and consideration.

Sincerely,

Fnu Kajal
