# Supplementary material for: Between the scales and the scars: The interpersonal moderators of the association between Body Mass Index and parent-reported depression/anxiety diagnoses in U.S. children: Evidence from the 2021–22 National Survey of Children’s Health (NSCH)
Source: PLOS Glob Public Health. 2026 May 20;6(5):e0006462. doi: 10.1371/journal.pgph.0006462 (PMC13189307; doi:10.1371/journal.pgph.0006462)
Supplement: Attachment — Submitted filename: Response to Reviewers_2nd revision.docx [file pgph.0006462.s008.docx]

**Manuscript Title:** Between the Scales and the Scars: The Interpersonal Moderators of the Association Between Body Mass Index and Parent-Reported Depression/Anxiety Diagnoses in U.S. Children: Evidence from the 2021- 22 National Survey of Children’s Health (NSCH)

**Manuscript ID:** PGPH-D-25-02696R1
**Journal:** PLOS Global Public Health

**Dear Editors and Reviewers,**

We sincerely thank the reviewers for their thoughtful and constructive feedback. We have carefully revised the manuscript to address all comments. As reflected in the review summary, the manuscript is now considered technically sound, and all major concerns have been addressed.

In this revision, we have:

- Strengthened the **methodological clarity**, including explicit discussion of parent-reported measures and common method bias
- Conducted and reported **sensitivity analyses** using current diagnoses of depression/anxiety
- Removed all **causal language** and ensured interpretations are strictly associational
- Provided **subgroup counts** and clarified interpretation of interaction findings
- Standardized **tables, figures, and statistical reporting**
- Incorporated **clear, evidence-based recommendations** to enhance the study’s contribution to policy and practice
- Improved the **clarity, structure, and consistency** of the manuscript throughout

We have also ensured that all editorial comments have been addressed and that the manuscript is presented in a clear, coherent, and publication-ready format. Below, we provide a concise, point-by-point response that indicates the specific changes and corresponding line numbers in the revised manuscript.

**Reviewer 1**

**Comment 1, 2 & 8:** The study relies entirely on data reported by parents for the independent variable (height and weight for Body Mass Index, or BMI), the dependent variable (mental health diagnoses), and the moderators (bullying and family resilience). This reliance poses a significant risk of common method variance. A parent with mental health issues may perceive or report their child as having problems, which could inflate the associations seen in the study. Although the authors recognize the potential for BMI misclassification, depending on one reporter for all the variables threatens the validity of the findings. The limitations section should be expanded to discuss Common Method Bias beyond just BMI misclassification. It is essential to consider how a parent's mental health status (which acts as a moderator) affects their reporting of the child's experiences with bullying and mental health conditions.

**Response:** We agree that reliance on parent-reported data across exposure, outcomes, and moderators introduces potential same-informant (common method) bias. We have strengthened the manuscript to explicitly acknowledge this limitation.

**Revisions made:** Expanded Methods to clarify that all primary variables are parent-reported. Added a detailed discussion on common method variance, including the possibility that parental mental health may influence reporting of child outcomes and exposures.

**Location:** Methods (Data Collection) and Discussion (Limitations)

**Changes:** Lines 203-216, 664-671

**Comment 3:**  The authors combined "Current" diagnoses with "Lifetime" diagnoses into one binary outcome to maintain sample size. This decision complicates a cross-sectional study that connects current BMI with mental health. A child may have received a diagnosis years ago (a lifetime diagnosis) but be in remission now, yet they are analyzed with their current BMI. This mismatch can confuse the results. For example, a child who was depressed three years ago, but is currently healthy and obese, contributes to a positive association that may not be causal in the present.

**Response**: We appreciate this important concern regarding temporal mismatch. We have clarified the rationale for combining current and lifetime diagnoses to preserve statistical power and reduce sparse data bias in interaction models.

To directly address this limitation, we conducted a **sensitivity analysis using only current diagnoses**.

**Revisions made:**

- Added justification for combining outcomes in Methods.
- Conducted and reported sensitivity analyses restricted to current depression and anxiety.
- Explicitly discussed temporal misalignment as a limitation.

**Location:** Methods (Outcomes), Results (Sensitivity analysis), Supplementary Table S2, Discussion

**Changes**: Lines 220-232, 380-397, 671-675

**Comment 4:** The results show a significant association between obesity and depression for children who experienced "never" or "occasional" bullying. However, the confidence interval for those who faced "frequent" bullying crosses the null. The authors suggest that frequent bullying might build resilience. This idea is controversial and could be dangerous, especially without long-term data to support it. It is also likely that a smaller group size contributed to this finding, or that frequently bullied children show symptoms not measured by the specific anxiety and depression assessments (like acting out or complete withdrawal).

**Response:** We agree with the reviewer and have **removed the interpretation suggesting resilience**.

**Revisions made:**

- Eliminated all statements implying that frequent bullying may build resilience.
- Replaced with a statistical explanation highlighting small subgroup size and reduced precision.

**Location:** Results and Discussion

**Changes**: Lines 450-459, 675-677

**Comment 5:** Even though this study is cross-sectional, the manuscript sometimes uses causal language. Phrases like "impact of," "escalate the risk," and "disrupt the pathways" suggest a directionality that cannot be established with this data. Reverse causation (for example, depression leading to obesity) is also a valid possibility, as noted in the introduction but not fully addressed in the conclusion

**Response:** We have revised the manuscript to ensure that all interpretations are **strictly associational** and consistent with a cross-sectional design.

**Revisions made:**

- Replaced causal phrases (e.g., “impact,” “escalate risk,” “disrupt pathways”) with non-causal language such as “associated with” and “may reflect.”
- Added explicit acknowledgment of potential reverse causation in the Discussion.

**Location:** The whole manuscript was screened to correct this anomaly.

**Comment 6**: The authors should conduct a sensitivity analysis that separates "Current" diagnoses from "Lifetime" diagnoses. Even if the sample size is smaller, it is crucial to show the odds ratios for Current Depression/Anxiety associations with Current BMI. If the "Current" model does not show significance, this limitation must be clearly stated.

**Response:** As recommended, we conducted sensitivity analyses restricted to current diagnoses.

**Findings:**

- Associations remained directionally consistent but were attenuated and no longer statistically significant.
- This likely reflects reduced statistical power due to a smaller sample size.

**Revisions made:**

- Added Supplementary Table S2.
- Added Results paragraph summarizing findings.
- Interpreted results cautiously in the Discussion.

**Changes:** Lines 220-232, 380-397, 671-675

**Comment 7:** The authors should provide the raw counts (both unweighted and weighted) for the "Obese + Frequent Bullying" category. If this sample size is small, the lack of significance (CI crossing 1.0) is likely due to a Type II error (a power issue) rather than a "resilience" effect. The discussion on "frequent bullying building resilience" should be removed or carefully qualified as a statistical artifact unless strong literature supports this claim.

**Response:** We have provided both unweighted and weighted counts for this subgroup.

**Findings:**

- The obese + frequent bullying subgroup comprised **n = 399 children (0.95% of the analytic sample)**.

**Interpretation:**

- The small subgroup size likely reduced precision and explains the confidence interval crossing the null (Type II error), rather than indicating a true protective effect.

**Revisions made:**

- Added Supplementary Table S3 with full cell counts.
- Reported subgroup size explicitly in Results.
- Revised Discussion accordingly.

**Changes**: Lines: 450-459, 675-677.

**Reviewer 2**

**Comment**: I read the manuscript with great care and attention. The authors have satisfactorily addressed the questions posed in the initial submission. The manuscript is now more thorough and easier to read. It is now more appropriate technically. The manuscript may now contribute to the body of scientific literature in its current form, in my opinion.

**Response:** We sincerely thank the reviewer for their thorough re-evaluation of the manuscript and for the encouraging feedback. We are glad that the revisions have enhanced the clarity, rigor, and technical correctness of our work. We appreciate the reviewer’s acknowledgment of the manuscript’s contribution and are dedicated to upholding these standards in the final version.

**Reviewer 3**

**Comment**: Let the Authors endeavour to implement all the corrections before further submission please.

**Response:** We confirm that all reviewer comments have been thoroughly addressed and incorporated, and the manuscript has been carefully revised to ensure clarity, consistency, and completeness.

**Reviewer 4**

Very nice work. I have a few comments:

**Comment 1**. Line 48 - The LDA/LDD acronyms are appearing for the first time, you should spell it out just like you did for the BMI in line 54.

**Response:** We thank the reviewer for highlighting this oversight. We have now defined both acronyms at first mention in the Abstract.

**Revisions made:**

- “Parent-reported lifetime diagnosis of depression (LDD)” and “parent-reported lifetime diagnosis of anxiety (LDA)” are now spelled out at first use.

**Location:** Abstract and Introduction.

**Changes**: Line 50-51, 95 & 97.

**Comment 2.** In your results section of the abstract, you should qualify your moderators. For example in line 62, you report maternal mental health when you are actually reporting the poor maternal mental health. The same goes for poor paternal mental health and lots of difficulty making friends.

**Response:** We agree and have revised the Abstract to accurately qualify the moderators.

**Revisions made:**

- Updated wording to explicitly reflect:
  - poor maternal mental health
  - poor paternal mental health
  - A greater difficulty in making friends
- Ensured consistency with variable definitions in Methods.

**Location:** Abstract

**Changes**: Lines 55-59.

**Comment 3**: Line 155 - You use SEM for the first time. While I know you are referring to the socioecological model, I think you should still spell it out.

**Response:** We thank the reviewer for noting this. The term has now been spelled out at first use.

**Revisions made:**

- Revised to “socio-ecological model (SEM)” at first mention.

**Location:** Theoretical Framework

**Changes**: Line 159.

**Comment** 4: Between lines 325 and 327, you conclude that the supplementary table demonstrates there were no significant demographic differences between participants who were included and those who were excluded. I beg to differ. I can see some considerable differences, especially for the FPL (bar the 200-399% sub-category) and the Race/Ethnicity category (bar the other sub-category). Unless you performed a statistical significance analysis showing that these differences are not significant, I really do not see the utility of this table.

**Response:** We appreciate this important clarification. We agree that the previous statement was inaccurate.

**Revisions made:**

- Removed the statement indicating “no significant differences.”
- Revised the text to reflect that:
  - **Age and sex distributions were similar**, but
  - **differences were observed in race/ethnicity, federal poverty level (FPL), and BMI category**
- Included results of survey-weighted statistical comparisons to support this interpretation.

**Location:** Results, Supplementary Table S1

**Changes**: Lines 353-365.

**Comment 5**. In line 338 and 339, I see you did not adjust for FPL for the LDA but did for the LDD. I am curious about this choice.

**Response:** We thank the reviewer for this important methodological query.

**Revisions made:**

- Added clarification that covariate inclusion was guided by both theoretical considerations and model fit.
- Federal Poverty Level (FPL) was retained in the LDD model due to its stronger association with depression outcomes and contribution to model fit, while its inclusion in the LDA model did not materially improve model performance.
- This rationale is now explicitly stated.

**Location:** Methods – Statistical Analysis.

**Changes**: Lines 314-316.

**Comment 6:** You mentioned in line 488, 'backed by resilience research' but did not include any source.

**Response:** We thank the reviewer for identifying this concern. Upon careful consideration, we have removed the interpretation that suggested a resilience effect in the frequent-bullying subgroup, as it was not sufficiently supported by the data.

**Revisions made:**

- Deleted statements implying a resilience effect.
- Reframed the findings to note that the association in the frequent bullying subgroup was attenuated and not statistically significant, likely due to limited sample size and reduced statistical power.
- Clarified that the absence of statistical significance should not be interpreted as the absence of an association.

**Location:** Results and Discussion

**Changes**: Lines: 450-459, 675-677.

**Comment 7:**Between lines 505 and 507, you attempt to explain the reason for the strong link between poor maternal mental health and child depression but offer none for the strong link between poor paternal mental health and child depression. I was wondering what you have to say about that.

**Response:** We thank the reviewer for this insightful observation. We agree that the manuscript should address the association between paternal mental health and child depression, alongside maternal mental health.

**Revisions made:**

- Added a brief discussion acknowledging that poor paternal mental health may also influence child mental health through pathways such as household emotional climate, parenting behaviors, and family functioning.
- Framed this interpretation cautiously, noting that evidence on paternal influences is comparatively less extensive and warrants further investigation.

**Location:** Discussion

**Changes:** Lines 613-622.

**Figure & Table Resubmissions:**

**Comment**: While revising your submission, we strongly recommend that you use PLOS’s NAAS tool (https://ngplosjournals.pagemajik.ai/artanalysis) to test your figure files. NAAS can convert your figure files to the TIFF file type and meet basic requirements (such as print size, resolution), or provide you with a report on issues that do not meet our requirements and that NAAS cannot fix.

**Response:** We appreciate the editor's recommendation. All figure files have been reviewed and standardized using the PLOS NAAS tool to ensure compliance with the required specifications for format, resolution, and print size. Corrections have been made accordingly. Moreover, in response to reviewer comments, all tables have been standardized for numerical consistency, with percentages reported to two decimal places and p-values to three decimal places. We have confirmed that all figures and tables now adhere to PLOS submission requirements.

We believe that the revised manuscript makes a meaningful contribution to understanding the interpersonal context of childhood obesity and mental health and offers actionable insights for public health research and practice.

We respectfully request that the manuscript be considered for publication in **PLOS Global Public Health**.

Thank you again for your time, guidance, and consideration.

Sincerely,

Dr. Fnu Kajal

(On behalf of all authors)
