# Supplementary material for: Between the scales and the scars: The interpersonal moderators of the association between Body Mass Index and parent-reported depression/anxiety diagnoses in U.S. children: Evidence from the 2021–22 National Survey of Children’s Health (NSCH)
Source: PLOS Glob Public Health. 2026 May 20;6(5):e0006462. doi: 10.1371/journal.pgph.0006462 (PMC13189307; doi:10.1371/journal.pgph.0006462)
Supplement: Attachment — Submitted filename: Reviewer Comments.docx [file pgph.0006462.s005.docx]

**Reviewer Comments**

**Manuscript Title:** *Between the Scales and the Scars: The Interpersonal Burden of Body Weight on Children’s Mental Health: Evidence from the 2021–22 National Survey of Children’s Health (NSCH)*

**Overall Assessment**

This manuscript addresses a crucial and timely issue: the connection between childhood obesity and mental health, presented from a fresh angle on the role of interpersonal factors. The introduction is engaging, but the knowledge gap and rationale for the study could be spelled out more clearly. The methods are solid overall, with a large, nationally representative dataset and appropriate analytic techniques. However, the paper would benefit from greater clarity regarding how measures were validated, why confident analytic choices were made, and the limitations of relying on self-reports.

The results are thorough, well-presented, and enriched by examining interpersonal moderators; however, the flow could be smoother, and the tables require careful proofreading. The discussion effectively ties the findings to the literature and outlines practical implications, but some interpretations appear overly strong, given the cross-sectional design. A more cautious framing, closer alignment with existing research, and a more balanced perspective on parental roles would be beneficial. Overall, this is a strong and valuable study that needs moderate revisions before it is ready for publication.

**Points for Improvement**

**Introduction**

- The introduction does a good job of summarizing the literature, but the research gap is not clearly defined. It would be helpful to highlight that few studies have closely examined the interpersonal and social factors in the obesity–mental health connection.
- Some citation clusters feel too dense (e.g., lines 87–93). Integrating references more smoothly into the narrative would improve readability.
- The introduction concludes with bullying, which is a strong point, but it would be better to conclude with a clear lead-in to the study’s aims.
- The text switches between “children” and “adolescents.” Since the dataset covers the age range of 0–17, the authors should consider clarifying upfront what “children” means in this study.

**Methods**

- Since all outcomes are parent-reported, there is a real risk of bias, especially for sensitive issues like mental health and bullying. Authors should address the validity of these reports and whether NSCH has validation data.
- Merging current and lifetime diagnoses of depression/anxiety into a single binary outcome could mask important differences. Authors should justify this decision or provide sensitivity analyses with separate categories.
- Parent-reported height/weight may misclassify BMI. This limitation should be acknowledged, ideally with references to validation work.
- Moderators (bullying, friendships, parental mental health, resilience) are well-chosen, but more detail on how they were measured would be helpful. Were these scales validated?
- Excluding 23,715 cases is significant. Did excluded participants differ from included ones in key demographics or health factors? A brief comparison would strengthen confidence.
- The analytic approach is sound, but it needs further justification. Why quasibinomial instead of standard logistic regression? Were multicollinearity checks done? Were interactions pre-specified?
- Authors should strive to maintain consistent terminology (e.g., LDD/LDA). Currently, it alternates between full and shorthand labels.
- The explanation of FPL categories could be simplified, perhaps with a small table instead of dense text.

**Results**

- Double-check formatting and numbers. For example, in Table 2, some ORs/CI ranges look off (e.g., “1.53(1.18-197)”).
- The transitions between tables/figures and the narrative feel abrupt. Briefly summarizing the key takeaway after each would help readers follow along.
- With so many stratified analyses, the type I error risk rises. Were any corrections (Bonferroni, FDR) applied? If not, authors should consider acknowledging this.
- For non-significant results, rather than just saying “not significant,” point to the CI overlap with the null to show strength of evidence.

**Discussion & Conclusion**

- Some phrasing suggests causation when the design only supports associations. Use more cautious wording (e.g., “associated with” instead of “contributes to”).
- The explanation for the distinction between infrequent and frequent bullying is speculative. Authors should clearly frame it as a hypothesis, cite relevant resilience literature, and avoid overextending the claim.
- The emphasis on equity and multi-level interventions is strong, but it is essential to avoid overstating what the dataset itself supports. Policy recommendations should be framed as potential applications, not direct conclusions from the data.

**Recommendations for Improvement**

To strengthen the manuscript, the introduction would benefit from clearly stating the research gap upfront and maintaining consistent terminology (for example, using “children” vs. “adolescents”). In the methods, it would be helpful to be more transparent about how measures were validated, why confident analytic choices were made, and how self-reported data were handled. The results section could be tightened with careful proofreading of tables, a smoother narrative flow, and a note about the risk of multiple testing. In the discussion, the authors should use more cautious language, given the cross-sectional design, and flag speculative interpretations (such as the bullying paradox) as hypotheses to be explored further. Lastly, the policy implications are important, but they should be framed as possible applications of the findings rather than firm conclusions from the data.
