# Supplementary material for: Between the scales and the scars: The interpersonal moderators of the association between Body Mass Index and parent-reported depression/anxiety diagnoses in U.S. children: Evidence from the 2021–22 National Survey of Children’s Health (NSCH)
Source: PLOS Glob Public Health. 2026 May 20;6(5):e0006462. doi: 10.1371/journal.pgph.0006462 (PMC13189307; doi:10.1371/journal.pgph.0006462)
Supplement: Peer review history

# Peer review: Decision Letter 0

**DOI:** 10.1371/journal.pgph.0006462.r001

**Authors:** Feten Fekih-Romdhane

6 Oct 2025

PGPH-D-25-02696

Between the Scales and the Scars: The Interpersonal Burden of Body Weight on Children’s Mental Health: Evidence from the 2021- 22 National Survey of Children’s Health (NSCH)

PLOS Global Public Health

Dear Dr. Kajal,

Thank you for submitting your manuscript to PLOS Global Public Health. After careful consideration, we feel that it has merit but does not fully meet PLOS Global Public Health’s publication criteria as it currently stands. Therefore, we invite you to submit a revised version of the manuscript that addresses the points raised during the review process.

Please submit your revised manuscript by Nov 20 2025 11:59PM. If you will need more time than this to complete your revisions, please reply to this message or contact the journal office at globalpubhealth@plos.org. When you're ready to submit your revision, log on to https://www.editorialmanager.com/pgph/ and select the 'Submissions Needing Revision' folder to locate your manuscript file.

Please include the following items when submitting your revised manuscript:

- A rebuttal letter that responds to each point raised by the editor and reviewer(s). You should upload this letter as a separate file labeled 'Response to Reviewers'.
- A marked-up copy of your manuscript that highlights changes made to the original version. You should upload this as a separate file labeled 'Revised Manuscript with Track Changes'.
- An unmarked version of your revised paper without tracked changes. You should upload this as a separate file labeled 'Manuscript'.

Guidelines for resubmitting your figure files are available below the reviewer comments at the end of this letter.

We look forward to receiving your revised manuscript.

Kind regards,

Feten Fekih-Romdhane

Academic Editor

PLOS Global Public Health

Journal Requirements:

1. Please send a completed 'Competing Interests' statement, including any COIs declared by your co-authors. If you have no competing interests to declare, please state "The authors have declared that no competing interests exist".
2. Please provide separate figure files in .tif or .eps format.
  For more information about figure files please see our guidelines:
https://journals.plos.org/globalpublichealth/s/figures
https://journals.plos.org/globalpublichealth/s/figures#loc-file-requirements

3. If the reviewer comments include a recommendation to cite specific previously published works, please review and evaluate these publications to determine whether they are relevant and should be cited. There is no requirement to cite these works unless the editor has indicated otherwise.

Additional Editor Comments (if provided):

[Note: HTML markup is below. Please do not edit.]

Reviewers' comments:

Reviewer's Responses to Questions

**Comments to the Author**

1. Does this manuscript meet PLOS Global Public Health’s publication criteria? Is the manuscript technically sound, and do the data support the conclusions? The manuscript must describe methodologically and ethically rigorous research with conclusions that are appropriately drawn based on the data presented.

Reviewer #1: Yes

Reviewer #2: Yes

Reviewer #3: Yes

Reviewer #4: Partly

Reviewer #5: Yes

**********

2. Has the statistical analysis been performed appropriately and rigorously?

Reviewer #1: Yes

Reviewer #2: Yes

Reviewer #3: Yes

Reviewer #4: Yes

Reviewer #5: Yes

**********

3. Have the authors made all data underlying the findings in their manuscript fully available (please refer to the Data Availability Statement at the start of the manuscript PDF file)?

The PLOS Data policy requires authors to make all data underlying the findings described in their manuscript fully available without restriction, with rare exception. The data should be provided as part of the manuscript or its supporting information, or deposited to a public repository. For example, in addition to summary statistics, the data points behind means, medians and variance measures should be available. If there are restrictions on publicly sharing data—e.g. participant privacy or use of data from a third party—those must be specified.

Reviewer #1: Yes

Reviewer #2: Yes

Reviewer #3: Yes

Reviewer #4: Yes

Reviewer #5: Yes

**********

4. Is the manuscript presented in an intelligible fashion and written in standard English?

PLOS Global Public Health does not copyedit accepted manuscripts, so the language in submitted articles must be clear, correct, and unambiguous. Any typographical or grammatical errors should be corrected at revision, so please note any specific errors here.

Reviewer #1: Yes

Reviewer #2: Yes

Reviewer #3: Yes

Reviewer #4: Yes

Reviewer #5: Yes

**********

5. Review Comments to the Author

Please use the space provided to explain your answers to the questions above. You may also include additional comments for the author, including concerns about dual publication, research ethics, or publication ethics. (Please upload your review as an attachment if it exceeds 20,000 characters)

Reviewer #1: 1. The RAG enhancement's limited effectiveness on complex questions is a key finding, but more could be discussed as to why this would be the case in the paper. Is it something inherent in the chunking mechanism of the RAG system, the particular knowledge base chosen, or otherwise?

2. The explanation need to explained on performance of medical-specialized models like Meditron-70B (52.9% accuracy) is noted but not elaborately explained. The passing reference to potential causes for it, like a restricted training horizon or lack of advanced reasoning capabilities, would enrich the analysis.

3. The authors should elaborate more on the specific limitation of their RAG implementation. They mention that the RAG system was provided with a specific neurology textbook and that this could be different from the MedQA dataset in general, but this might be emphasized more in the discussion. A brief reference to other RAG strategies, i.e., multi-document RAG or differences in chunking, would demonstrate better insight into the trade-offs.

4. To extend the argument of the paper on the advantages of advanced AI structures towards solving complex medical reasoning issues, include a citation discussing the application of machine learning models towards the diagnosis of diseases. This would serve as a counterexample analogous to the paper's focus on neurological reasoning in underscoring the contribution of AI to increasing diagnostic precision in another field of medicine, for instance, cardiovascular disease. It is suggested to refer the paper: “Artificial intelligence in improving disease diagnosis. Artificial Intelligence in Medicine and Healthcare”. "A. Pati et al., “Artificial Intelligence in improving disease diagnosis,” Artificial Intelligence in Medicine and Healthcare, pp. 24–49, Jan. 2025. " Here is a two-stage experiment with the use of ML and DL algorithms for diagnosis of cardiovascular disease and high accuracy of 97.06%, which is a good, representative example of the ability of AI to improve diagnostic accuracy. My suggestion is to include this reference in the introduction or discussion, perhaps in the paragraph on recent advances in LLMs for medical reasoning

5. Clarifying Medical Model Weakness there is a need to expand discussion section to hypothesize why expert models like Meditron-70B fared badly. For instance, do they suffer from an inability to manage the "reasoning complexity" aspect despite having depth in factual knowledge? This would contribute immensely to the background of other researchers

6. I recommend that the authors revise the recommendation changes and suggestions to add and proceed with the further review process for publication.

Reviewer #2: The purpose of this study is to investigate the influence of less studied interpersonal dynamics on the relationship between childhood obesity and lifetime diagnoses of anxiety and depression. My observations are given below:

(1) The study title is okay.

(2) The abstract needs minor revision. Although the abstract provides a clear overview of the background, methods, results, and conclusions, it is too detailed, particularly when it comes to reporting statistics, which makes it harder to read. A wider audience would benefit from increased clarity, accessibility, and overall impact if numerical details were simplified and implications were emphasized. Please add relevant keywords after the abstract.

(3) The introduction also needs minor revision. The introduction is thorough, organized effectively, and backed up by a wealth of empirical data. In addition to highlighting the reciprocal relationship between obesity and mental health, it places familial and interpersonal factors in the context of important but little-studied moderators. A solid conceptual foundation is provided by the combination of the diathesis-stress and socio-ecological frameworks. The section could be shorter, though, as the extensive prevalence data and high citation density run the risk of overshadowing the main research gap. Clarity and focus would be strengthened by a more concise statement of the study's distinctive contribution.

(4) The methods section also needs improvements. The methods section is comprehensive and offers precise information on the measures, data sources, and analytical techniques. Generalizability is improved by using a sizable, nationally representative dataset, and the weighting approach is clearly described. Concerns regarding reporting bias are raised by the use of parent-reported measures, especially for delicate variables like mental health. It should be stressed more that causal inference is limited by the cross-sectional design. Furthermore, the rationale behind the moderators' selection could be more explicit, even though they are clearly defined. The methodological rigor is praiseworthy overall.

(5) The results section also needs revision. With a clear display of prevalence estimates, odds ratios, and stratified findings, the results section offers a thorough and in-depth explanation of both descriptive and inferential analyses. Transparency is improved through the use of tables and figures. Nevertheless, the section is overly lengthy and detailed, making it challenging for readers to summarize the key conclusions. Clarity would be enhanced by more synthesis and attention to the most important findings. To improve interpretation, effect sizes should also be examined in terms of their practical significance rather than just their statistical significance.

(6) The discussion section also needs revision. The discussion is comprehensive, theoretically grounded, and connects findings with socio-ecological models, but it tends toward overextension in some explanations (e.g., bullying paradox, maternal caregiving roles), which could benefit from more cautious interpretation. While strengths include nuanced consideration of resilience and policy implications, the section is somewhat repetitive and could be more concise. Limitations are appropriately acknowledged, but a clearer integration of unexpected findings with prior evidence would strengthen the contribution. Greater focus on novel insights would enhance clarity and impact.

(7) The references are ok. Please correct it by following the journal’s guidelines. The tables and figures should be prepared following standard guidelines.

Reviewer #3: This is a good research work that actually contributes to Knowledge if only those corrections can be effected and attended to accordingly. Please attend to all the corrections and submit for further review. Thanks

Reviewer #4: Insightful findings and discussions, especially about the moderating effect of occasional bullying on depression. I have the following comments for the authors:

1. Your results section in the abstract should be rewritten for clarity, particularly lines 61 to 67. It is currently framed in a way that makes one assume that all levels of parental mental health have the reported effect in the abstract, but this is not so. A way you could rewrite this is to say: among children who were never bullied (aOR, CI:), had poor paternal (aOR, CI:) and maternal (aOR, CI:) mental health, those with obesity had significantly higher odds for depression.

2. I know you treated the interpersonal factors as moderators between obesity and depression/anxiety. But I think some of your data might be suggesting obesity is the moderator, for instance, amplifying the effect of bullying on depression. Consider Table 3.2, where in the never bullied group, those who are obese have the highest aOR for LDD. I think it might be important to address/acknowledge this in your discussion of findings. While you focused on why occasional bullying might produce higher odds for LDD compared to frequent bullying, I noticed you did not really touch on the subject of why the never-bullied group had an aOR of 2.12. The authors may wish to reconsider and reframe their interpretation of moderation accordingly.

3. In lines 159-160, you say ‘…the present study WILL EXPLORE how…’. This makes your study sound like a proposal rather than a study that has been conducted. I would rewrite this to say ‘…the present study EXPLORES how…’.

4. In lines 205-207, you mentioned that you combined current depression/anxiety and those who had ever been diagnosed as one category into one variable - LDD. Could you please provide your justification for this combination? I worry this could confound your results.

5. In Table 2, you have two figures (1.53 and 2.08) and their CI highlighted in red. Is there a reason for this? Also, the table states that FPL was not adjusted for in the analysis for anxiety, and I wonder if there was any reason for this.

6. You should revise the labeling of Table 3, as all the tables under it (3.1 to 3.3) show aOR. None of them shows an unadjusted OR.

7. In Table 3.3, the ‘bullying frequency’ column has some information that look like codes in parentheses, and I am not sure what their relevance for inclusion is.

8. In lines 322 to 324, you state that “Similarly, Figures 1, 2, and 3 presented indicate that children with a history of frequent bullying exhibited significantly greater predicted probabilities of depression across all BMI ranges, particularly among children who were overweight or obese”. First, figures 2 and 3 do not report on the effect of bullying. Second, the wide confidence intervals of some of your predicted probabilities raise concerns about the precision of your predicted probabilities, especially the green bars. Please address this.

Reviewer #5: This manuscript addresses a crucial and timely issue: the connection between childhood obesity and mental health, presented from a fresh angle on the role of interpersonal factors. The introduction is engaging, but the knowledge gap and rationale for the study could be spelled out more clearly. The methods are solid overall, with a large, nationally representative dataset and appropriate analytic techniques. However, the paper would benefit from greater clarity regarding how measures were validated, why confident analytic choices were made, and the limitations of relying on self-reports.

**********

6. PLOS authors have the option to publish the peer review history of their article (what does this mean?). If published, this will include your full peer review and any attached files.

**Do you want your identity to be public for this peer review?** If you choose “no”, your identity will remain anonymous but your review may still be made public.

For information about this choice, including consent withdrawal, please see our Privacy Policy.

Reviewer #1: No

Reviewer #2: **Yes:**Gyanesh Kumar Tiwari

Reviewer #3: No

Reviewer #4: No

Reviewer #5: No

**********

[NOTE: If reviewer comments were submitted as an attachment file, they will be attached to this email and accessible via the submission site. Please log into your account, locate the manuscript record, and check for the action link "View Attachments". If this link does not appear, there are no attachment files.]

-->Figure Resubmissions:

While revising your submission, we strongly recommend that you use PLOS’s NAAS tool (https://ngplosjournals.pagemajik.ai/artanalysis) to test your figure files. NAAS can convert your figure files to the TIFF file type and meet basic requirements (such as print size, resolution), or provide you with a report on issues that do not meet our requirements and that NAAS cannot fix.

After uploading your figures to PLOS’s NAAS tool - https://ngplosjournals.pagemajik.ai/artanalysis, NAAS will process the files provided and display the results in the "Uploaded Files" section of the page as the processing is complete. If the uploaded figures meet our requirements (or NAAS is able to fix the files to meet our requirements), the figure will be marked as "fixed" above. If NAAS is unable to fix the files, a red "failed" label will appear above. When NAAS has confirmed that the figure files meet our requirements, please download the file via the download option, and include these NAAS processed figure files when submitting your revised manuscript.

Attachment Submitted filename: REVIEWED ARTICLE-PROF AJAO.docx

Attachment Submitted filename: Reviewer Comments.docx

# Author comment: Author response to Decision Letter 1

**DOI:** 10.1371/journal.pgph.0006462.r002

14 Dec 2025

Attachment Submitted filename: Response to Reviewers_PLOS.docx

# Peer review: Decision Letter 1

**DOI:** 10.1371/journal.pgph.0006462.r003

**Authors:** Emma Campbell

4 Feb 2026

PGPH-D-25-02696R1

Between the Scales and the Scars: The Interpersonal Burden of Body Weight on Children’s Mental Health: Evidence from the 2021- 22 National Survey of Children’s Health (NSCH)

PLOS Global Public Health

Dear Dr. Kajal,

Thank you for submitting your manuscript to PLOS Global Public Health. After careful consideration, we feel that it has merit but does not fully meet PLOS Global Public Health’s publication criteria as it currently stands. Therefore, we invite you to submit a revised version of the manuscript that addresses the points raised during the review process.

The revised manuscript has been further assessed by the reviewers and their comments are below or in the attached file. Please review their comments and make the appropriate additional revisions they have requested.

Please submit your revised manuscript by Mar 21 2026 11:59PM. If you will need more time than this to complete your revisions, please reply to this message or contact the journal office at globalpubhealth@plos.org. When you're ready to submit your revision, log on to https://www.editorialmanager.com/pgph/ and select the 'Submissions Needing Revision' folder to locate your manuscript file.

Please include the following items when submitting your revised manuscript:

- A letter that responds to each point raised by the editor and reviewer(s). You should upload this letter as a separate file labeled 'Response to Reviewers'.
- A marked-up copy of your manuscript that highlights changes made to the original version. You should upload this as a separate file labeled 'Revised Manuscript with Track Changes'.
- An unmarked version of your revised paper without tracked changes. You should upload this as a separate file labeled 'Manuscript'.

Guidelines for resubmitting your figure files are available below the reviewer comments at the end of this letter.

We look forward to receiving your revised manuscript.

Kind regards,

Emma Campbell, Ph.D

Staff Editor

PLOS Global Public Health

Journal Requirements:

If the reviewer comments include a recommendation to cite specific previously published works, please review and evaluate these publications to determine whether they are relevant and should be cited. There is no requirement to cite these works unless the editor has indicated otherwise.

Additional Editor Comments (if provided):

[Note: HTML markup is below. Please do not edit.]

Reviewers' comments:

Reviewer's Responses to Questions

**Comments to the Author**

1. If the authors have adequately addressed your comments raised in a previous round of review and you feel that this manuscript is now acceptable for publication, you may indicate that here to bypass the “Comments to the Author” section, enter your conflict of interest statement in the “Confidential to Editor” section, and submit your "Accept" recommendation.

Reviewer #1: All comments have been addressed

Reviewer #2: All comments have been addressed

Reviewer #3: (No Response)

Reviewer #4: (No Response)

**********

2. Does this manuscript meet PLOS Global Public Health’s publication criteria? Is the manuscript technically sound, and do the data support the conclusions? The manuscript must describe methodologically and ethically rigorous research with conclusions that are appropriately drawn based on the data presented.

Reviewer #1: Yes

Reviewer #2: Yes

Reviewer #3: Yes

Reviewer #4: (No Response)

**********

3. Has the statistical analysis been performed appropriately and rigorously?

Reviewer #1: N/A

Reviewer #2: Yes

Reviewer #3: Yes

Reviewer #4: (No Response)

**********

4. Have the authors made all data underlying the findings in their manuscript fully available (please refer to the Data Availability Statement at the start of the manuscript PDF file)?

The PLOS Data policy requires authors to make all data underlying the findings described in their manuscript fully available without restriction, with rare exception. The data should be provided as part of the manuscript or its supporting information, or deposited to a public repository. For example, in addition to summary statistics, the data points behind means, medians and variance measures should be available. If there are restrictions on publicly sharing data—e.g. participant privacy or use of data from a third party—those must be specified.

Reviewer #1: Yes

Reviewer #2: Yes

Reviewer #3: Yes

Reviewer #4: (No Response)

**********

5. Is the manuscript presented in an intelligible fashion and written in standard English?

PLOS Global Public Health does not copyedit accepted manuscripts, so the language in submitted articles must be clear, correct, and unambiguous. Any typographical or grammatical errors should be corrected at revision, so please note any specific errors here.

Reviewer #1: Yes

Reviewer #2: Yes

Reviewer #3: Yes

Reviewer #4: (No Response)

**********

6. Review Comments to the Author

Please use the space provided to explain your answers to the questions above. You may also include additional comments for the author, including concerns about dual publication, research ethics, or publication ethics. (Please upload your review as an attachment if it exceeds 20,000 characters)

Reviewer #1: 1. The study relies completely on data reported by parents for the independent variable (height and weight for Body Mass Index or BMI), the dependent variables (mental health diagnoses), and the moderators (bullying and family resilience).

2. This reliance poses a significant risk of common method variance. A parent with mental health issues may perceive or report their child as having problems, which could inflate the associations seen in the study. Although the authors recognize the potential for BMI misclassification, depending on one reporter for all the variables threatens the validity of the findings.

3. The authors combined "Current" diagnoses with "Lifetime" diagnoses into one binary outcome to maintain sample size. This decision complicates a cross-sectional study that connects current BMI with mental health. A child may have received a diagnosis years ago (a lifetime diagnosis) but be in remission now, yet they are analyzed with their current BMI. This mismatch can confuse the results. For example, a child who was depressed three years ago, but is currently healthy and obese, contributes to a positive association that may not be causal in the present.

4. The results show a significant association between obesity and depression for children who experienced "never" or "occasional" bullying. However, the confidence interval for those who faced "frequent" bullying crosses the null. The authors suggest that frequent bullying might build resilience. This idea is controversial and could be dangerous, especially without long-term data to support it. It is also likely that a smaller group size contributed to this finding, or that frequently bullied children show symptoms not measured by the specific anxiety and depression assessments (like acting out or complete withdrawal).

5. Even though this study is cross-sectional, the manuscript sometimes uses causal language. Phrases like "impact of," "escalate the risk," and "disrupt the pathways" suggest a directionality that cannot be established with this data. Reverse causation (for example, depression leading to obesity) is also a valid possibility, as noted in the introduction but not fully addressed in the conclusion.

6. The authors should conduct a sensitivity analysis that separates "Current" diagnoses from "Lifetime" diagnoses. Even if the sample size is smaller, it is crucial to show the odds ratios for Current Depression/Anxiety associations with Current BMI. If the "Current" model does not show significance, this limitation must be clearly stated.

7. The authors should provide the raw counts (both unweighted and weighted) for the "Obese + Frequent Bullying" category. If this sample size is small, the lack of significance (CI crossing 1.0) is likely due to a Type II error (a power issue) rather than a "resilience" effect. The discussion on "frequent bullying building resilience" should be removed or carefully qualified as a statistical artifact unless strong literature supports this claim.

8. The limitations section should be expanded to discuss Common Method Bias beyond just BMI misclassification. It is essential to consider how a parent's mental health status (which acts as a moderator) affects their reporting of the child's experiences with bullying and mental health conditions.

Reviewer #2: I read the manuscript with great care and attention. The authors have satisfactorily addressed the questions posed in the initial submission. The manuscript is now more thorough and easier to read. It is now more appropriate technically. The manuscript may now contribute to the body of scientific literature in its current form, in my opinion.

Reviewer #3: Let the Authors endeavour to implement all the corrections before further submission please.

Reviewer #4: Very nice work. I have a few comments:

1. Line 48 - The LDA/LDD acronyms are appearing for the first time, you should spell it out just like you did for the BMI in line 54.

2. In your results section of the abstract, you should qualify your moderators. For example in line 62, you report maternal mental health when you are actually reporting the poor maternal mental health. The same goes for poor paternal mental health an lots of difficulty making friends.

3. Line 155 - You use SEM for the first time. While I know you are referring to the socioecological model, I think you should still spell it out.

4. Between lines 325 and 327, you conclude that the supplementary table demonstrates there was no significant demographic differences between participants who were included and those who were excluded. I beg to differ. I can see some considerable differences especially for the FPL (bar the 200-399% sub-category) and the Race/Ethnicity category (bar the other sub-category). Unless you performed some statistical significance analysis that shows these differences are not significant, I really do not see the utility of this table.

5. In line 338 and 339, I see you did not adjust for FPL for the LDA but did for the LDD. I am curious about this choice.

6. You mentioned in line 488, 'backed by resilience research' but did not include any source.

7. Between lines 505 and 507, you attempt to explain the reason for the strong link between poor maternal mental health and child depression but offer none for the strong link between poor paternal mental health and child depression. I was wondering what you have to say about that.

**********

7. PLOS authors have the option to publish the peer review history of their article (what does this mean?). If published, this will include your full peer review and any attached files.

**Do you want your identity to be public for this peer review?** If you choose “no”, your identity will remain anonymous but your review may still be made public.

For information about this choice, including consent withdrawal, please see our Privacy Policy.

Reviewer #1: No

Reviewer #2: **Yes:**Gyanesh Kumar Tiwari

Reviewer #3: No

Reviewer #4: No

**********

[NOTE: If reviewer comments were submitted as an attachment file, they will be attached to this email and accessible via the submission site. Please log into your account, locate the manuscript record, and check for the action link "View Attachments". If this link does not appear, there are no attachment files.]

-->Figure Resubmissions:

While revising your submission, we strongly recommend that you use PLOS’s NAAS tool (https://ngplosjournals.pagemajik.ai/artanalysis) to test your figure files. NAAS can convert your figure files to the TIFF file type and meet basic requirements (such as print size, resolution), or provide you with a report on issues that do not meet our requirements and that NAAS cannot fix.

After uploading your figures to PLOS’s NAAS tool - https://ngplosjournals.pagemajik.ai/artanalysis, NAAS will process the files provided and display the results in the "Uploaded Files" section of the page as the processing is complete. If the uploaded figures meet our requirements (or NAAS is able to fix the files to meet our requirements), the figure will be marked as "fixed" above. If NAAS is unable to fix the files, a red "failed" label will appear above. When NAAS has confirmed that the figure files meet our requirements, please download the file via the download option, and include these NAAS processed figure files when submitting your revised manuscript.

Attachment Submitted filename: RE PGPH-D-25-02696_R1.pdf

# Author comment: Author response to Decision Letter 2

**DOI:** 10.1371/journal.pgph.0006462.r004

14 Apr 2026

Attachment Submitted filename: Response to Reviewers_2nd revision.docx

# Peer review: Decision Letter 2

**DOI:** 10.1371/journal.pgph.0006462.r005

**Authors:** Somayeh Hessam

28 Apr 2026

Between the Scales and the Scars: The Interpersonal Moderators of the Association Between Body Mass Index and Parent-Reported Depression/Anxiety Diagnoses in U.S. Children: Evidence from the 2021- 22 National Survey of Children’s Health (NSCH)

PGPH-D-25-02696R2

Dear Dr Kajal,

We are pleased to inform you that your manuscript 'Between the Scales and the Scars: The Interpersonal Moderators of the Association Between Body Mass Index and Parent-Reported Depression/Anxiety Diagnoses in U.S. Children: Evidence from the 2021- 22 National Survey of Children’s Health (NSCH)' has been provisionally accepted for publication in PLOS Global Public Health.

Before your manuscript can be formally accepted you will need to complete some formatting changes, which you will receive in a follow up email. A member of our team will be in touch with a set of requests.

Please note that your manuscript will not be scheduled for publication until you have made the required changes, so a swift response is appreciated.

IMPORTANT: The editorial review process is now complete. PLOS will only permit corrections to spelling, formatting or significant scientific errors from this point onwards. Requests for major changes, or any which affect the scientific understanding of your work, will cause delays to the publication date of your manuscript.

If your institution or institutions have a press office, please notify them about your upcoming paper to help maximize its impact. If they'll be preparing press materials, please inform our press team as soon as possible -- no later than 48 hours after receiving the formal acceptance. Your manuscript will remain under strict press embargo until 2 pm Eastern Time on the date of publication. For more information, please contact globalpubhealth@plos.org.

Thank you again for supporting Open Access publishing; we are looking forward to publishing your work in PLOS Global Public Health.

Best regards,

Somayeh Hessam

Academic Editor

PLOS Global Public Health

***********************************************************

Reviewer Comments (if any, and for reference):

Reviewer's Responses to Questions

**Comments to the Author**

1. If the authors have adequately addressed your comments raised in a previous round of review and you feel that this manuscript is now acceptable for publication, you may indicate that here to bypass the “Comments to the Author” section, enter your conflict of interest statement in the “Confidential to Editor” section, and submit your "Accept" recommendation.

Reviewer #2: All comments have been addressed

Reviewer #3: All comments have been addressed

Reviewer #4: (No Response)

**********

2. Does this manuscript meet PLOS Global Public Health’s publication criteria? Is the manuscript technically sound, and do the data support the conclusions? The manuscript must describe methodologically and ethically rigorous research with conclusions that are appropriately drawn based on the data presented.

Reviewer #2: Yes

Reviewer #3: Yes

Reviewer #4: (No Response)

**********

3. Has the statistical analysis been performed appropriately and rigorously?

Reviewer #2: Yes

Reviewer #3: Yes

Reviewer #4: (No Response)

**********

4. Have the authors made all data underlying the findings in their manuscript fully available (please refer to the Data Availability Statement at the start of the manuscript PDF file)?

The PLOS Data policy requires authors to make all data underlying the findings described in their manuscript fully available without restriction, with rare exception. The data should be provided as part of the manuscript or its supporting information, or deposited to a public repository. For example, in addition to summary statistics, the data points behind means, medians and variance measures should be available. If there are restrictions on publicly sharing data—e.g. participant privacy or use of data from a third party—those must be specified.

Reviewer #2: Yes

Reviewer #3: Yes

Reviewer #4: (No Response)

**********

5. Is the manuscript presented in an intelligible fashion and written in standard English?

PLOS Global Public Health does not copyedit accepted manuscripts, so the language in submitted articles must be clear, correct, and unambiguous. Any typographical or grammatical errors should be corrected at revision, so please note any specific errors here.

Reviewer #2: Yes

Reviewer #3: Yes

Reviewer #4: (No Response)

**********

6. Review Comments to the Author

Please use the space provided to explain your answers to the questions above. You may also include additional comments for the author, including concerns about dual publication, research ethics, or publication ethics. (Please upload your review as an attachment if it exceeds 20,000 characters)

Reviewer #2: I read the manuscript again. I believe the authors have addressed all the queries raised during the initial review. It has improved comprehensiveness and readability of the manuscript. It now has a better technical fit. In my opinion, the manuscript in its current form has the potential to advance knowledge.

Reviewer #3: The Author has done all the necessary corrections needed to proceed for recommendation. Good luck.

Reviewer #4: Kudos to the authors.

1. Please include your p-values in table 2.

**********

7. PLOS authors have the option to publish the peer review history of their article (what does this mean?). If published, this will include your full peer review and any attached files.

**Do you want your identity to be public for this peer review?** If you choose “no”, your identity will remain anonymous but your review may still be made public.

For information about this choice, including consent withdrawal, please see our Privacy Policy.

Reviewer #2: **Yes:**Gyanesh Kumar Tiwari

Reviewer #3: **Yes:**PROF AJAO ADEWALE G.

Reviewer #4: No

**********

Attachment Submitted filename: Final Clean Manuscript.docx
